# Diversification of bacterial genome content through distinct mechanisms over different timescales

**DOI:** 10.1038/ncomms6471

**Published:** 2014-11-19

**Authors:** Nicholas J. Croucher, Paul G. Coupland, Abbie E. Stevenson, Alanna Callendrello, Stephen D. Bentley, William P. Hanage

**Affiliations:** 1 Centre for Communicable Disease Dynamics, Harvard School of Public Health, 677 Huntington Avenue, Boston, Massachusetts 02115, USA; 2 Department of Infectious Disease Epidemiology, St. Mary’s Campus, Imperial College, London W2 1PG, UK; 3 The Wellcome Trust Sanger Institute, Wellcome Trust Genome Campus, Hinxton, Cambridge CB10 1SA, UK

## Abstract

Bacterial populations often consist of multiple co-circulating lineages. Determining how such population structures arise requires understanding what drives bacterial diversification. Using 616 systematically sampled genomes, we show that *Streptococcus pneumoniae* lineages are typically characterized by combinations of infrequently transferred stable genomic islands: those moving primarily through transformation, along with integrative and conjugative elements and phage-related chromosomal islands. The only lineage containing extensive unique sequence corresponds to a set of atypical unencapsulated isolates that may represent a distinct species. However, prophage content is highly variable even within lineages, suggesting frequent horizontal transmission that would necessitate rapidly diversifying anti-phage mechanisms to prevent these viruses sweeping through populations. Correspondingly, two loci encoding Type I restriction-modification systems able to change their specificity over short timescales through intragenomic recombination are ubiquitous across the collection. Hence short-term pneumococcal variation is characterized by movement of phage and intragenomic rearrangements, with the slower transfer of stable loci distinguishing lineages.

## Abstract (web summary)

Populations of the pathogenic bacterium *Streptococcus pneumoniae* consist of distinct co-circulating lineages. Here, the authors show lineages are characterized by particular combinations of stable genomic islands, whereas prophage and restriction-modification systems vary over short timescales.

## Graphical abstract

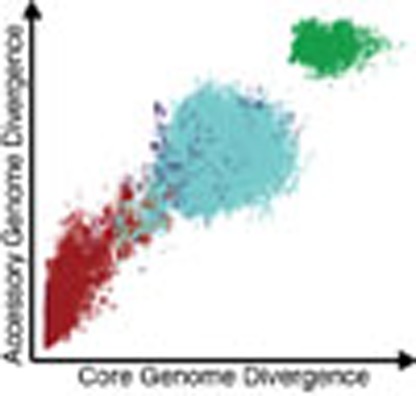

S*treptococcus pneumoniae* is a human respiratory commensal and pathogen in which extensive genetic diversity underlies phenotypic variation in traits such as antibiotic resistance, virulence and antigenic profile. Alongside considerable allelic variation in the core genome, the species contains many ‘genomic islands’ (GIs): genetic loci only found in a subset of the population^1^. Any GI may be transferred between cells through transformation^2^, as pneumococci possess a competence system. Some GIs, referred to as ‘mobile genetic elements’ (MGEs), encode functions that promote their own transfer between cells. At least three types of MGEs have been characterized in *S. pneumoniae*: phage^3^, most commonly of the *Siphoviridae* family^4^; plasmids^5^,^6^, of which just two cryptic examples are known in pneumococci; and integrative and conjugative elements (ICEs), which have played an important role in the spread of antibiotic resistance^7^,^8^.

Horizontal movement of DNA can be limited by bacterial ‘immunity’ mechanisms^9^. Although pneumococci lack CRISPR elements^10^, they do encode restriction-modification systems^11^ (RMSs) that can cleave MGEs when present within the cell as double-stranded DNA. While the pneumococcal competence machinery imports DNA into the cell in restriction-insensitive single-stranded form^12^,^13^, the acquisition of novel GIs by this mechanism necessitates the synthesis of the complementary strand of DNA after integration into the chromosome. If the pattern of modification of the imported DNA differs from that of the recipient's genome, the locus may become vulnerable to endonucleolysis by RMSs that cleave unmodified motifs^2^. Hence, variation in many RMSs affects multiple mechanisms of GI transfer. However, the best-characterized pneumococcal RMSs^11^, *Dpn*I and *Dpn*II, do not inhibit the acquisition of GIs by transformation. *Dpn*I cleaves GATC motifs when fully methylated in double-stranded DNA, while the *Dpn*II RMS cleaves unmethylated GATC motifs, but encodes a methylase that modifies imported single-stranded DNA^2^,^14^. Another factor that has been suggested to affect the movement of sequence is the lineage’s ‘pherotype’, determined by the sequence of the competence stimulating peptide (CSP) pheromone it secretes^15^. CSP is a critical signal in triggering competence for transformation, and multiple sets of cognate signalling peptides and receptors are found in the population^16^. It remains controversial as to whether the exchange of sequence is inhibited by differences in pherotype between pneumococci^17^,^18^.

A recently published set of 616 draft pneumococcal genomes^19^ provides an opportunity to examine the extent of, and processes underlying, variation in gene content within a single population. This systematic collection of isolates carried by children in Massachusetts was previously divided into fifteen monophyletic sequence clusters (SCs), and a sixteenth diverse group of rarer genotypes (SC16), based on variation in the core genome. One monophyletic SC (SC12), composed of atypical unencapsulated pneumococci^20^ that have caused outbreaks of conjunctivitis^21^, was found to be a distinct outlier from the rest of the population. The analysis presented here describes the evolutionary processes that generate this population structure. SCs were found to be characterized by their complement of stable GIs, including those transferred primarily through transformation and conjugation, while changes over shorter timescales frequently represented the consequence of phage transmission and intragenomic recombination.

## Results

### SCs have distinct accessory genomes

**Figure 1:**
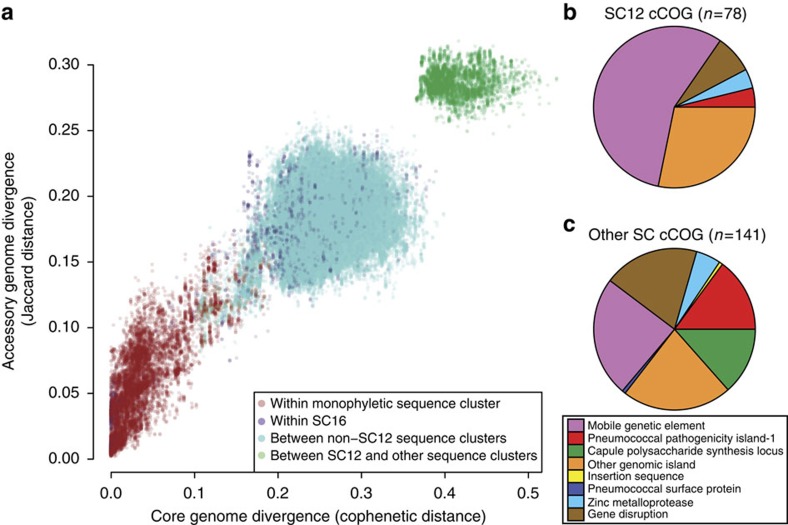
Existence of distinct clusters in the pneumococcal population and the properties of the cCOGs with which they are associated. (**a**) Comparison of pairwise distances between isolates in terms of their core genome divergence, as measured by the cophenetic distance calculated from a maximum likelihood core genome phylogeny^19^, and the difference in their accessory genomes, as measured by the Jaccard distance based on the variation in the COG content of their sequences. Points in red indicate comparisons within monophyletic sequence clusters, while purple points represent comparisons between isolates within the diverse SC16. Points in green indicate comparisons between the atypical unencapsulated isolates of SC12 and other sequence clusters; points in turquoise represent all other comparisons between isolates in different sequence clusters. (**b**) Properties of the COGs characteristic of SC12. The 78 COGs found in >95% of SC12 isolates, and found at a frequency <5% in the other monophyletic sequence clusters, were classified according to their function or location. (**c**) The 141 cCOGs of all other sequence clusters classified in the same manner.

The original analysis of 616 pneumococcal genome sequences identified 5,442 clusters of orthologous genes (COGs), of which around 1,500 were ‘core’ to almost all isolates and around 3,000 were rare^19^. Applying the ‘power law’ method for quantifying the pangenome^22^,^23^ suggested the gene pool available to this population was unbounded (Supplementary Fig. 1). However, this was heavily influenced by numerous rare COGs that individually had little impact on the population structure, and were the most likely to represent false-positive gene predictions. An alternative representation (Fig. 1) showed the distribution of variation across the population using pairwise comparisons between isolates. This revealed three distinct groups of points that suggested differences in gene content were approximately proportional to core genome divergence.

The mainly red group of points nearest the origin of the plot demonstrated isolates within the same monophyletic SC were highly similar in their core and accessory genomes, while the set of turquoise points showed the greater level of divergence between representatives of different non-SC12 SCs (purple points represent comparisons between isolates in SC16). The discontinuity between these two sets of points indicated clonal structure in the population, as higher rates of recombination were predicted to generate a more homogenous distribution (Supplementary Fig. 2). Hence the co-circulating lineages that could be distinguished through their core genomes^24^ also maintained distinct accessory genomes. The set of green points represented comparisons between isolates in SC12 and those in other SCs, highlighting the divergence of SC12 from the rest of the population.

### Potential speciation of atypical genotypes

Genetic loci unique to SC12 seemed likely to explain its distinctive phenotype and disease tropism. To define a set of candidate genes, ‘characteristic COGs’ (cCOGs) were identified in each SC as those COGs found in greater than 95% of genomes in that cluster, while being present in fewer than 5% of genomes in any other monophyletic SC. This identified 78 cCOGs in SC12 (Fig. 1b). Forty-four cCOGs were found within putative MGEs, and a further three were found within the conjugative element-related pneumococcal pathogenicity island 1 (PPI-1)^8^, although the *pit* iron transporter operon within this locus implicated in pathogenesis^25^ was absent from SC12 but present in all other SCs (Supplementary Fig. 3a).

Other non-MGE GIs contributing to SC12 divergence appeared to represent single gain or loss events conserved across the SC. All lacked a functional capsule polysaccharide synthesis (*cps*) locus and conserved a distinct set of large surface proteins (Supplementary Fig. 3b–e). The SC12 isolates also lacked either of the fucose utilization loci, one of which was evident in all other SCs (Supplementary Fig. 4); the conservation of these sequences across deep-branching clades suggested exchange at this locus was not rapid, and therefore a single deletion in an ancestor of SC12 would account for the observed pattern. Analogously, it seemed likely a single acquisition of genes encoding dihydroxyacetone kinases, in place of the pneumococcal histidine triad protein gene *phpA* (Supplementary Fig. 3f), would explain their conservation in SC12 and absence from other isolates in the collection. Hence, the SC12 isolates appeared to be genetically, antigenically and metabolically distinct from the other SCs, and therefore may represent a separate species.

### Clonal association of GI diversity

**Figure 2:**
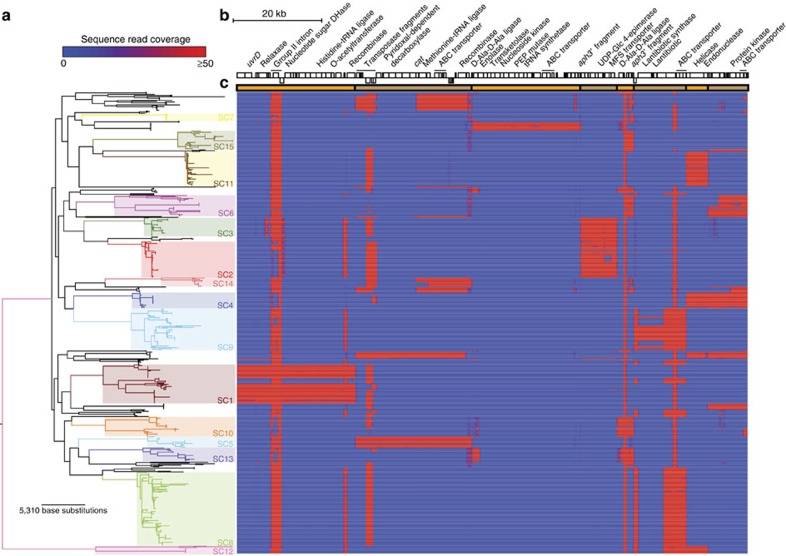
Distribution of PPI-1 3′ variable region sequences across the population. (**a**) The maximum likelihood phylogeny based on the core genome annotated according to the distribution of sequence clusters. (**b**) The set of sequences representing the diversity of the 3′ variable region of PPI-1 across the collection; different reference sequences are demarcated by the alternating orange and brown blocks. Coding sequences that could be annotated based on functional domain information are marked (DHase—dehydrogenase). (**c**) Heatmap representing the distribution of sequence across the population. Each row corresponds to an isolate in the phylogeny. Absence of mapping reads is indicated by blue; red regions indicate read mapping coverage up to a maximum of 50-fold, demonstrating the locus is present in the relevant isolate.

Each of the other SCs had a smaller number of cCOGs (Fig. 1c and Supplementary Table 1). In some cases, these corresponded to putative protein antigens or *cps* genes; for instance, all SC4 representatives expressed capsule type 22F, not found elsewhere in the sample^19^. The other cCOGs showed little similarity in sequence or putative function, although a substantial number were located in the 3′ variable region of PPI-1^8^. Although extensive diversity was observed at this locus across the species, there was little evidence of variation within SCs (Fig. 2), suggesting genes within this locus may underlie lineages’ distinctive traits: at least some allelic variation has previously been associated with differences in virulence in a mouse model of disease^26^. Distinct loci within PPI-1, each ~20-kb long and encoding metabolic genes, were evident in SC1, SC5 and the serotype 3 isolates^27^. However, not all alleles were unique to a SC: SC2 and SC3 shared an ~10 kb gene cassette, a 3.8 kb allele was common to SC6, SC10, SC13 and SC15, and both complete and incomplete versions of a previously described lantibiotic synthesis gene cluster^8^ were found in the PPI-1 loci of SC8, SC9 and SC12. In SC12, these genes were accompanied by a putative RMS, which alone constituted the 3′ variable region of PPI-1 in SC4 and SC11. The read mapping suggested SC4 also possessed the version of the island found in SC6, but in fact these genes were found on an ICE and appear to exemplify the contribution of MGEs to the diversity of sequences within PPI-1^8^,^28^. MGEs themselves accounted for almost a quarter of the cCOGs not associated with PPI-1, suggesting such elements did not necessarily exhibit a high level of mobility, and instead may contribute to the stable differences between SCs.

### Diversification driven by MGEs

The smallest putative mobile sequences previously characterized in pneumococci, three families of short interspersed repeats^29^, were generally stable in frequency within SCs (except for expansion and contraction of boxB tandem arrays), with small differences between lineages (Supplementary Fig. 5). All three families were evident in SC12 at typical frequencies, in contrast to the related species *S. pseudopneumoniae*^30^ and *S. mitis*^31^. Similarly, some types of insertion sequences (ISs) were ubiquitous across the sample, while others exhibited stable associations with particular lineages (Supplementary Fig. 6). Acquisition of novel ISs was observed within SC8: IS*1202* was gained through serotype switching events twice, while IS*Spn*5 was imported as part of ICE ‘scars’^8^. An extensive search for longer MGEs (see Supplementary Methods) identified 2,260 putative MGE-derived genetic loci, with a median length of 31 COGs (range 2–91 COGs). On the basis of their distribution around the chromosome, 16 insertion sites could be robustly identified within the core genome (Supplementary Fig. 7 and Supplementary Methods). As in *Escherichia coli* and *Salmonella enterica*, the majority of the insertion sites (15 of 16) were in intergenic regions despite the high coding densities of bacterial genomes^32^. However, in contrast to these enteric bacteria, all but two insertion sites were closer to the origin of replication than the terminus. As the distance of genes from the replichore boundaries is conserved even more strongly than synteny in pneumococci^33^, this result should apply across the species, although sequence variation prevented the re-identification of three known insertion sites for large conjugative elements that lie close to the terminus of replication (Supplementary Fig. 7).

**Figure 3:**
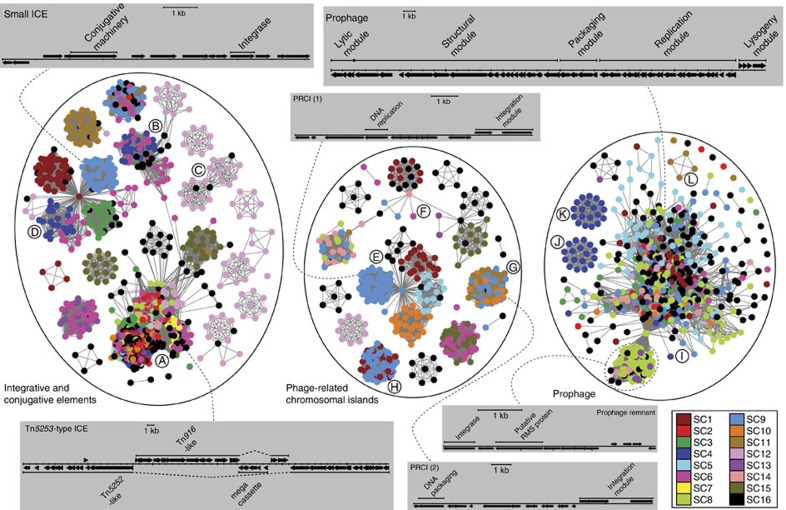
Mobile genetic elements found in the pneumococcal population. The 2,226 MGE sequences identified in the collection that could be classified as derived from a putative ICE, PRCI or prophage are each represented by a node, coloured according to the sequence cluster of the host in which it was found as displayed in the key. These are linked by vertices based on their similarity in terms of COGs using the Mountford index and classified using functional domains that appear characteristic of different MGE types. Clusters of nodes described in the text are annotated with letters. The grey boxes display the annotation of representative nodes, indicated by the dashed lines, from the main clusters in the network.

A network was constructed in which each putative MGE was represented by a node, coloured according to the SC of the host cell, with vertices linking elements determined as being similar using Mountford’s index^34^. This allowed all but 34 putative MGEs to be classified into three groups based on the presence of functional domains (Fig. 3, Supplementary Figs 8,9; Supplementary Methods). The most numerous group (1,083 nodes) represented putative ICEs (Supplementary Table 2). These spanned the full range of detected MGE lengths, likely reflecting the efficiency of conjugation in transferring long segments of DNA between streptococci^35^, permitting modular variation through the insertion or deletion of sequence segments^36^. Hence, these elements are effective vectors for the import of novel DNA into a species. For instance, all antibiotic resistance genes encoded by MGEs were found on ICEs in the component labelled A and B. These consisted of sequences related to Tn*5253*, generated through the insertion of Tn*916*-type elements and other cassettes into Tn*5252*-type elements^8^,^37^ (Supplementary Figs 10,11). Conversely, ICEs in component C did not appear to carry `cargo' genes, but did exhibit extensive similarity to *Streptococcus suis* MGE ICE*Ssu*32457^38^, which contained a cluster of resistance genes not evident in the pneumococcal elements (Supplementary Fig. 12). Hence, in other species, these elements can fulfil the role played by component A and B ICEs in pneumococci.

Component C was one of the six ICE network components predominately associated with SC12, and in this case appeared to represent a conserved GI distinguishing these isolates from the rest of the population. Such sharing of MGEs through recent common ancestry (that is, vertical transmission of the MGE) was indicated by these cliques of highly connected nodes within the same SC. Conservation of ICEs has been observed in multidrug-resistant lineages in which the ICEs encoded resistance genes^37^,^39^,^40^, but many examples identified here, such as those in component C or the Tn*5252*-type MGE similar to ICE*Sp*PN1^28^ found in SC6 (Supplementary Fig. 11), lack such obviously beneficial cargo genes. In some cases, the importance of vertical transmission to the spread of some MGEs may reflect the absence of modules encoding the machinery needed for horizontal mobility. Examples were evident in component D (Supplementary Fig. 13), the longest representative of which appeared potentially mobile, whereas the shorter members may have lost some of the machinery for transfer between cells^41^.

The second group of MGEs, accounting for 471 nodes, likely represents phage-related chromosomal islands (PRCIs), mobilized in *cis* by ‘helper’ prophage^42^. First identified as ‘pathogenicity islands’ carrying superantigens in *Staphylococcus aureus*^43^, these pneumococcal examples encoded a high proportion of sequences for which no robust functional prediction could be made. Representatives from components E and F exhibited similarity with the *Streptococcus pyogenes* PRCI *Spy*CI1 (Supplementary Figs 14,15) and were typically between 8 and 15 kb in length with putative integrase and regulatory genes transcribed in one direction and a DNA primase gene transcribed in the opposite direction. Representatives from component G were similar in size and genetic organization, with an integration module that showed limited similarity with the enterococcal PRCI *Ef*CIV583^44^ (Supplementary Fig. 16). The most unusual representatives were in component H, in which the putative integrase and primase genes were linked to a central, transposase-flanked portion that closely matched a GI from *Streptococcus mutans* LJ23 (Supplementary Fig. 17). However, there was generally little evidence of the ICE-type modular evolution: PRCIs exhibited less variation in size (Supplementary Fig. 8), and the same core set of functions tended to be conserved between them. Sequence variation was instead mosaic in nature, with the level of sequence divergence between representatives changing at breakpoints that varied between elements, likely representing the consequence of homologous recombination.

Exhibiting a similar mosaic pattern of sequence variation were prophage, the third group of 672 nodes. These generally conserved a distinctive module order, and had a consistent orientation across the five insertion sites containing full-length prophage in which the genes active during MGE replication were aligned with the strong coding bias of the pneumococcal genome, akin to the ‘polarization’ seen in enteric bacteria^32^. In marked contrast with ICEs and PRCIs, few instances of prophage being stably associated with a lineage were observed, as implied by the connectivity within component I (Supplementary Table 2). Notable exceptions to this trend were evident: isotypes of prophage φOXC141, independently observed to be stably associated with the serotype 3 genotype predominant in Massachusetts^19^,^27^, were identified in the expected hosts (Supplementary Fig. 18). These viruses were within component I, which encompassed the previously described diversity of pneumococcal phages^4^. Similarly conserved between related isolates were two atypical phage: one similar to *Enterococcus faecalis* V583-pp1^44^ present in all but four members of SC4 (components J and K; Supplementary Fig. 19), and another similar to *S. oralis* prophage φPH10 present in five SC11 isolates (component L; Supplementary Fig. 20). Yet, the largest set of nodes that showed stable association with host SCs was found within component I; these represented a GI identified in the multidrug-resistant PMEN1 lineage^8^ that likely represented a ‘prophage remnant’ that has lost its mobilization machinery (Supplementary Fig. 18). Such degradation of an MGE can occur when selection acts to conserve a beneficial cargo gene^45^; the only candidate in this instance was a coding sequence (CDS) with a functional domain associated with RMSs, suggesting this gene may have been preserved to protect against other MGEs.

### Potential barriers to sequence exchange

**Figure 4:**
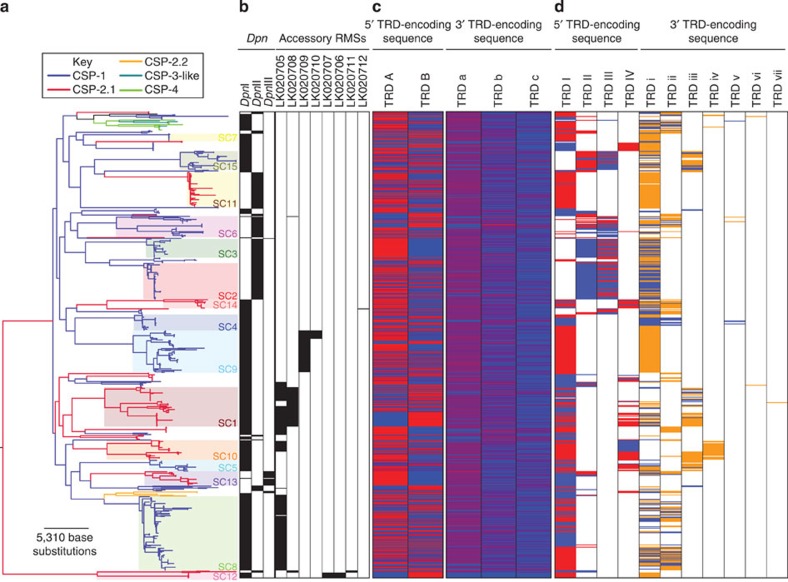
Mechanisms potentially affecting GI transfers. (**a**) Maximum likelihood phylogeny based on the core genome annotated according to the distribution of sequence clusters. The branches of the phylogeny are coloured according to a maximally parsimonious reconstruction of CSP pherotype. The ‘CSP-3-like’ sequence was identical to the previously described CSP-3 (ref. 16) but lacking an FNIFNF peptide. (**b**) Variation in accessory RMSs. The columns to the left indicate which of the three RMSs is present at the *dpn* locus by black bars in the appropriate rows. The eight columns to the right indicate the presence of other putative accessory RMSs, as inferred from the distribution of the relevant methylase COGs. Columns are labelled with the accession code of the sequence in Supplementary Table 4, with black bars again indicating the presence of an RMS in the corresponding isolate. (**c**) Variation in the *ivr* locus. The left columns show reads corresponding to the 5′ part of the full-length *spnIVRhsdS* gene assigned to the two alternative 5′ TRD-encoding sequences A or B. The heatmap indicates the proportion of reads corresponding to the *spnIVRhsdS* gene that matched each allele, with red indicating a higher proportion and blue a lower proportion. The right columns show reads likely corresponding to the 3′ part of *spnIVRhsdS* assigned to the three alternative TRD-encoding sequences a, b or c. (**d**) Variation in the *tvr* locus. Eleven different *spnTVRhsdS* TRD-encoding sequences were identified across the population. When the TRD-encoding sequence was present as part of a full-length CDS, the cell is coloured red, if the TRD was found in the 5′ half (these are labelled with uppercase Roman numerals), and orange, if found in the 3′ half (these are labelled with lowercase Roman numerals). Where the TRD-encoding sequence was present as a lone fragment, the corresponding cell in the grid is coloured blue. Empty cells indicate the TRD-encoding sequence was absent from the corresponding isolate.

The apparently high rate of phage transmission suggested there would be strong selection for mechanisms that prevented infection with these viruses, which may also inhibit the exchange of other GIs. RMSs seemed likely to play such a role, and 11 candidate RMSs were identified using Pfam domains^46^ (Supplementary Tables 3 and 4). Three of these were present at the *dpn* locus, of which two were the previously characterized *Dpn*I and *Dpn*II systems^11^. The only example of switching between these two systems within a SC occurred on the long branch within SC12 (Fig. 4b). The one other change at the *dpn* locus within a SC involved replacement of a Type II RMS (designated *Dpn*III and represented by SPN23F18640-18650 in the genome of *S. pneumoniae* ATCC 700669 (ref. 8)) present in all but one isolate of SC13, in which it had been replaced by *Dpn*I. *Dpn*III likely targets a different motif to *Dpn*I and *Dpn*II, both sensitive to adenine methylation^11^, as functional domain information suggested the *Dpn*III RMS modified cytosine bases.

As both *Dpn*I and *Dpn*II do not prevent the uptake of GIs by transformation^2^, but are likely to be similarly effective against MGEs found as double-stranded DNA forms in the cell, it was unsurprising to find that the accessory genome diversified at approximately the same rate in isolates carrying either system (Supplementary Fig. 21). However, there was also little difference in the equivalent rate estimated from those isolates carrying *Dpn*III, which appeared to be a typical Type II restriction system. Furthermore, increasing numbers of non-*Dpn* accessory RMSs also did not appear to affect the rate of accessory genome diversification, despite these Type I and II systems being potentially able to cleave MGEs or any GIs imported by transformation (Supplementary Fig. 22). Despite their apparent lack of effect on the plasticity of genome content, these accessory RMSs exhibited a similar level of conservation across clades as the *Dpn* systems (Fig. 4b).

The absence of an observed effect may reflect the influence of other aspects of the transformation mechanism. One candidate was pherotype, which was also conserved across deep-branching clades. All 15 monophyletic SCs were uniformly associated with either CSP-1 or CSP-2, with no isolates having acquired the rarer pherotypes or switched between the more common types (Fig. 4a) despite requiring a change within the range of commonly observed transformation events^47^. One explanation is that inter-pherotype exchange of sequence is infrequent^18^. However, any inhibition of exchange between the pherotypes does not appear to substantially affect their relative rates of recombination. No significant difference was observed in the rate of diversification through homologous recombination relative to point mutation between SCs of the two common pherotypes (Wilcoxon rank-sum test of previously calculated *r*/*m* values^19^, *N*=15, W=20, *P*=0.46), and no substantial difference in the relative rate of accessory to core genome diversification could be identified between them (Supplementary Fig. 23).

### Rapid RMS variation through DNA inversion

**Figure 5:**
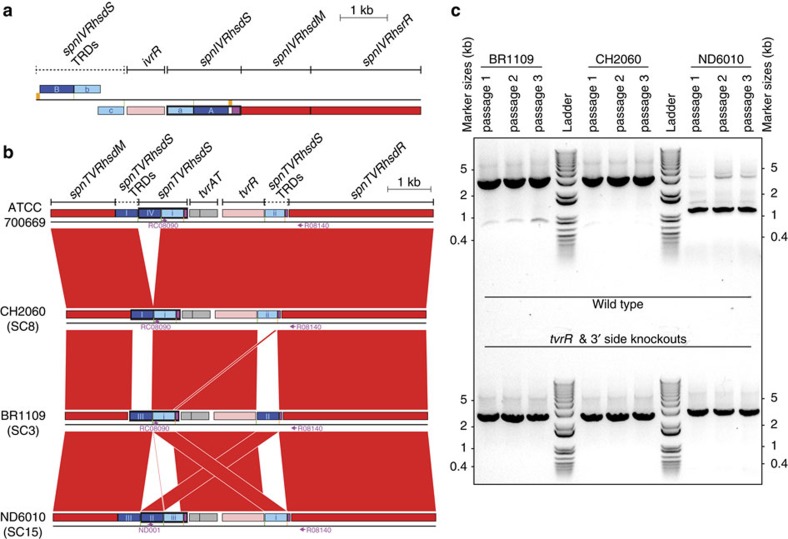
Structures of the RMS loci varying through intragenomic recombination. (**a**) Structure of the *ivr* locus in *S. pneumoniae* R6 and (**b**) structure of the *tvr* locus in *S. pneumoniae* ATCC 700669. In both cases, the CDSs encoding the methylases (*hsdM* genes) and endonucleases (*hsdR* genes) are coloured red, and the CDSs encoding the recombinases are coloured pink. The components of the specificity subunit CDSs are coloured differently: the 5′ TRD-encoding sequences are dark blue, the 3′ TRD-encoding sequences are light blue, and invariant regions are purple. TRD-encoding sequences are labelled as in Fig. 4. Full-length specificity subunit genes, containing a representative of each component type, are boxed. The sets of repeats on which the recombinases may act are indicated by the orange and green boxes. In the *tvr* locus of *S. pneumoniae* ATCC 700669, the two grey CDSs represent *tvrAT*, encoding a putative toxin–antitoxin system. This sequence is aligned with other *tvr* loci encoding functional *spnTVRhsdS* genes. Regions of sequence similarity between loci are indicated by red bands. The positions of primers are indicated with purple arrows. (**c**) Variation in the *tvr* locus through intragenomic recombination. For each Massachusetts isolate in (**b**) (and their corresponding mutants in which the 3′ end of the locus has been replaced), a single colony was serially passaged in broth three times. DNA was extracted from each passage, and the arrangement of the *tvr* locus assayed by PCR amplification with the primers labelled in (**b**) and an extension time of 60 s. The ladder used was the 1 kb Plus Invitrogen ladder, with the darkest band corresponding to a size of 1.65 kb. In each case, the expected size of the band from the native *tvr* locus was >3 kb, with shuffling of the TRD-encoding sequences expected to result in *tvr* locus arrangements that generated smaller bands.

The lack of a detectable impact of either accessory RMSs or pherotype on the rate of genome content diversification indicated there may be an alternative mechanism inhibiting the spread of GIs. Two candidates were ‘core’ RMSs that were ubiquitous in the sampled population (Figs 4 and 5). Both of these were Type I RMS loci containing multiple sequences encoding different DNA-binding target recognition domains (TRDs) of specificity subunits together with a recombinase. One of these loci encoded TRDs on both strands of the genome (Fig. 5a), and had previously been demonstrated to undergo rearrangements through sequence inversion in *S. pneumoniae* TIGR4 (ref. 48). This phase variation resulted in five TRD-encoding sequences being combined into up to six different full-length genes, each encoding a putatively functional Type I RMS specificity subunit formed of two TRDs. This region was denoted the ‘inverting variable restriction’ locus (*ivr* locus), with the specificity subunit encoded by the *spnIVRhsdS* gene. The rapid variation in the composition of *spnIVRhsdS* across the sequenced collection (Fig. 4c) was hypothesized to be driven by intragenomic recombinations catalysed by the recombinase encoded by *ivrR* within the *ivr* locus^48^. Hence, *ivrR* was disrupted using an antibiotic resistance marker to stabilize three different versions of *spnIVRhsdS* generated by intragenomic recombination during routine culturing of *S. pneumoniae* R6 (ref. 49).

The mutants were characterized by SMRT sequencing, with *de novo* assemblies confirming that each had a different *spnIVRhsdS* allele (Supplementary Fig. 24). The mutant with the same *spnIVRhsdS* sequence as the R6 genome (composed of the TRDs denoted Aa) was found to have adenines methylated at the N6 position in three motifs. Two of these, TCGAG and TCTAGA (underlined adenines were methylated; Supplementary Table 5), likely represented the activity of two Type II RMSs. Twenty CDSs in the *S. pneumoniae* R6 genome matched the RMS-associated functional domains listed in Supplementary Table 3. The most likely candidates for causing these methylation patterns were SpnIM, an ‘orphan’ methyltransferase encoded by a CDS adjacent to an endonuclease pseudogene, predicted to target the TCTAGA motif^50^; and Spr1102, which appears to form a functional Type II RMS with Spn1103 (orthologous with the accessory RMS with accession code LK020705 in Fig. 4). The third motif, CAG(N)_8_TTYG, was bipartite and likely to represent the activity of the Type I RMS encoded by the *ivr* locus. SMRT sequencing of the second mutant, in which the 3′ region of the *spnIVRhsdS* gene had been switched by an inversion such that it was composed of TRDs Ab, found the same Type II RMS motifs and an altered Type I motif, GAA(N)_9_TTYG. The maintenance of the 5′ half of the *spnIVRhsdS* allele was consistent with the conservation of the TTYG component of the original motif. Correspondingly, SMRT sequencing of a third mutant with a Ba *spnIVRhsdS* allele, in which only the 5′ region of *spnIVRhsdS* differed from allele Aa, identified a bipartite methylated motif of CAG(N)_7_GTG; this preserved the CAG component of the original motif, while nevertheless again altering the system’s overall specificity.

### Rapid RMS variation through DNA translocation

In contrast with the *ivr* locus described above, the TRD-encoding sequences at the second ‘core’ RMS (the SP_0886-SP_0892 region of the *S. pneumoniae* TIGR4 genome) were all on the same strand (Fig. 5b). In many isolates, apparently functional specificity subunit genes were formed through the combination of two TRDs, as at the *ivr* locus. Alignments of the locus in closely related members of sequence type 3280 (ref. 19) suggested that ‘shuffling’ of TRDs occurred through lateral translocation of DNA (Supplementary Fig. 25); PCR amplification confirmed this was genuine variation and not an assembly artefact (Supplementary Fig. 26). This was unlikely to represent spontaneous, irreversible mutation because isolates in SC2 and SC3 apparently alternated between two different forms (Fig. 4d and Supplementary Fig. 27). Rather, the changes were likely catalysed by the putative recombinase, TvrR, encoded by this locus, henceforth termed the ‘translocating variable restriction’ (*tvr*) locus. These alterations would likely involve excision and re-integration of DNA; the putative TvrTA toxin–antitoxin system may select against failure to re-insert the gene cassette during rearrangements, as these systems are likely to be effective in stabilizing such dynamic genetic loci. To test whether variation in this locus could occur through intragenomic recombination, individual colonies from three different isolates, each of which had a different full-length *spnTVRhsdS* gene (Fig. 5b), were serially passaged in broth three times. A PCR was designed to amplify an ~3 kb product from the ‘native’ version of the locus, which could also detect rearrangements through the amplification of shorter products as the consequence of a primer binding site within a TRD-encoding sequence being shuffled closer to the 3′ edge of the locus (Fig. 5c). In the case of CH2060, only the ~3 kb product was clearly observed, suggesting any rearrangements were rare in this isolate; with BR1109, a shorter band became prominent over the time course, suggesting infrequent rearrangement; whereas the variant locus was easily detectable in ND6010 after a single night's growth. None of these shorter bands were observed following the replacement of the 3′ end of the locus, including *tvrR*, with a kanamycin resistance marker (Supplementary Fig. 28). Again, the high rate of this mechanism was reflected by the extensive variation in *spnTVRhsdS* observed across the population (Fig. 4d). These data indicated that the ‘shuffling’ of *spnTVRhsdS* configurations was rapid, commonly occurring within SCs, whereas the horizontal acquisition of new *spnTVRhsdS* TRD-encoding sequences was much less frequent.

*S. pneumoniae* R6 lacks a full-length *tvr* locus specificity subunit gene (*spnTVRhsdS*), hence the absence of any corresponding signal from the previous SMRT sequencing data. To determine whether the system was active when a full-length *spnTVRhsdS* allele was present, the three *tvr* loci in which the 3′ end had been replaced with a kanamycin resistance marker were introduced into the *S. pneumoniae* R6 Aa mutant. When the *tvr* locus from isolate BR1109 was introduced, the same methylation motifs were detected as in the original Aa mutant, with no evidence of another RMS being active. This may be the consequence of a small truncation of the methylase subunit, or a low specificity, or efficiency, of methylation. However, introducing the loci from isolates CH2060 and ND6010 resulted in the detection of both the Type II RMS and *ivr* locus-associated motifs, along with an additional Type I methylation motif (Supplementary Table 5): GATA(N)_6_RTC in CH2060 and GGA(N)_7_TGA in ND6010. Hence, the *tvr* locus encodes an RMS with a specificity apparently determined by the sequence of *spnTVRhsdS*, a gene that can vary through intragenomic recombination.

## Discussion

The observation of distinct co-circulating lineages, as defined by the core genome, is often assumed to mirror selectively important differences in gene content. While pneumococci belonging to the same lineage are more likely to share accessory genome loci, this is generally not the result of lineages maintaining large numbers of unique genes; instead, they are characterized by combinations of stable, individually common GIs. The SC12 isolates were an exception in this population. They appear to be adapted to a different, although likely overlapping, ecological niche and may merit recognition as a novel species.

**Figure 6:**
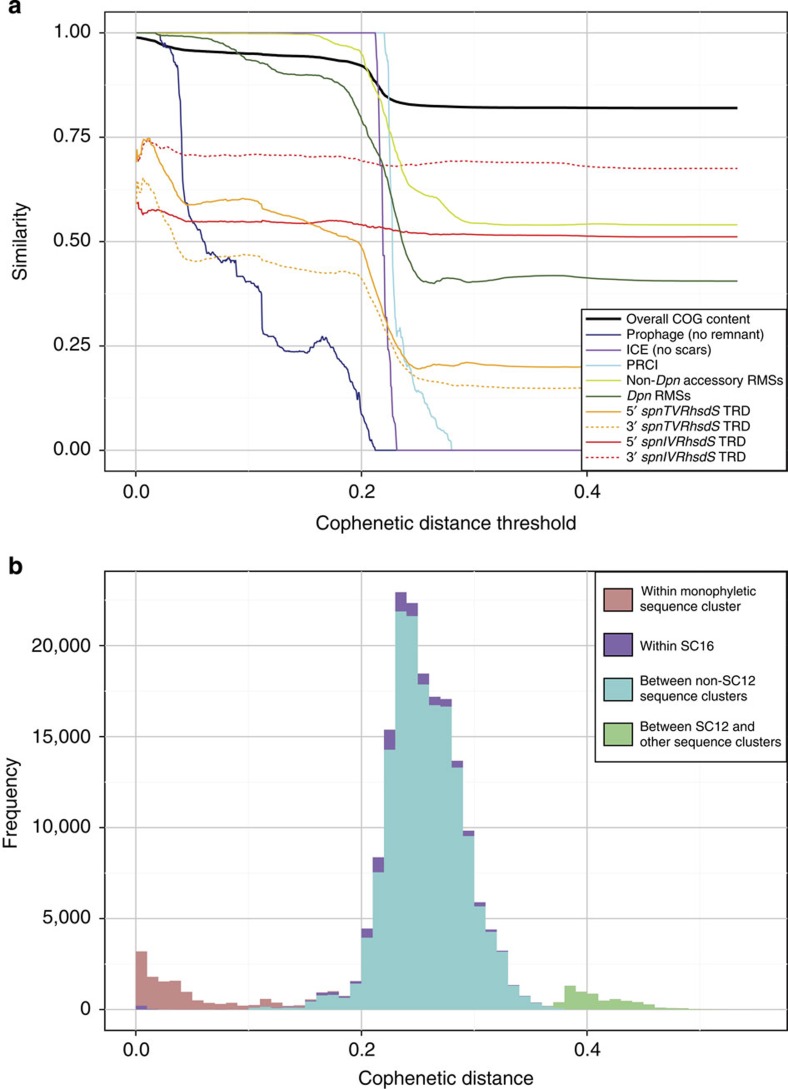
Dynamics of the accessory genome across the population. (**a**) Rate of change in different components of the accessory genome. The horizontal axis represents a threshold maximum cophenetic distance separating isolates, based on the core genome maximum likelihood phylogeny; the vertical axis represents the similarity observed in different aspects of the genome when considering all pairwise comparisons below this cophenetic distance threshold. The black line uses the median Jaccard similarity metric to trace the change in overall COG content between isolates. The blue and purple lines represent the median value of a similar metric calculated only using the subset of COGs characteristic of different MGE classes. Prophage-associated COG content (excluding the prophage remnant GI) diverged considerably within sequence clusters, indicating these MGEs are relatively transiently associated with pneumococcal hosts. By contrast, PRCI and ICE content (excluding the ICE ‘scars’) were stable within sequence clusters, but varied substantially between them. When considering the distribution of RMSs, each pairwise comparison was coded one where both isolates shared the same profile (as calculated from the data in Fig. 4), and therefore the system could not be effective in preventing an MGE transmission, and zero otherwise. In the case of the *ivr* locus, the TRDs most commonly predicted to form the *spnIVRhsdS* gene were used to calculate this metric; in the case of the *tvr* locus, the profile of all TRDs at this locus, including whether or not they were present in a full-length *spnTVRhsdS* gene, was used. The plotted lines show the proportion of pairwise comparisons in which isolates had identical profiles based on the same core genome cophenetic distance thresholds as for the similarities in terms of COGs. This found the *dpn* locus and other accessory RMS to be conserved over relatively long evolutionary timescales, whereas the *ivr* and *tvr* loci were divergent between even very closely related isolates. (**b**) The distribution of pairwise cophenetic distances, calculated from a maximum likelihood core genome phylogeny^19^, represented as a histogram.

Not all GIs were stable; different types demonstrated different dynamics across the population, as illustrated by Fig. 6. The black line traces the general decline in gene content similarity from focusing only on near-identical isolates to comparing the entire collection. This partly reflects the low rate at which GIs primarily depending on transformation for their mobility were exchanged, as exemplified by the conservation of PPI-1 alleles (Fig. 2), genes involved in sugar metabolism (Supplementary Fig. 4) and capsule type^19^ within SCs. Other lines trace the divergence assessed using only the subset of COGs associated with the three large MGE types (Supplementary Methods). These show that PRCIs and ICEs were stable within SCs, but diverged between them. Such conservation may reflect these MGEs providing an advantage to their host, although few examples of potentially beneficial cargo were identified. Nevertheless, these MGEs appear to be reliant on vertical transmission for their success, and therefore the clonal dissemination of ICE-associated antibiotic resistance^37^,^39^,^40^ can also be thought of not only as hosts maintaining selectively advantageous MGEs, but also as MGEs providing a benefit for their long-term host.

In contrast to such a ‘symbiotic’ long-term association between MGEs and hosts, prophage were much less stable (Fig. 6). The typically destructive transmission mechanism of phage imposes a strong selective cost on host cells, meaning isolates carrying prophage are likely to be selected out of the population comparatively quickly. This lowered vertical transmission through inheritance necessitates that phage have an elevated rate of horizontal movement between cells^51^. As RMSs are effective at inhibiting horizontal transmission, the benefit they confer on their host cell is likely to be high if cells are frequently being infected by phage, as in this population. Yet, this inhibition is contingent on the donor and potential recipient harbouring different RMSs. Hence, RMSs can be highly effective at preventing the spread of MGEs if diverse across the population; however, this did not appear to be the case for the accessory RMSs in this collection, as typified by the *dpn* locus only having two common alleles. This contrasts with the extensive variety of prophage from which variants may emerge able to evade such defences. Furthermore, the observed stability of RMSs on non-MGE GIs (Fig. 6) implied they were exchanged less frequently than phage. This makes it difficult to conceive how they might co-evolve at the same rate as phage, and also makes them unlikely to be effective in preventing transmission between isolates of the same SC. Hence, the maintenance of these RMSs may partly reflect persistence as ‘selfish’ elements^52^.

Unlike GI exchange by transformation, the speed of intragenomic recombination is not limited by the need to encounter a suitable donor cell, and hence it can facilitate adaptation over short timescales^53^. Rapid intragenomic changes affecting RMSs have previously been described in other species, through phase variation produced by DNA inversion^54^,^55^, variation in the length of tandem repeat arrays^56^ and homopolymeric tracts^57^. The rate of diversification facilitated by the *ivr* and *tvr* loci means they are likely to be effective at preventing phage transmission in clonally related cell populations, as is evident from the high levels of diversity seen at these loci even between closely related isolates (Fig. 6). Consistent with such an anti-phage activity, of the three motifs associated with the *ivr* locus, the most frequent was found at a density of one site per 2.3 kb in the subset of COGs associated with prophage, with the least frequent was present at a density of one per 16 kb. The motifs associated with the *tvr* locus were found at densities of one per 3.8 kb and one per 5.4 kb in the same sequences. As complete prophage are typically over 30 kb in length^4^, these systems should frequently be effective at preventing viral infection.

Hence, closely related pneumococci are most likely to be distinguished by shuffling of these variable RMSs and their complement of prophage. However, the reversible nature of the RMS alterations means that once an equilibrium level of diversity is reached through intragenomic recombination, it does not tend to increase (Fig. 6), with the exception of transfer of *spnTVRhsdS* TRD-encoding sequences between isolates (Fig. 4d). Similarly, the association of prophage with different lineages is comparatively transient. Hence, the fast movement of phage needed to overcome their relatively low rate of vertical transmission, and the rapid intragenomic recombination that diversifies RMSs likely to inhibit viral infection, do not accumulate to cause ever-greater differences over time. Instead, the infrequent transfer of more stable GIs accounts for the distinctive characteristics of different lineages. It seems likely that the slow pace of such exchanges may partly represent a consequence of the activity of the *ivr* and *tvr* loci, given their broad distribution across the species. For instance, the impact of RMSs on ICE transfer may be inferred from the presence of the *ardA* gene, encoding a DNA-mimicking anti-Type I restriction protein, on Tn*916*-type sequences^58^. Hence the fast, transient ‘microevolutionary’ changes observed within clonal populations can be distinguished from the infrequent ‘macroevolutionary’ events that result in the stable differences between lineages.

## Methods

### Analysis of COG distribution

The COGs and SCs used in these analyses were defined previously^19^. The power law pangenome model^22^,^23^ was fitted to the output of 1,000 replicates in which all 616 isolates were sampled in a random order using R^59^. The comparison of cophenetic and Jaccard distances was achieved using distance matrices calculated with the VEGAN^60^ and APE^61^ packages. When comparing the rates of accessory genome divergence for different subpopulations, plots were generated using only distances between isolate pairs that were concordant for the trait being studied.

Functional annotation was generated for each COG through scanning a representative protein, selected to be of median length, for Pfam^46^ functional domains using HMMer^62^. Characteristic COGs were identified as those COGs present in >95% of the tested monophyletic SC isolates and absent from at least 95% of isolates in other monophyletic SCs. This made allowance for a low rate of assembly or gene prediction error. These cCOGs were then classified using the Pfam annotations and the genomic location of the COG, as ascertained using BLAT for nucleotide alignment^63^, followed by inspection with Artemis and ACT^64^.

### Analysis of GI distribution

For the variable region of PPI-1 and the two fucose utilization gene clusters, the identified alleles represent a manually curated set that were concatenated to form a reference sequence. Repeats were excised from these loci to avoid misleading mapping artefacts. The raw Illumina reads for each isolate were then mapped against this reference using BWA^65^ with standard settings to produce a coverage plot. This was converted to a heatmap using Biopython^66^.

### Simulation of lineage coexistence

A simple simulation was used to test how the observed coexistence of distinct SCs inferred from the accessory genome distribution and core genome phylogeny would be affected by different levels of recombination. A discrete step forward time Wright–Fisher simulation was applied to a population of 1,000 sequences, initially composed of a random sample of 15 different genotypes, based on the number of monophyletic SCs identified in the genome collection. Every sample was represented by 100 ‘core’ loci, and 100 ‘accessory’ loci, each of which was biallelic. In each generation, each individual independently acquired a mutation at a single core locus with a probability of 0.1, or a single accessory locus, also with a probability of 0.1. In addition, with a probability of *r*, a given individual underwent recombination with a randomly selected donor. This involved each locus of the core and accessory genome being independently acquired from the donor with a fixed probability of 0.025 in this simulation (equating to an import of around 50 kb in a pneumococcus). The next generation of 1,000 sequences were then selected from the population at random with replacement, with each simulation run for 10,000 generations. Finally, a neighbour-joining tree was generated from the core loci using APE^61^, and the cophenetic distances plotted against the accessory genome dissimilarity (calculated as a Jaccard distance) as in Fig. 1. Qualitatively similar results were obtained for different mutation rates and numbers of generations.

### Detection and classification of MGE

Short interspersed repeat sequences were detected using hidden Markov models as described previously^29^. Analysis of the distribution of ISs involved constructing a reference sequence from the ISs found to be associated with pneumococci in the ISFinder database^67^ and analysing the distribution of sequence by read mapping as for GIs. The identification and classification of multi-gene MGEs is described in Supplementary Methods and Supplementary Table 6. BLAT^63^ was used for alignment of translated nucleotide sequences; these were displayed using ACT^64^. Accession codes for MGE assemblies are listed in Supplementary Table 7.

### Detection of RMSs

Fifty-eight COGs were identified through searching the overall set for those containing one of the Pfam^46^ domains listed in Supplementary Table 3. Representatives of each example were then manually investigated using ACT, and functional loci selected on the criteria that they contain apparently full-length endonuclease and methylase genes (as well as a specificity subunit gene, if a Type I system). These have been submitted to the ENA with accession codes listed in Supplementary Table 4.

### Ascertaining the arrangement of *ivr* loci *in silico*

For each isolate, the Illumina reads were mapped to the reverse complement of the region defined by coordinates 454708-456366 in the genome of *S. pneumoniae* R6 [EMBL accession code: AE007317]^49^, corresponding to the complete spr0449 *spnIVRhsdM* CDS and invariant 5′ region of the spr0448 *spnIVRhsdS* CDS. The unmapped pairs of those reads mapped in the correct orientation for providing information on the downstream region should correspond to the 5′ variable region of *spnIVRhsdS*. Consequently, the number of uniquely mapping reads with at least 95% similarity along their full length to each of the two alternative 5′ sequences (A and B in Fig. 5a) found in the locus were tallied, and their relative frequencies displayed as a heatmap in the first pair of columns in Fig. 4c. Seven isolates had ten or fewer reads matching the two *ivr* locus 5′ TRD sequences in total; *de novo* assemblies confirmed these isolates had complete, or partial, deletions of the *ivr* locus, and consequently all *spnIVRhsdS* TRD sequences were set as having zero coverage in these isolates.

The 5′ TRD-encoding sequence most frequently found immediately downstream of the *spnIVRhsdM* CDS was then used as the reference sequence for a second round of mapping, as appropriate for each isolate. In this case, the unmapped read pairs downstream of the mapped reads should correspond to the 3′ TRD-encoding sequence of the *spnIVRhsdS* gene (a, b and c in Fig. 5a) most commonly found adjacent to this 5′ TRD-encoding sequence. The relative frequencies of these reads with at least 95% similarity along their whole length to each of the three 3′ TRD-encoding sequence were displayed as the three adjacent columns in Fig. 4c. Ten isolates had fewer than ten reads mapping to the 3′ TRD-encoding sequences. *De novo* assemblies confirmed these corresponded to the clade of seven isolates within SC12, which shared a deletion that eliminated all of the 3′ *ivr* locus TRD-encoding sequences, and a further three isolates across the collection that suffered from deletions or rearrangements at the locus that explained this lack of matches. All 3′ TRD-encoding sequences of *spnIVRhsdS* were set as having zero coverage in these isolates.

### Ascertaining the arrangement of *tvr* loci *in silico*

A single COG (CLS00804) corresponded to the majority of the *tvr* locus TRDs. Every member of this COG was scanned for the Pfam domain Methylase_S (PF01420), found in single copy in *spnTVRhsdS* TRD-encoding sequences and in two copies in putatively functional *spnTVRhsdS* genes. The amino-acid sequences corresponding to this domain were extracted from each CDS, aligned using MUSCLE^68^ and clustered using BAPS^69^. This identified 11 different groups of sequences, which could then be classified as corresponding to 5′ or 3′ TRD-encoding sequences based on the order of domains within putatively functional CDSs. The distribution of these sequences across the population is shown in Fig. 4d.

### Ascertaining *ivr* and *tvr* orientations by PCR

To confirm the orientations of the *ivr* and *tvr* loci by PCR, isolates were cultured overnight in THY broth (Todd Hewitt broth containing 0.5% yeast extract), and their DNA extracted using DNeasy kits (Qiagen). In each reaction, 50 ng of genomic DNA was used as the template for PCR amplification with the specified primers (Supplementary Table 8) using the OneTaq DNA polymerase and appropriate buffer (NEB). Product elongation was performed at 48 °C for a time commensurate with the expected product length. Reaction products were separated by agarose gel electrophoresis.

To produce the time courses shown in Fig. 5c, each of the three isolates CH2060, BR1109 and ND6010 were streaked out on blood agar plates and a single colony transferred into 1 ml of THY broth. Cultures were grown at 37 °C in 5% CO_2_ for 24 h, at which point 100 μl was transferred into a fresh 1 ml of THY medium. This passage was repeated serially two further times for each isolate. DNA was extracted from the broth remaining after inoculation of the next culture using a DNA purification kit (Qiagen). PCR amplification used the primers RC08090 and R08140 for CH2060 and BR1109 (and the corresponding knock out mutants), and primers ND001 and R08140 for ND6010 (and the corresponding knock out mutant), and used the conditions described above except that the extension time in the thermocycle was 60 s, to increase the sensitivity for detection of shorter products generated by rearrangement of the locus.

### Construction of *S. pneumoniae* R6 *ivrR* knockouts

To disrupt the *ivrR* recombinase of *S. pneumoniae* R6 and thereby stabilize the locus in different orientations, the two ~500 bp halves of the recombinase gene were separately amplified using the primer pairs R6hsdSL and Lint, which added an *Apa*I site, and R6hsdSR and Linr, which added a *Bam*HI site. The *ermCB* resistance marker was then amplified using template DNA from a macrolide-resistant PMEN1 isolate with primers ermBF and ermBR, which added *Bam*HI and *Apa*I sites onto the construct, respectively. DNA products were purified by agarose gel electrophoresis, then digested with *Apa*I (NEB) at room temperature for 1 h, or with *Bam*HI (NEB) at 37 °C for 1 h, as appropriate. The three digestion products were purified with a DNA Purification Kit (Qiagen) and mixed in equimolar proportions for ligation with T4 ligase (NEB) at 4 °C for 24 h. Full-length ligation products were then amplified using primers R6hsdSL and R6hsdSR; this allowed a product around 3 kb in length to be purified through agarose gel electrophoresis. This construct was then reamplified with the same primer pair and used to transform thawed *S. pneumoniae* R6 cells in the presence of 10 ng of CSP-1 and 5 μl 500 mM calcium chloride. After 2 h incubation, cells were spread on blood agar plates supplemented with 5 mg l^−1^ erythromycin, and multiple colonies picked for screening using PCRs to identify mutants with different *spnIVRhsdS* genes, resulting in the isolation of the three patterns found in isolates *S. pneumoniae* R6 Aa, Ab and Ba.

### Construction of *S. pneumoniae* R6 *tvrR* knockouts

Three isolates with different putatively functional alleles of the *spnTVRhsdS* genes were identified from the collection^19^: CH2060, BR1109 and ND6010. For each of these, the region upstream of *tvrR* in the *tvr* locus was amplified through PCR using the primers LUpVL and LDwnVL, and the region downstream of the *tvr* locus amplified using RUpVL and RDwnVL. These primers added an *Apa*I site onto the 3′ end of the upstream product, and a *Bam*HI site onto the 5′ end of the downstream product. An *aph3′* gene was amplified from the Janus cassette^70^ using the primers kanL and kanR, which generated a DNA fragment containing the resistance marker flanked by *Apa*I and *Bam*HI sites. These three PCR products were then digested with the appropriate enzymes as described above, and ligated in equimolar proportions using T4 ligase (NEB) at room temperature for 10 min. Primers LUpVL and RDwnVL were then used to amplify the complete construct from the ligation reaction, which was purified using agarose gel electrophoresis. The extracted DNA was reamplified using primers LUpVL and RDwnVL, and then used to transform the parental isolate, using the appropriate CSP as determined from the genome sequence, and *S. pneumoniae* R6 Aa, using CSP-1, as described above.

### SMRT sequencing of samples

Initially 2–4 μg genomic DNA was converted into ~20 kb fragments through hydrodynamic shearing using a MegaRuptor (Diagenode). The DNA fragments were subsequently made into ‘SMRTbells’ by a process of damage repair, end-repair, adapter ligation and exonuclease-based removal of un-ligated molecules and adapters using DNA Template Prep Kit 2.0 (3–10 kb; Pacific Biosciences). The long-fragment SMRTbell libraries were subsequently annealed with primers and bound with P4 polymerase using the DNA/Polymerase Binding Kit P4. Sequencing was performed using the PacBio RSII by ‘MagBead loading’ of these complexes onto several V3 SMRTcells, which were each sequenced using 180 min movies.

Analysis of the data was conducted with using smrtanalysis version 2.1.0. *De novo* assembly, using PacBio data exclusively, was performed using the Hierarchical Genome Assembly Process, protocol RS_HGAP_Assembly.2. Base modification and motif analysis was performed using protocol RS_Modification_and_Motif_Analysis.1n. All data have been submitted to the ENA under the study accession code ERP005506.

## Author contributions

N.J.C., S.D.B. and W.P.H. conceived and designed the study. N.J.C., P.G.C., A.E.S. and A.C. performed the experiments. N.J.C. and W.P.H. wrote the manuscript, which was approved by all authors.

## Additional information

**How to cite this article:** Croucher, N. J. *et al*. Diversification of bacterial genome content through distinct mechanisms over different timescales. *Nat. Commun.* 5:5471 doi: 10.1038/ncomms6471 (2014).

**Accession codes:** The putative mobile genetic element and RMS sequences have been deposited in the European Nucleotide Archive under the accession codes LK020676 to LK020715. The SMRT sequence data have been deposited in the European Nucleotide Archive under the study code ERP005506.

## Supplementary Material

Supplementary Information Supplementary Figures 1-30, Supplementary Tables 1-8, Supplementary Methods and Supplementary References

## References

[b1] Langille M. G. I., Hsiao W. W. L. & Brinkman F. S. L. Detecting genomic islands using bioinformatics approaches. Nat. Rev. Microbiol. 8, 373–382 (2010). 20395967 10.1038/nrmicro2350

[b2] Johnston C., Martin B., Granadel C., Polard P. & Claverys J. P. Programmed protection of foreign DNA from restriction allows pathogenicity island exchange during pneumococcal transformation. PLoS Pathog. 9, e1003178 (2013). 23459610 10.1371/journal.ppat.1003178 PMC3573125

[b3] McDonnell M., Lain R. & Tomasz A. ‘Diplophage’: a bacteriophage of *Diplococcus pneumoniae*. Virology 63, 577–582 (1975). 234654 10.1016/0042-6822(75)90329-3

[b4] Romero P. . Comparative genomic analysis of ten *Streptococcus pneumoniae* temperate bacteriophages. J. Bacteriol. 191, 4854–4862 (2009). 19502408 10.1128/JB.01272-08 PMC2715734

[b5] Smith M. D. & Guild W. R. A plasmid in *Streptococcus pneumoniae*. J. Bacteriol. 137, 735–739 (1979). 33961 10.1128/jb.137.2.735-739.1979 PMC218350

[b6] Romero P. . Isolation and characterization of a new plasmid pSpnP1 from a multidrug-resistant clone of *Streptococcus pneumoniae*. Plasmid 58, 51–60 (2007). 17275906 10.1016/j.plasmid.2006.12.006

[b7] Shoemaker N. B., Smith M. D. & Guild W. R. Organization and transfer of heterologous chloramphenicol and tetracycline resistance genes in pneumococcus. J. Bacteriol. 139, 432–441 (1979). 37238 10.1128/jb.139.2.432-441.1979 PMC216887

[b8] Croucher N. J. . Role of conjugative elements in the evolution of the multidrug-resistant pandemic clone *Streptococcus pneumoniae*^Spain23F^ ST81. J. Bacteriol. 191, 1480–1489 (2009). 19114491 10.1128/JB.01343-08 PMC2648205

[b9] Samson J. E., Magadán A. H., Sabri M. & Moineau S. Revenge of the phages: defeating bacterial defences. Nat. Rev. Microbiol. 11, 675–687 (2013). 23979432 10.1038/nrmicro3096

[b10] Bikard D., Hatoum-Aslan A., Mucida D. & Marraffini L. A. CRISPR interference can prevent natural transformation and virulence acquisition during *in vivo* bacterial infection. Cell Host. Microbe. 12, 177–186 (2012). 22901538 10.1016/j.chom.2012.06.003

[b11] Lacks S. & Greenberg B. Complementary specificity of restriction endonucleases of *Diplococcus pneumoniae* with respect to DNA methylation. J. Mol. Biol. 114, 153–168 (1977). 20509 10.1016/0022-2836(77)90289-3

[b12] Morrison D. A. & Guild W. R. Transformation and deoxyribonucleic acid size: extent of degradation on entry varies with size of donor. J. Bacteriol. 112, 1157–1168 (1972). 4404818 10.1128/jb.112.3.1157-1168.1972 PMC251544

[b13] Lacks S. & Neuberger M. Membrane location of a deoxyribonuclease implicated in the genetic transformation of *Diplococcus pneumoniae*. J. Bacteriol. 124, 1321–1329 (1975). 366 10.1128/jb.124.3.1321-1329.1975 PMC236044

[b14] Cerritelli S., Springhorn S. S. & Lacks S. A. DpnA, a methylase for single-strand DNA in the *Dpn* II restriction system, and its biological function. Proc. Natl Acad. Sci. USA 86, 9223–9227 (1989). 2687877 10.1073/pnas.86.23.9223 PMC298466

[b15] Pozzi G. . Competence for genetic transformation in encapsulated strains of *Streptococcus pneumoniae*: two allelic variants of the peptide pheromone. J. Bacteriol. 178, 6087–6090 (1996). 8830714 10.1128/jb.178.20.6087-6090.1996 PMC178474

[b16] Whatmore A. M., Barcus V. A. & Dowson C. G. Genetic diversity of the streptococcal competence (*com*) gene locus. J. Bacteriol. 181, 3144–3154 (1999). 10322016 10.1128/jb.181.10.3144-3154.1999 PMC93770

[b17] Cornejo O. E., McGee L. & Rozen D. E. Polymorphic competence peptides do not restrict recombination in *Streptococcus pneumoniae*. Mol. Biol. Evol. 27, 694–702 (2010). 19942613 10.1093/molbev/msp287

[b18] Carrolo M., Pinto F. R., Melo-Cristino J. & Ramirez M. Pherotypes are driving genetic differentiation within *Streptococcus pneumoniae*. BMC Microbiol. 9, 191 (2009). 19735561 10.1186/1471-2180-9-191 PMC2751782

[b19] Croucher N. J. . Population genomics of post-vaccine changes in pneumococcal epidemiology. Nat. Genet. 45, 656–663 (2013). 23644493 10.1038/ng.2625 PMC3725542

[b20] Hanage W. P., Kaijalainen T., Saukkoriipi A., Rickcord J. L. & Spratt B. G. A successful, diverse disease-associated lineage of nontypeable pneumococci that has lost the capsular biosynthesis locus. J. Clin. Microbiol. 44, 743–749 (2006). 16517849 10.1128/JCM.44.3.743-749.2006 PMC1393106

[b21] Martin M. . An outbreak of conjunctivitis due to atypical *Streptococcus pneumoniae*. N. Engl. J. Med. 348, 1112–1121 (2003). 12646668 10.1056/NEJMoa022521

[b22] Tettelin H., Riley D., Cattuto C. & Medini D. Comparative genomics: the bacterial pan-genome. Curr. Opin. Microbiol. 11, 472–477 (2008). 19086349 10.1016/j.mib.2008.09.006

[b23] Donati C. . Structure and dynamics of the pan-genome of *Streptococcus pneumoniae* and closely related species. Genome Biol. 11, R107 (2010). 21034474 10.1186/gb-2010-11-10-r107 PMC3218663

[b24] Fraser C., Hanage W. P. & Spratt B. G. Recombination and the nature of bacterial speciation. Science 315, 476–480 (2007). 17255503 10.1126/science.1127573 PMC2220085

[b25] Brown J. S., Gilliland S. M., Spratt B. G. & Holden D. W. A locus contained within a variable region of pneumococcal pathogenicity island 1 contributes to virulence in mice. Infect. Immun. 72, 1587–1593 (2004). 14977965 10.1128/IAI.72.3.1587-1593.2004 PMC356060

[b26] Harvey R. M. . A variable region within the genome of *Streptococcus pneumoniae* contributes to strain-strain variation in virulence. PLoS ONE 6, e19650 (2011). 21573186 10.1371/journal.pone.0019650 PMC3088708

[b27] Croucher N. J. . Dominant role of nucleotide substitution in the diversification of serotype 3 pneumococci over decades and during a single infection. PLoS. Genet. 9, e1003868 (2013). 24130509 10.1371/journal.pgen.1003868 PMC3794909

[b28] Wyres K. L. . Evidence of antimicrobial resistance-conferring genetic elements among pneumococci isolated prior to 1974. BMC Genomics 14, 500 (2013). 23879707 10.1186/1471-2164-14-500 PMC3726389

[b29] Croucher N. J., Vernikos G. S., Parkhill J. & Bentley S. D. Identification, variation and transcription of pneumococcal repeat sequences. BMC Genomics 12, 120 (2011). 21333003 10.1186/1471-2164-12-120 PMC3049150

[b30] Shahinas D. . Comparative genomic analyses of *Streptococcus pseudopneumoniae* provide insight into virulence and commensalism dynamics. PLoS ONE 8, e65670 (2013). 23840352 10.1371/journal.pone.0065670 PMC3686770

[b31] Denapaite D. . The genome of *Streptococcus mitis* B6-what is a commensal? PLoS ONE 5, e9426 (2010). 20195536 10.1371/journal.pone.0009426 PMC2828477

[b32] Bobay L.-M., Rocha E. P. C. & Touchon M. The adaptation of temperate bacteriophages to their host genomes. Mol. Biol. Evol. 30, 737–751 (2013). 23243039 10.1093/molbev/mss279 PMC3603311

[b33] Camilli R. . Complete genome sequence of a serotype 11A, ST62 *Streptococcus pneumoniae* invasive isolate. BMC Microbiol. 11, 25 (2011). 21284853 10.1186/1471-2180-11-25 PMC3055811

[b34] Mountford M. D. in *Progress in Soil Zoology* (ed. Murphy, P.W.) 43–50 (Butterworths, 1962).

[b35] Brochet M. . Shaping a bacterial genome by large chromosomal replacements, the evolutionary history of *Streptococcus agalactiae*. Proc. Natl Acad. Sci. USA 105, 15961–15966 (2008). 18832470 10.1073/pnas.0803654105 PMC2572952

[b36] Burrus V., Pavlovic G., Decaris B. & Guedon G. The ICE*St*1 element of *Streptococcus thermophilus* belongs to a large family of integrative and conjugative elements that exchange modules and change their specificity of integration. Plasmid 48, 77–97 (2002). 12383726 10.1016/s0147-619x(02)00102-6

[b37] Croucher N. J. . Rapid pneumococcal evolution in response to clinical interventions. Science 331, 430–434 (2011). 21273480 10.1126/science.1198545 PMC3648787

[b38] Palmieri C. . Characterization of a *Streptococcus suis tet*(O/W/32/O)-carrying element transferable to major streptococcal pathogens. Antimicrob. Agents Chemother. 56, 4697–4702 (2012). 22710115 10.1128/AAC.00629-12 PMC3421841

[b39] Croucher N. J. . Evidence for soft selective sweeps in the evolution of pneumococcal multidrug resistance and vaccine escape. Genome Biol. Evol. 6, 1589–1602 (2014). 24916661 10.1093/gbe/evu120 PMC4122920

[b40] Croucher N. J. . Variable recombination dynamics during the emergence, transmission and `disarming' of a multidrug-resistant pneumococcal clone. BMC Biol. 12, 49 (2014). 24957517 10.1186/1741-7007-12-49 PMC4094930

[b41] Wozniak R. A. F. & Waldor M. K. Integrative and conjugative elements: mosaic mobile genetic elements enabling dynamic lateral gene flow. Nat. Rev. Microbiol. 8, 552–563 (2010). 20601965 10.1038/nrmicro2382

[b42] Novick R. P., Christie G. E. & Penadés J. R. The phage-related chromosomal islands of Gram-positive bacteria. Nat. Rev. Microbiol. 8, 541–551 (2010). 20634809 10.1038/nrmicro2393 PMC3522866

[b43] Lindsay J. A., Ruzin A., Ross H. F., Kurepina N. & Novick R. P. The gene for toxic shock toxin is carried by a family of mobile pathogenicity islands in *Staphylococcus aureus*. Mol. Microbiol. 29, 527–543 (1998). 9720870 10.1046/j.1365-2958.1998.00947.x

[b44] Matos R. C. . *Enterococcus faecalis* prophage dynamics and contributions to pathogenic traits. PLoS Genet. 9, e1003539 (2013). 23754962 10.1371/journal.pgen.1003539 PMC3675006

[b45] Rankin D. J., Rocha E. P. C. & Brown S. P. What traits are carried on mobile genetic elements, and why? Heredity (Edinb) 106, 1–10 (2010). 20332804 10.1038/hdy.2010.24 PMC3183850

[b46] Punta M. . The Pfam protein families database. Nucleic. Acids Res. 40, D290–D301 (2012). 22127870 10.1093/nar/gkr1065 PMC3245129

[b47] Croucher N. J., Harris S. R., Barquist L., Parkhill J. & Bentley S. D. A high-resolution view of genome-wide pneumococcal transformation. PLoS. Pathog. 8, e1002745 (2012). 22719250 10.1371/journal.ppat.1002745 PMC3375284

[b48] Tettelin H. . Complete genome sequence of a virulent isolate of *Streptococcus pneumoniae*. Science 293, 498–506 (2001). 11463916 10.1126/science.1061217

[b49] Hoskins J. . Genome of the bacterium *Streptococcus pneumoniae* strain R6. J. Bacteriol. 183, 5709–5717 (2001). 11544234 10.1128/JB.183.19.5709-5717.2001 PMC95463

[b50] Roberts R. J., Vincze T., Posfai J. & Macelis D. REBASE-A database for DNA restriction and modification: Enzymes, genes and genomes. Nucleic Acids Res. 38, D234–D236 (2009). 19846593 10.1093/nar/gkp874 PMC2808884

[b51] Rocha E. P. Evolutionary patterns in prokaryotic genomes. Curr. Opin. Microbiol. 11, 454–460 (2008). 18838127 10.1016/j.mib.2008.09.007

[b52] Naito T., Kusano K. & Kobayashi I. Selfish behavior of restriction-modification systems. Science 267, 897–899 (1995). 7846533 10.1126/science.7846533

[b53] Rocha E. P. C. Order and disorder in bacterial genomes. Curr. Opin. Microbiol 7, 519–527 (2004). 15451508 10.1016/j.mib.2004.08.006

[b54] Dybvig K., Sitaraman R. & French C. T. A family of phase-variable restriction enzymes with differing specificities generated by high-frequency gene rearrangements. Proc. Natl Acad. Sci. USA 95, 13923–13928 (1998). 9811902 10.1073/pnas.95.23.13923 PMC24968

[b55] Cerdeño-Tárraga A. M. . Extensive DNA inversions in the *B. fragilis* genome control variable gene expression. Science 307, 1463–1465 (2005). 15746427 10.1126/science.1107008

[b56] Seib K. L., Peak I. R. A. & Jennings M. P. Phase variable restriction-modification systems in *Moraxella catarrhalis*. FEMS Immunol. Med. Microbiol. 32, 159–165 (2002). 11821238 10.1111/j.1574-695X.2002.tb00548.x

[b57] De Vries N. . Transcriptional phase variation of a type III restriction-modification system in *Helicobacter pylori*. J. Bacteriol. 184, 6615–6623 (2002). 12426350 10.1128/JB.184.23.6615-6623.2002 PMC135423

[b58] McMahon S. A. . Extensive DNA mimicry by the ArdA anti-restriction protein and its role in the spread of antibiotic resistance. Nucleic Acids Res. 37, 4887–4897 (2009). 19506028 10.1093/nar/gkp478 PMC2731889

[b59] R Core Development Team. R: A language and environment for statistical computing R Foundation for Statistical Computing (2011).

[b60] Dixon P. VEGAN, a package of R functions for community ecology. J. Veg. Sci. 14, 927–930 (2003).

[b61] Paradis E., Claude J. & Strimmer K. APE: analyses of phylogenetics and evolution in R language. Bioinformatics 20, 289–290 (2004). 14734327 10.1093/bioinformatics/btg412

[b62] Eddy S. R. Accelerated profile HMM searches. PLoS Comput. Biol. 7, e1002195 (2011). 22039361 10.1371/journal.pcbi.1002195 PMC3197634

[b63] Kent W. J. BLAT-the BLAST-like alignment tool. Genome Res. 12, 656–664 (2002). 11932250 10.1101/gr.229202 PMC187518

[b64] Carver T. . Artemis and ACT: viewing, annotating and comparing sequences stored in a relational database. Bioinformatics 24, 2672–2676 (2008). 18845581 10.1093/bioinformatics/btn529 PMC2606163

[b65] Li H. & Durbin R. Fast and accurate short read alignment with Burrows–Wheeler transform. Bioinformatics 25, 1754–1760 (2009). 19451168 10.1093/bioinformatics/btp324 PMC2705234

[b66] Cock P. J. A. . Biopython: freely available Python tools for computational molecular biology and bioinformatics. Bioinformatics 25, 1422–1423 (2009). 19304878 10.1093/bioinformatics/btp163 PMC2682512

[b67] Siguier P., Perochon J., Lestrade L., Mahillon J. & Chandler M. ISfinder: the reference centre for bacterial insertion sequences. Nucleic Acids Res. 34, D32–D36 (2006). 16381877 10.1093/nar/gkj014 PMC1347377

[b68] Edgar R. C. MUSCLE: multiple sequence alignment with high accuracy and high throughput. Nucleic Acids Res. 32, 1792–1797 (2004). 15034147 10.1093/nar/gkh340 PMC390337

[b69] Tang J., Hanage W. P., Fraser C. & Corander J. Identifying currents in the gene pool for bacterial populations using an integrative approach. PLoS. Comput. Biol. 5, e1000455 (2009). 19662158 10.1371/journal.pcbi.1000455 PMC2713424

[b70] Sung C. K., Li H., Claverys J. P. & Morrison D. A. An *rpsL* cassette, Janus, for gene replacement through negative selection in *Streptococcus pneumoniae*. Appl. Environ. Microbiol. 67, 5190–5196 (2001). 11679344 10.1128/AEM.67.11.5190-5196.2001 PMC93289

