# Supplementary material for: Diversification of bacterial genome content through distinct mechanisms over different timescales
Source: Nat Commun. 2014 Nov 19;5:5471. doi: 10.1038/ncomms6471 (PMC4263131; doi:10.1038/ncomms6471)
Supplement: Supplementary Information — Supplementary Figures 1-30, Supplementary Tables 1-8, Supplementary Methods and Supplementary References [file ncomms6471-s1.pdf]

Supplementary Figures

Supplementary Figure 1

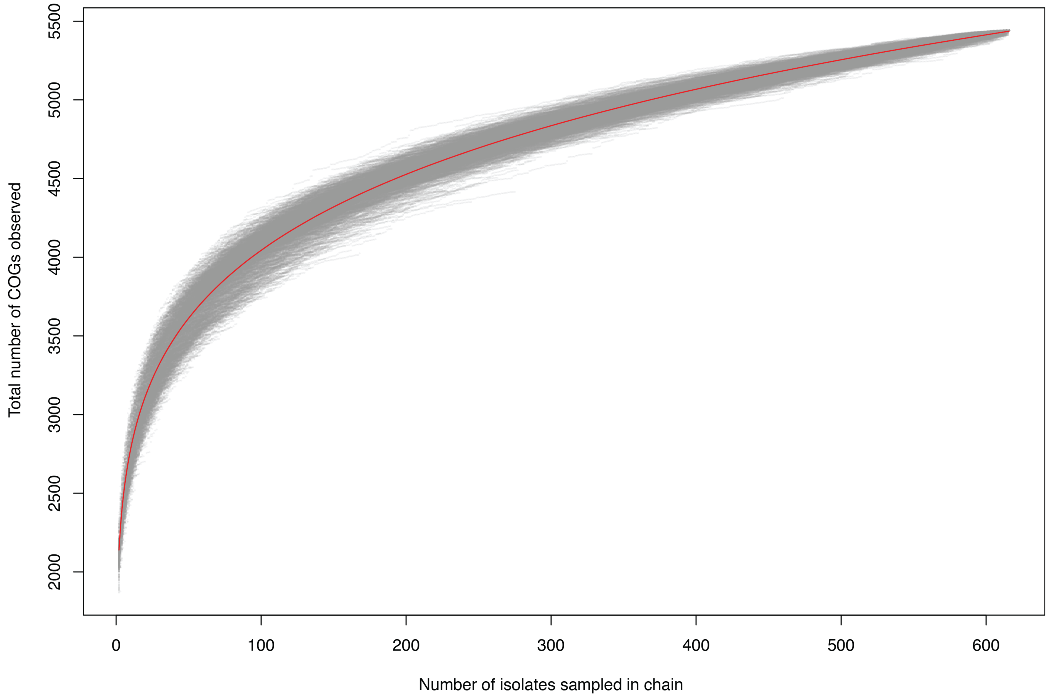

Supplementary Figure 1: Pangenome of the pneumococcal population. One thousand replicates were performed in which every isolate was sequentially sampled in a chain, without replacement. For each point in each chain, the total number of COGs observed up to that point was plotted against the number of isolates sampled. A power law function of the form $y = \kappa x^{\gamma}$ was fitted to these data, represented by the red curve. This estimated κ as 1,910 (95% confidence interval of 1,910-1,911) and γ as 0.1628 (95% confidence interval of 0.1628-0.1629). Values of γ greater than zero indicate an 'open' pangenome.

Supplementary Figure 2

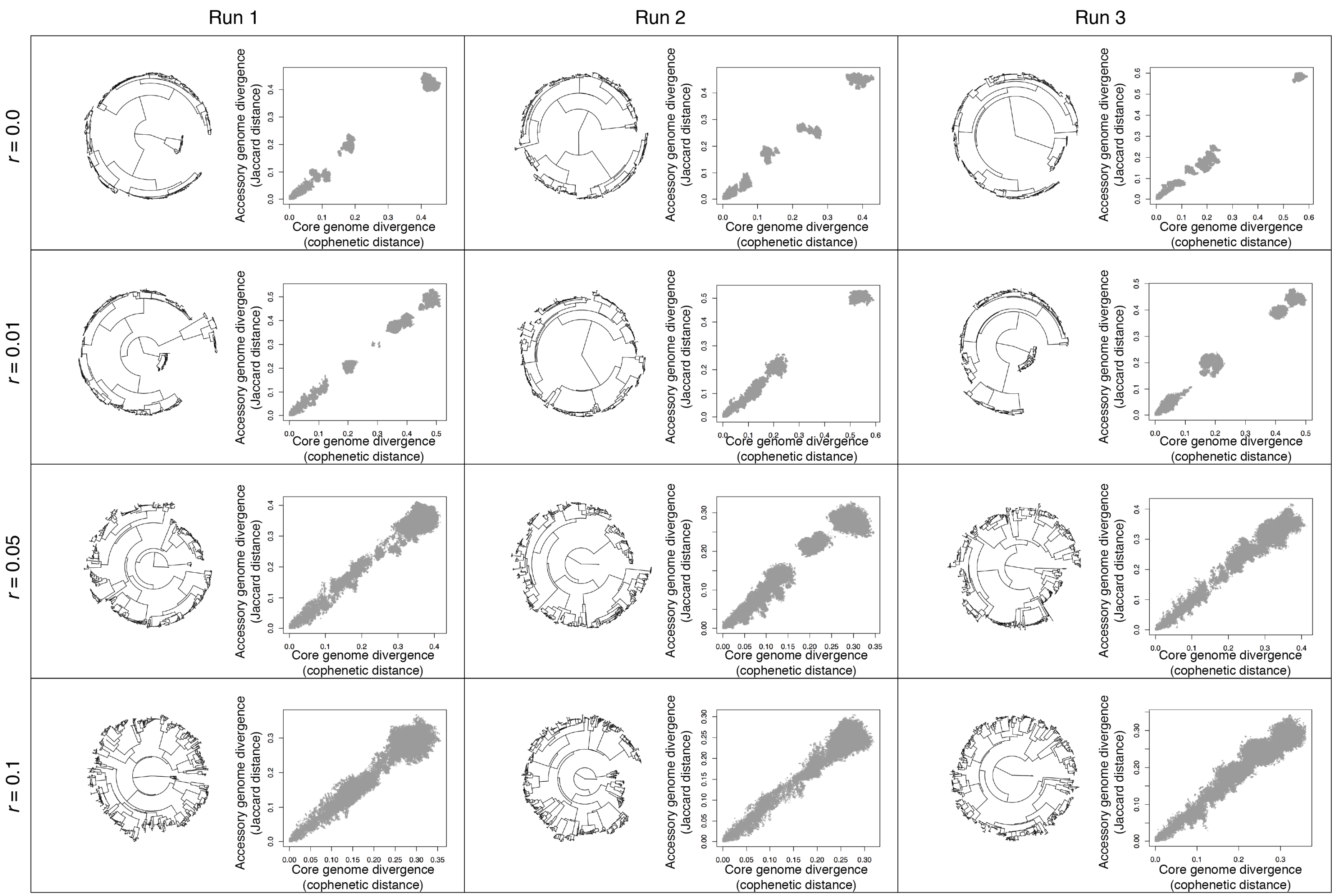

Supplementary Figure 2: Simulations of the co-existence of evolving lineages in a single population (see Methods). Populations of 1,000 isolates were initialized with fifteen distinct randomly-generated lineages, the members of each initially being identical, which could exchange sequence through recombination. Subsequent neutral evolution was simulated with Wright-Fisher models, in which isolates mutated at equal rates in both the core and accessory genome, and exchanged the same proportion of each through recombination. The rate of recombination relative to mutation (quantified by *r*) was set at different values on each row; three examples are shown for each value, with each simulation run for 10,000 generations. Using the 1,000 sequences from the final generation, a neighbor-joining tree was calculated and used to generate a set of cophenetic distances; these were plotted against the Jaccard distances between simulated sequences' accessory genomes as for the real dataset in Figure 1. The lower the value of *r*, the more discontinuous the distribution of pairwise distances, as there was a substantial clonal element to the different lineages' evolution. These have a stronger similarity to the actual data than the results from the simulations in which recombination was more frequent, in which pairwise distances were distributed more homogeneously along the identity line as recombination tended to inhibit the co-existence of distinct lineages.

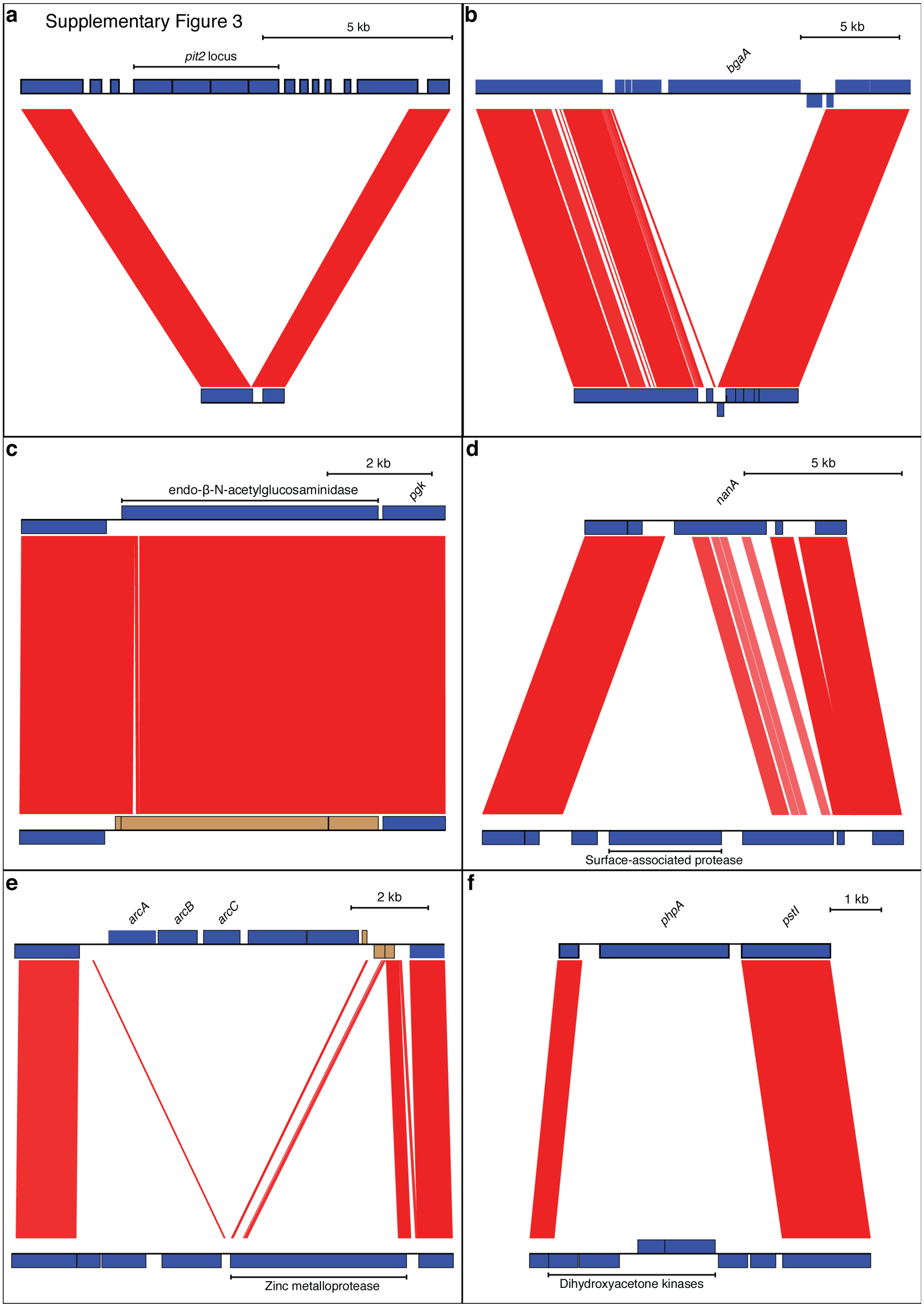

a
Supplementary Figure 3
5 kb
pit2 locus
b
5 kb
bgaA
c
2 kb
endo-β-N-acetylglucosaminidase
pgk
d
5 kb
nanA
Surface-associated protease
e
arcA
arcB
arcC
2 kb
Zinc metalloprotease
f
phpA
pstI
1 kb
Dihydroxyacetone kinases

Supplementary Figure 3: Differences between SC12 and the rest of the pneumococcal population. The panels show comparisons between the genome of *S. pneumoniae* ATCC 700669 at the top, and a *de novo* assembly of an SC12 isolate on the bottom. Blue boxes indicate functional coding sequences (CDSs), with their vertical position indicating whether they are encoded on the forward or reverse strand of the genome; brown boxes represent pseudogenes. Red bands indicate regions of sequence similarity, as detected by BLAT, with the intensity of the colour representing the level of similarity. Displayed are the (a) *pit2* locus within PPI-1; (b) *bgaA* locus; (c) frameshift within the surface-displayed acetylglucosaminidase; (d) *nanA* locus; (e) replacement of the *arc* operon with a zinc metalloprotease; and (f) replacement of *phpA* with a metabolic operon.

Supplementary Figure 4

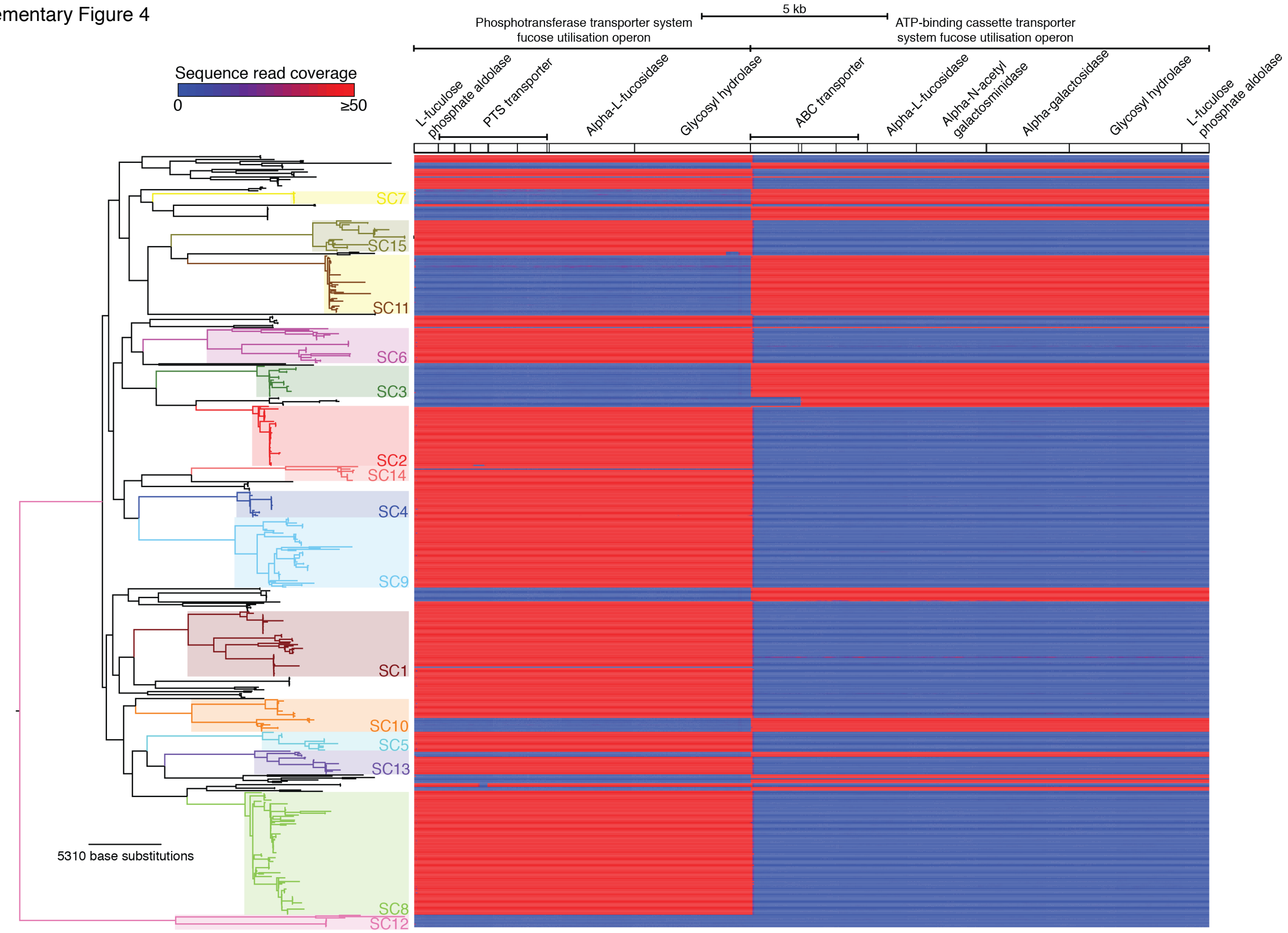

Supplementary Figure 4: Distribution of fucose utilisation genes. The core genome phylogeny is displayed adjacent to a heatmap showing mapping to two fucose utilisation gene clusters, one of which relies on a PTS transporter to import the sugar, and the other on an ABC transporter. Each row of the heatmap relates to a single leaf node of the phylogeny; blue regions indicate an absence of read mapping, while red regions indicate read mapping coverage up to a maximum of 50-fold, demonstrating the locus is present in the relevant isolate. Both operons show strong conservation within sequence clusters, with SC12 the only genotype to lack either gene cluster.

Supplementary Figure 5

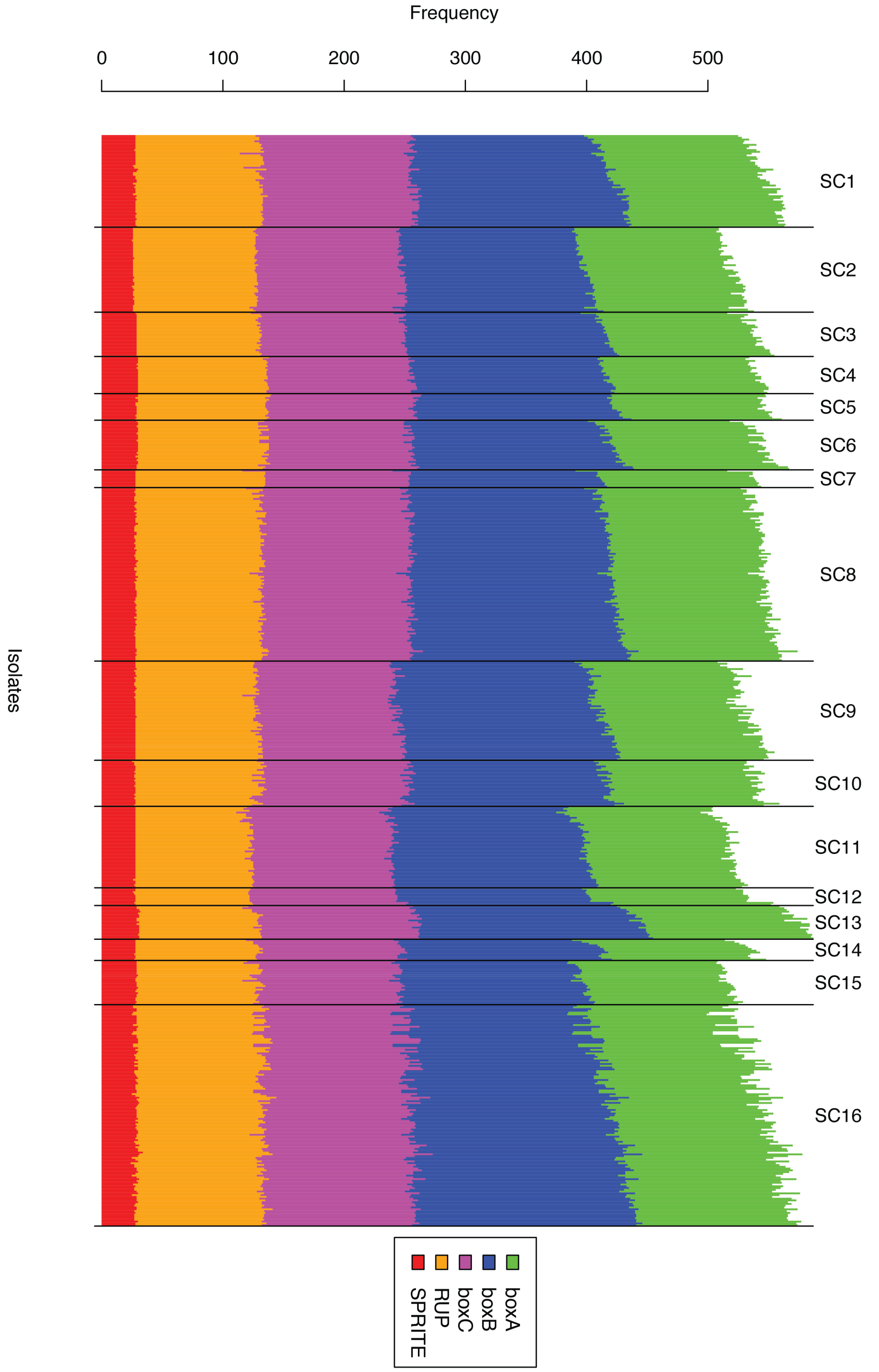

Supplementary Figure 5: Distribution of small interspersed repeat sequences. The *de novo* assembly of each sequence was scanned for small interspersed repeats and the frequencies plotted as stacked bar charts. Isolates are ordered by sequence cluster and the number of boxB repeats they contain. There was little variation in SPRITE elements across the species; RUP, boxA and boxC showed some evidence of between-sequence cluster variation. The IS elements to which the mobility of RUP and BOX elements have been ascribed, IS630-*Spn*1 and IS*Spn*2 respectively, are both ubiquitous across the collection (Supplementary Figure 6), which could explain the observed variation. By contrast, boxB exhibited a much higher level of within-sequence cluster variation. BOX elements are typically composed of single boxA and boxC sequences at their 5' and 3' ends, respectively, with a variable number of boxB sequences in tandem between them. Hence the variation in boxB content, without a corresponding alteration in the number of boxA and boxC sequences, was likely due to the expansion and contraction of tandem repeat arrays rather than changes in the frequency of BOX elements.

Supplementary Figure 6

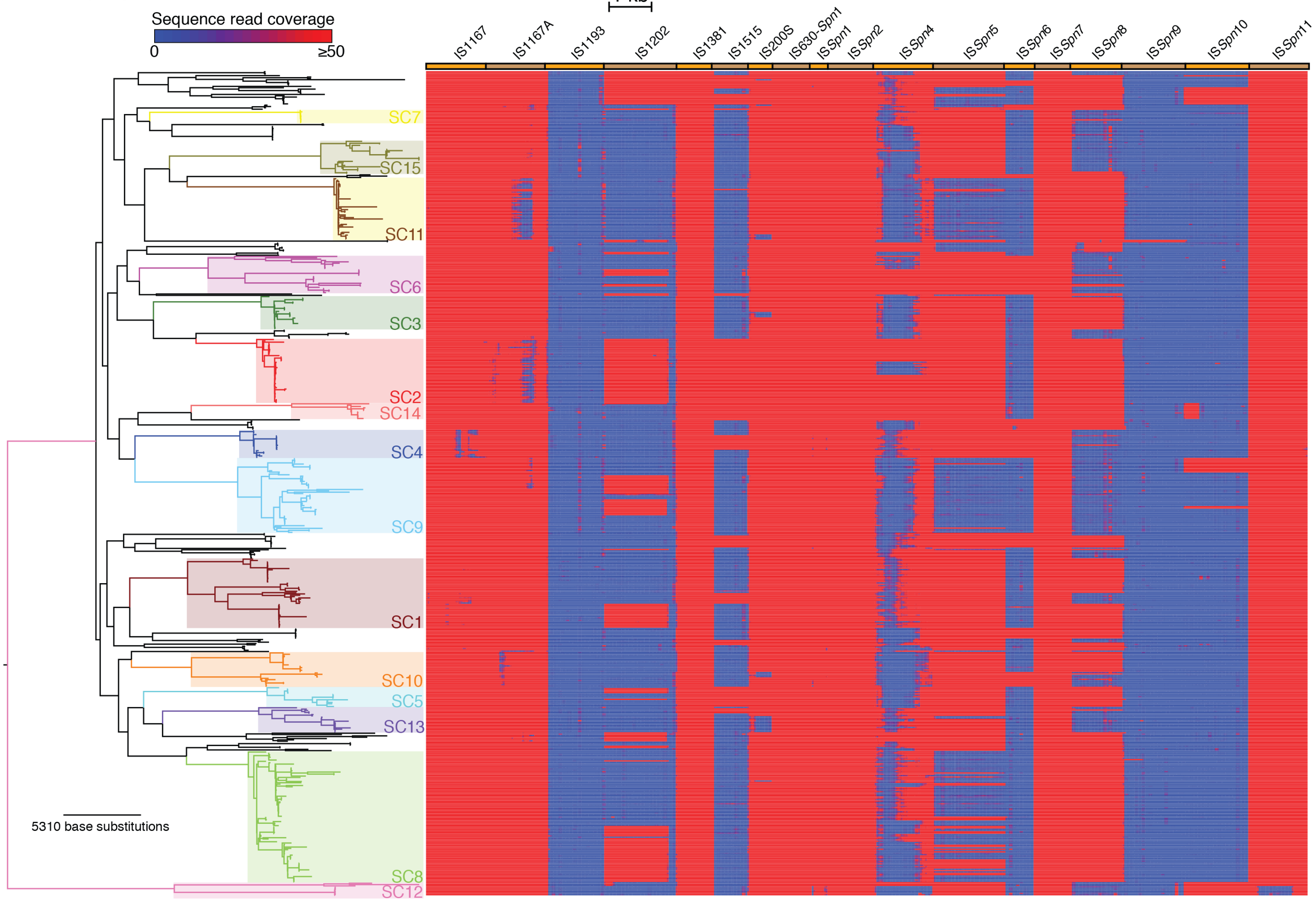

Supplementary Figure 6: Distribution of insertion sequences. This heatmap shows the sequence read mapping to each of the insertion sequences found in the pneumococcus (according to the ISFinder database), which are indicated across the top of the figure by alternating orange and brown bars. Each row of the heatmap relates to a single leaf node of the phylogeny; blue regions indicate an absence of read mapping, while red regions indicate read mapping coverage up to a maximum of 50-fold, demonstrating the IS is present in the relevant isolate. Partial mapping to IS*Spn4* is observed when it is absent from a genome but IS*Spn*11 is present, as these two ISs share similar termini.

Supplementary Figure 7

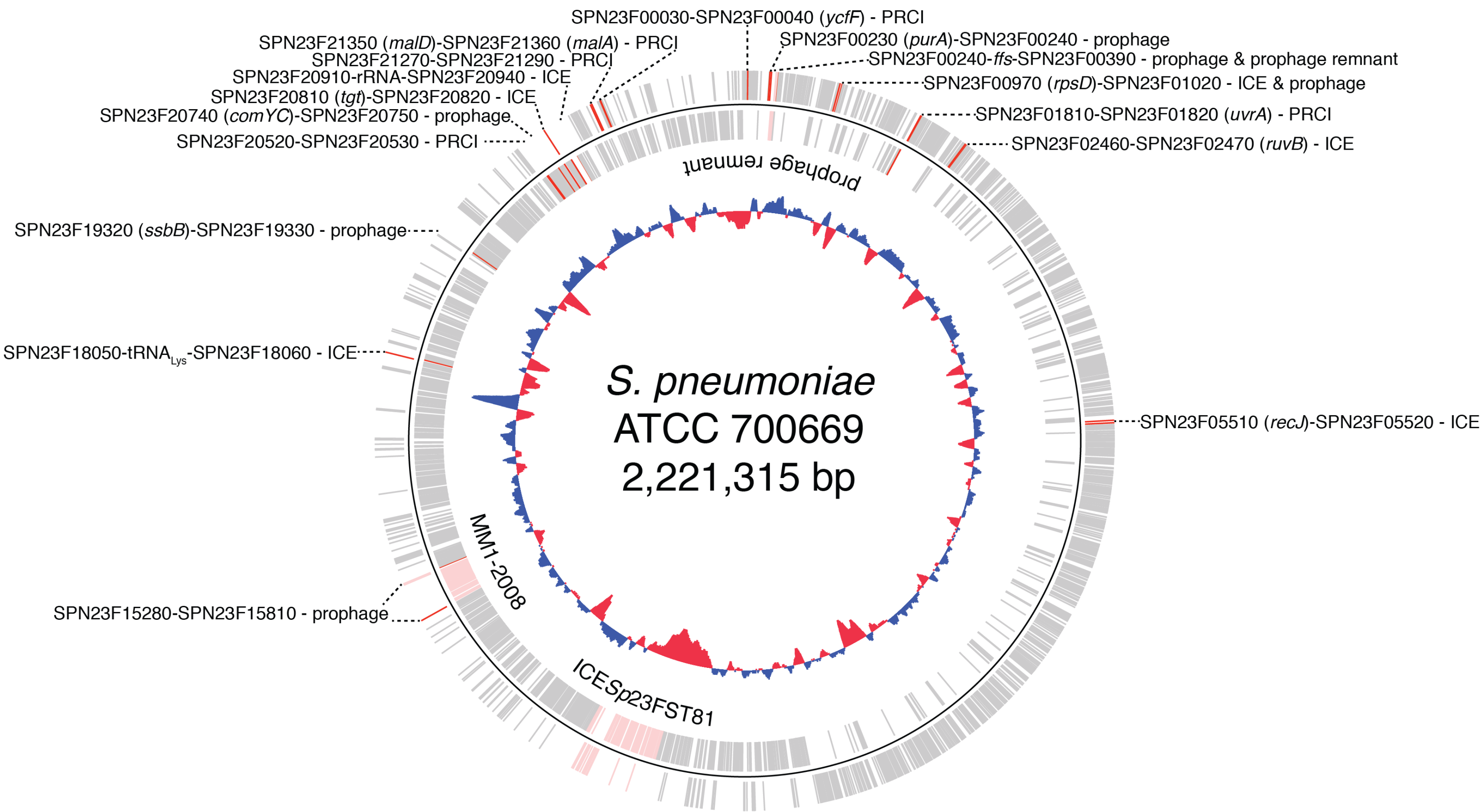

Supplementary Figure 7: Insertion sites of MGEs. Sixteen insertion sites were identified in the scan for multi-gene MGEs, each of which is labelled relative to the complete genome of *S. pneumoniae* ATCC 700669. The GC content of the genome is plotted as the red and blue graph on the inner track. Pink boxes represent MGE CDSs: these are found on ICE*Sp*23FST81, prophage ϕMM1-2008 and the prophage remnant. Red CDSs represent the core CDSs that comprise the sixteen identified insertion sites, which are labelled with the type of MGE found at that location. In the three cases where a non-coding RNA was found between the CDSs defining the insertion site, this is also labelled. Of the six insertion sites labelled as containing putative prophage, the orientation of insertion could only be established for five. The remaining site between SPN23F00970 and SPN23F01020 was occupied by putative MGEs in some representatives of SC13 and SC16; these had an atypical genetic structure that made them difficult to classify or annotate (see Supplementary Methods). The insertion sites for Tn*5253*-type ICEs were not identified in this analysis owing to the considerable sequence diversity present at all three sites even in isolates lacking intact ICEs. One was adjacent to the variable *zmpA* gene, encoding the immunoglobulin A protease, as observed for Tn*5253*; a second was within PPI-1, likely representing the type of event that originally gave rise to this GI, and the third was near *rplL*, a site at which the many ICE 'scars' that comprised a substantial proportion of component A were evident, indicating a long history of being targeted by such MGEs.

Supplementary Figure 8

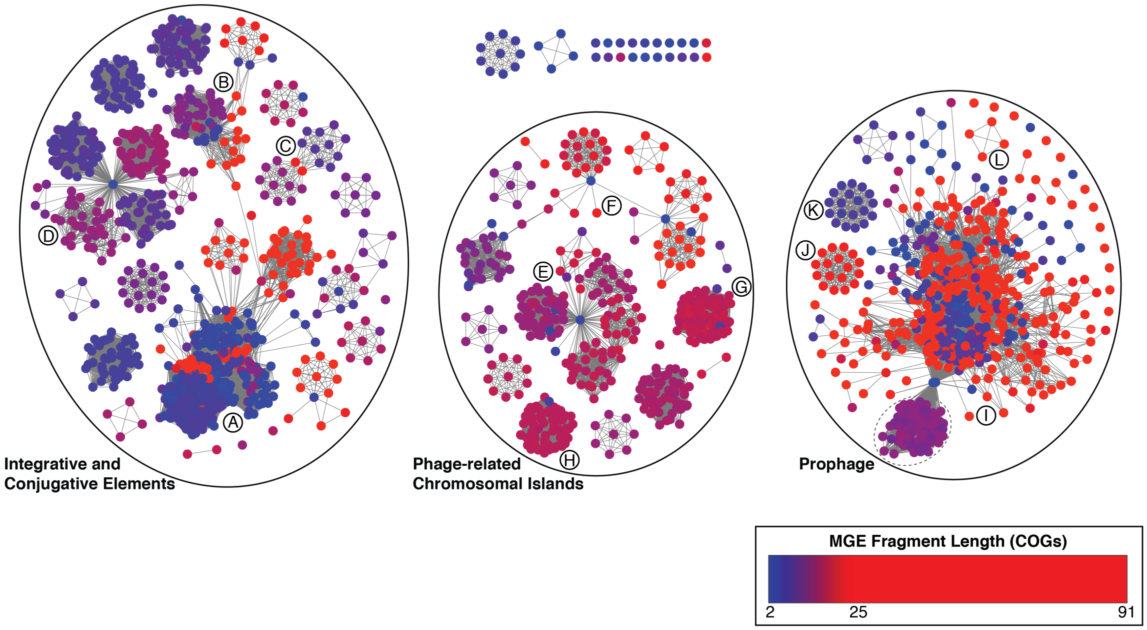

Supplementary Figure 8: Lengths of MGEs. The nodes of the network displayed in Figure 3 were recoloured according to the length of the putative MGEs. Blue nodes are shorter putative MGEs, whereas red MGEs are longer, as indicated by the key.

Supplementary Figure 9

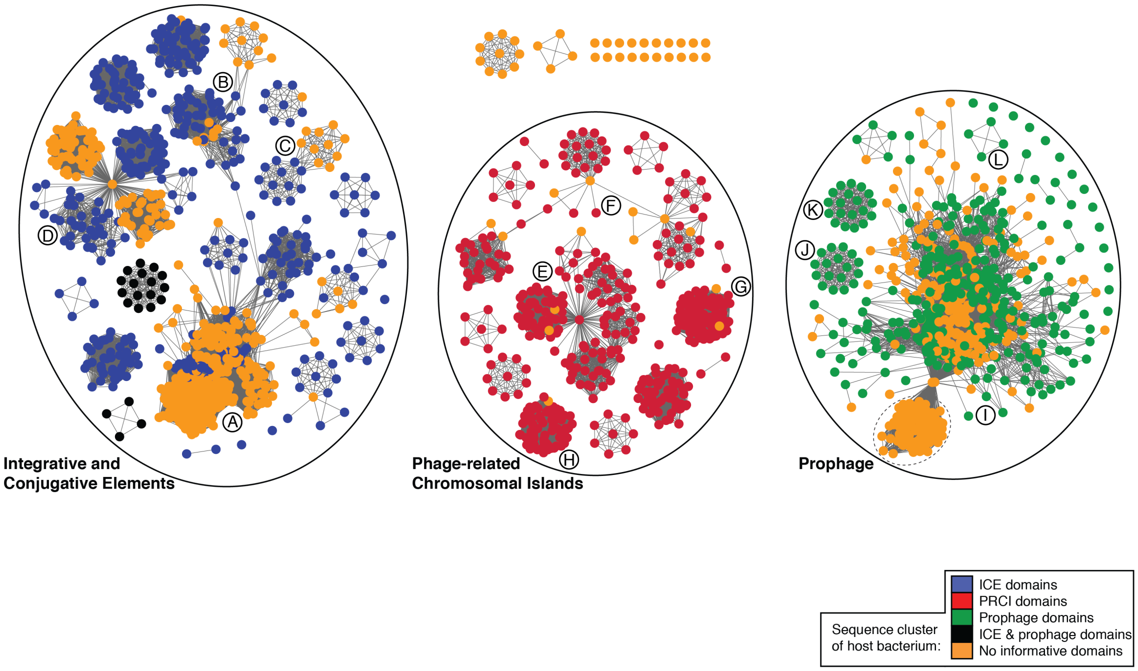

Supplementary Figure 9: Distribution of functional domains. The nodes of the network of putative MGEs displayed in Figure 3 were recoloured according to the presence of informative functional domains. Any putative MGE including a COG associated with a domain indicating a role in DNA translocation (FtsK_SpoIIIE, SpoIIIAH, MobC, TrbL or T4SS-DNA_transf) was coloured blue. Any putative MGE including a COG associated with a structural or DNA packaging role typical of prophage (Terminase_1, Terminase_2, Terminase_3, Terminase_4, Terminase_5, Terminase_6, Phage_tail, Sipho_tail, PhageMin_Tail, Phage_H_T_join, Prophage_tail) was coloured green. Any putative MGE including a COG associated with a functional domain that was consistently found in PRCI-type sequences (XhlA, Phage_pRha, Phage_Nu1) was coloured red. The black network components represent putative MGEs that had characteristics of ICEs but included COG CLS02376, which had a weak hit to the Phage_Nu1 domain typically associated with PRCIs. See Supplementary Methods for details.

Supplementary Figure 10

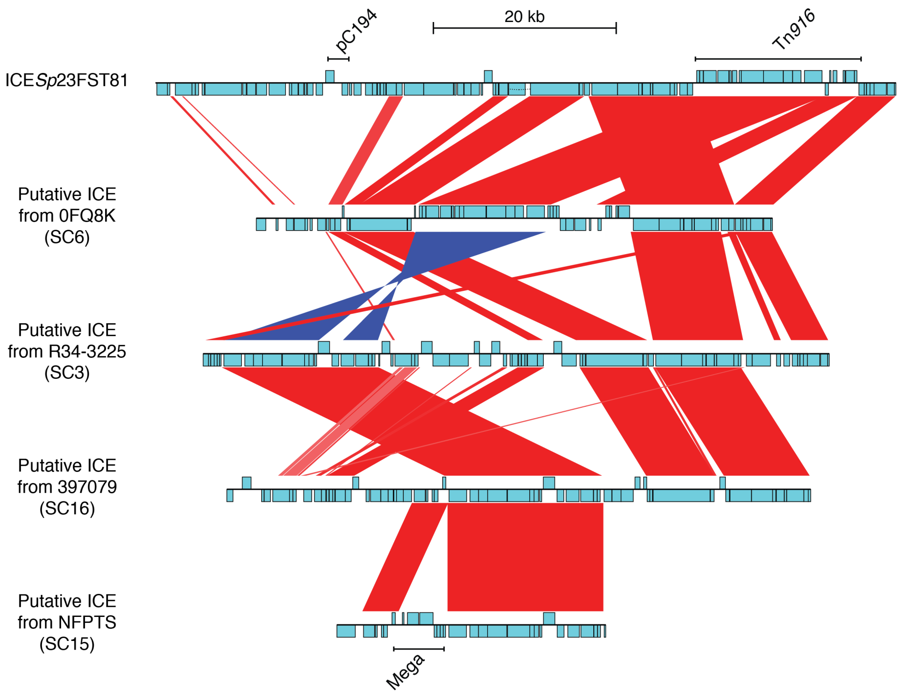

Supplementary Figure 10: Tn*5253* and Tn*916*-type ICEs found in the set of nodes labelled A. The sequence at the top is ICE*Sp*23FST81, the Tn*5253*-type ICE found in the multidrug-resistant isolate *S. pneumoniae* ATCC 700669 [EMBL accession: FM211187]. The linearized chloramphenicol resistance plasmid pC194 is annotated, as is the Tn*916*-type component inserted into the Tn*5252*-type backbone. Beneath are three Tn*5253*-type ICEs found in antibiotic resistant isolates of SC6, SC3 and SC16, and at the bottom is a Tn*916*-type ICE (including a mega macrolide resistance cassette) found in the multidrug-resistant PMEN14 lineage, corresponding to SC15 in this population. These illustrate the modular variation characteristic of ICEs. In this alignment, red bands between sequences represent regions of sequence similarity in the same orientation, as identified from comparisons of translated nucleotide sequences using BLAT. Twisted blue bands linking sequences indicate regions of similarity identified by such BLAT comparisons in the inverse orientation. In both cases, the intensity of the colour represents the strength of the match.

Supplementary Figure 11

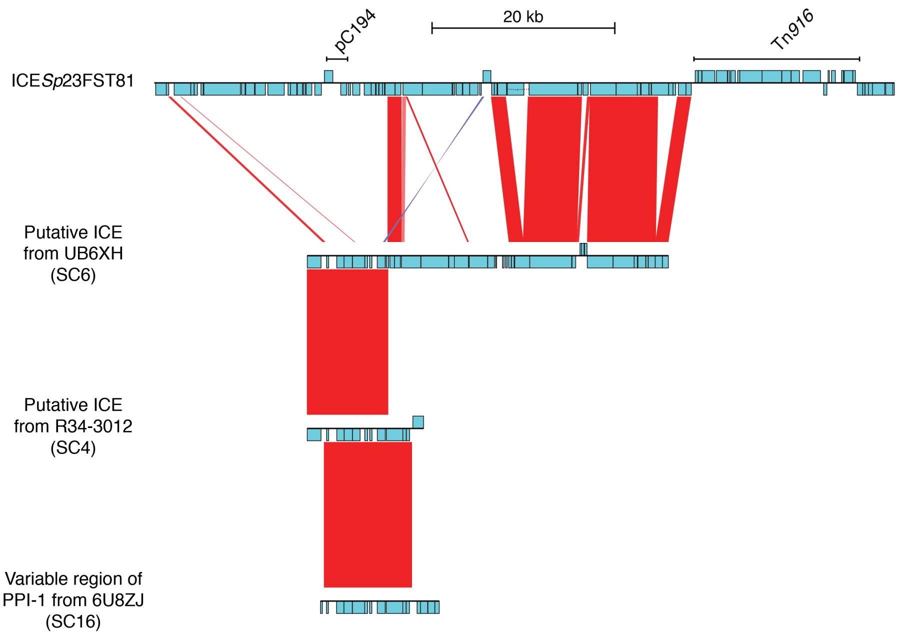

Supplementary Figure 11: ICEs found in the set of nodes labelled B, displayed as described in Supplementary Figure 10. ICE*Sp*23FST81 is again displayed at the top. The putative Tn*5252*-type ICE from SC6 lacks any of the resistance genes found in ICE*Sp*23FST81, and is similar to ICE*Sp*PN1[1]. The 5' region of this ICE matches a smaller element, lacking the Tn*5252* transfer machinery, found in SC4, which itself closely matches the PPI-1 variable region found in some isolates in this collection and *S. pneumoniae* TIGR4. This latter sequence was not identified as an MGE in this analysis. These sequences again illustrate the modular evolution characteristic of ICEs.

Supplementary Figure 12

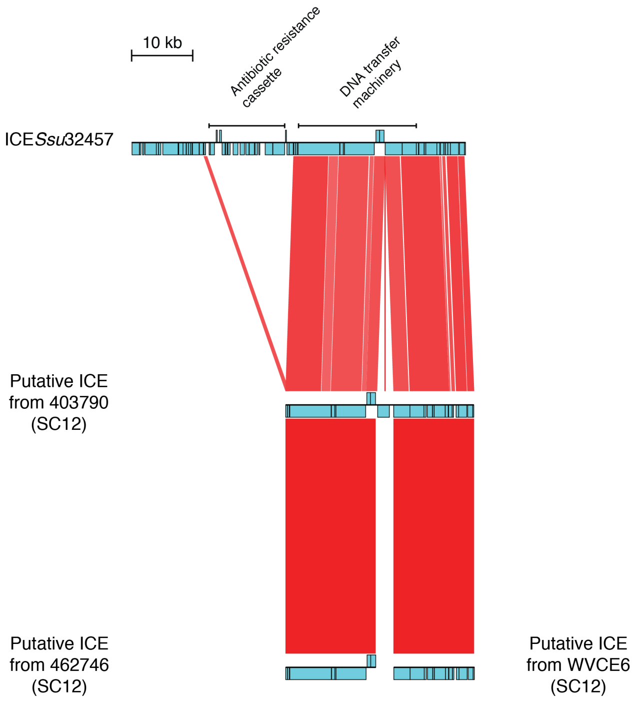

Supplementary Figure 12: ICEs found in component C, displayed as described in Supplementary Figure 10. At the top is displayed ICE*Ssu*32457 [EMBL accession: FR823304] from *S. suis*. This larger element includes a cassette encoding multiple antibiotic resistance genes; however, this is absent from the ICEs in SC12. The putative ICE from 403790 appears to represent the complete form of this element; in isolates 462746 and WVCE6, the element assembled in two fragments.

Supplementary Figure 13

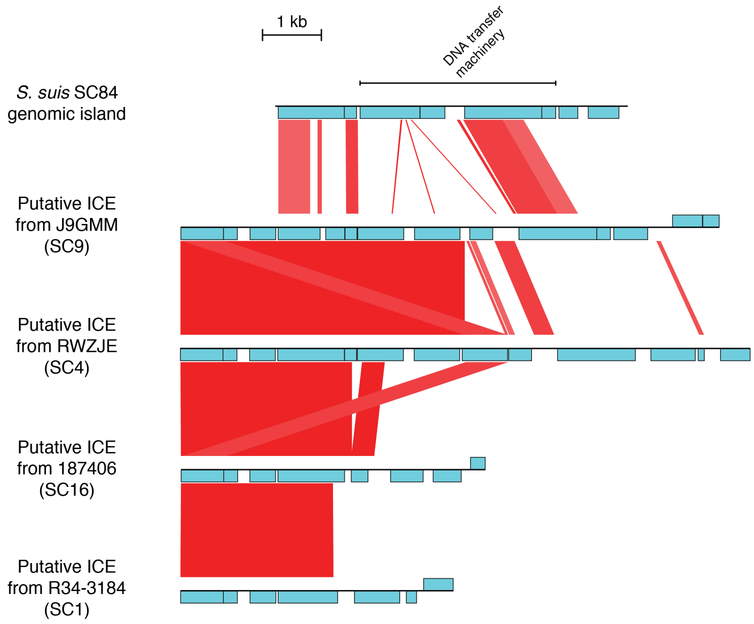

Supplementary Figure 13: ICEs found in component D, displayed as described in Supplementary Figure 10. At the top is displayed a genomic island (SSUSC84_0097-SSUSC84_0104) from *S. suis* SC84 [EMBL accession: FM252031]. Beneath are aligned four similar ICEs, which share a common 5' region that encodes an integrase.

Supplementary Figure 14

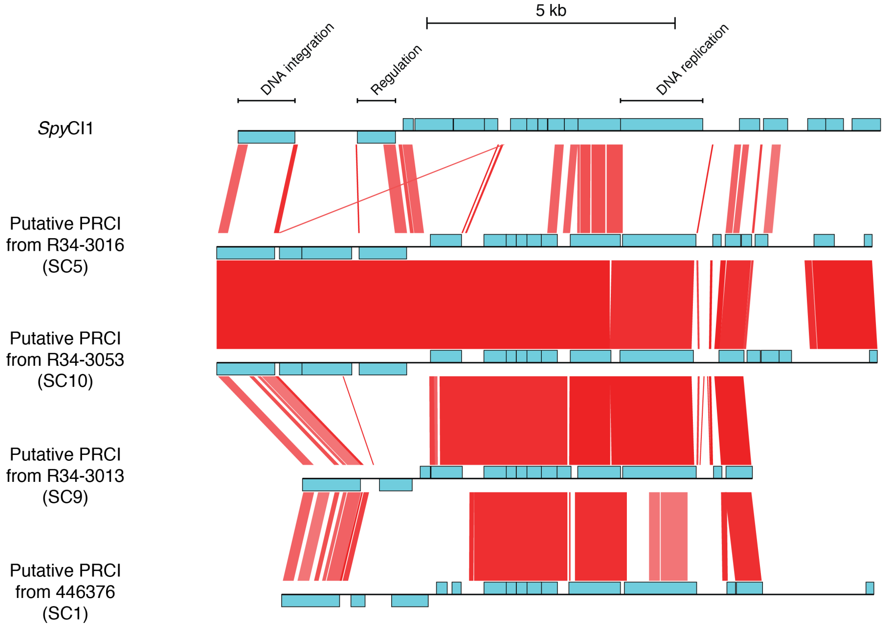

Supplementary Figure 14: Putative PRCIs found in component E, displayed as described in Supplementary Figure 10. At the top is displayed the PRCI *Spy*CI1 (SPy_2122-SPy_2147) from *Streptococcus pyogenes* SF370 [EMBL accession: AE004092]. Beneath are displayed putative PRCIs from four different sequence clusters that exhibit a mosaic pattern of similarity.

Supplementary Figure 15

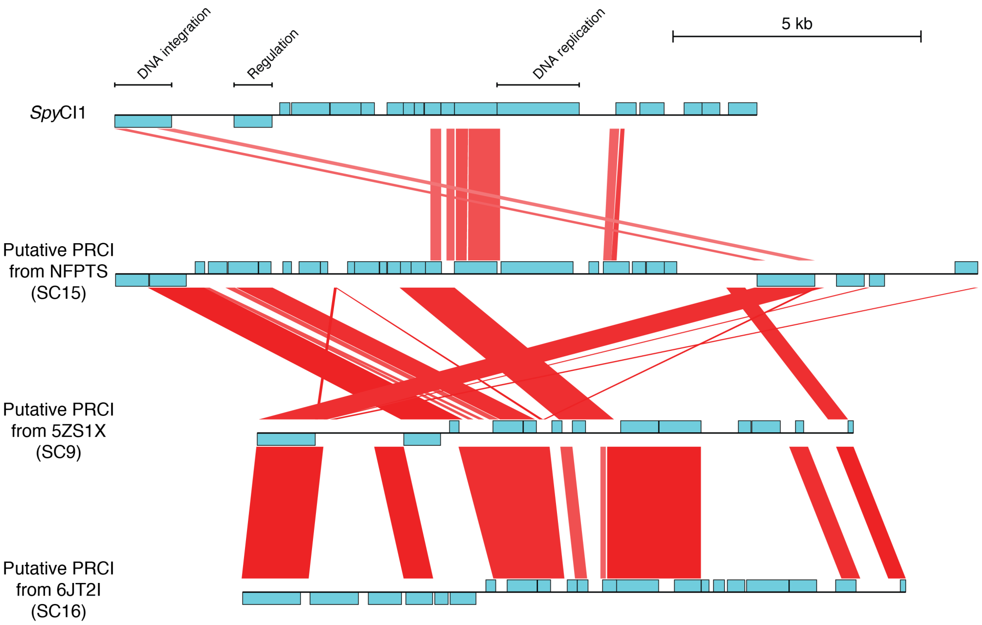

Supplementary Figure 15: Putative PRCIs found in component F, displayed as described in Supplementary Figure 10. At the top is displayed the PRCI *Spy*CI1 (SPy_2122-SPy_2147) from *Streptococcus pyogenes* SF370 [EMBL accession: AE004092]. Beneath are displayed putative PRCIs from three different sequence clusters that exhibit a mosaic pattern of similarity.

Supplementary Figure 16

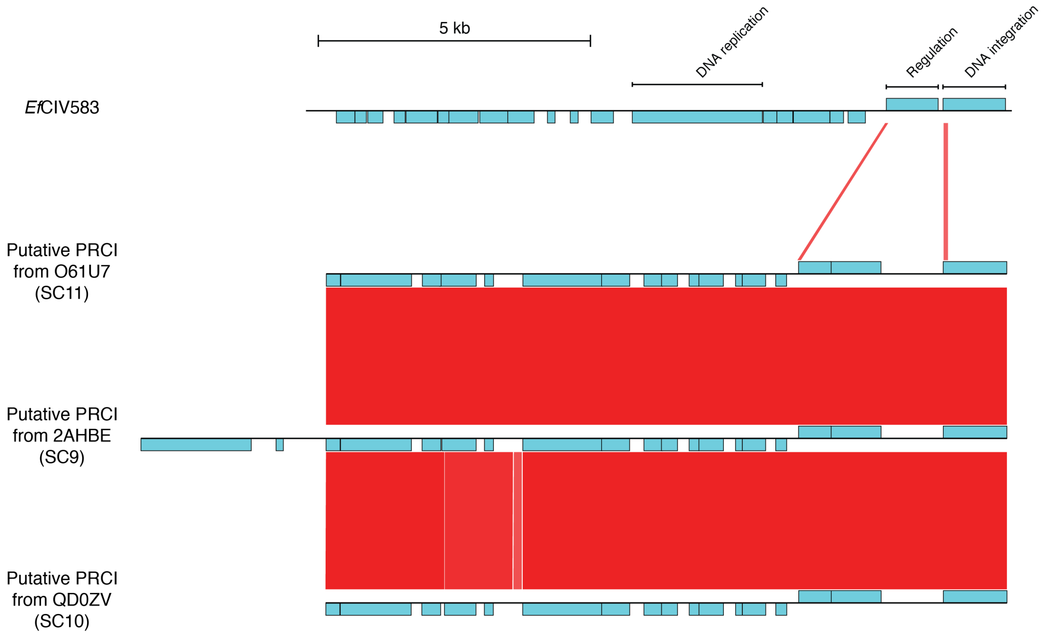

Supplementary Figure 16: Putative PRCIs found in component G, displayed as described in Supplementary Figure 10. Displayed at the top is the enterococcal PRCI *Ef*CIV583 (EF_2936-EF_2955) from *Enterococcus faecalis* V583 [EMBL accession: AE016830], which shows very limited similarity with the putative pneumococcal PRCIs. These three PRCIs are highly similar, despite coming from three different sequence clusters.

Supplementary Figure 17

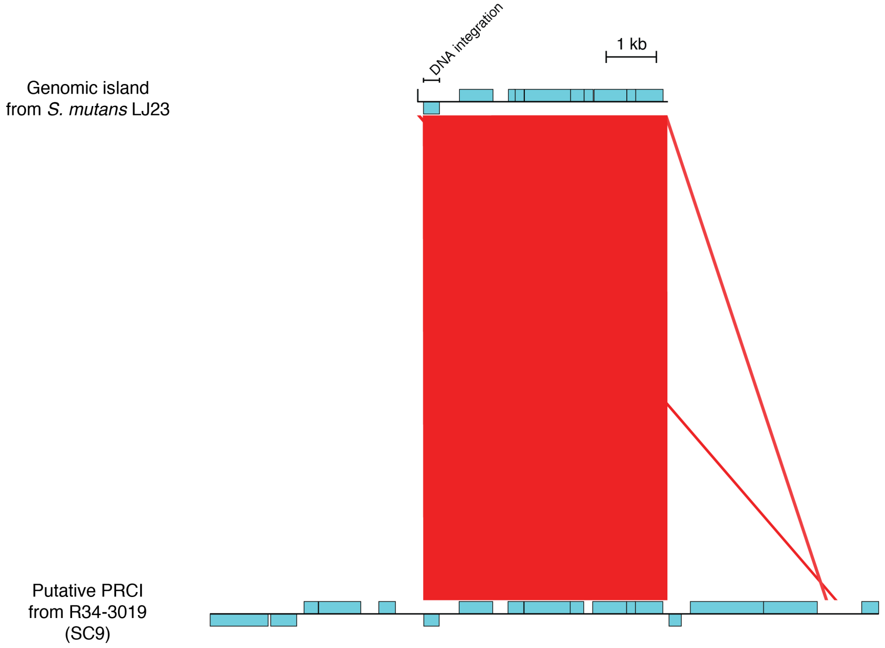

Supplementary Figure 17: Putative PRCI found in component H, displayed as described in Supplementary Figure 10. At the top is displayed a genomic island (SMULJ23_0140-SMULJ23_0150) from *S. mutans* isolate LJ23 [EMBL accession: AP012336]. This region matches the central portion of the putative PRCI from SC9 underneath that is flanked by transposases.

Supplementary Figure 18

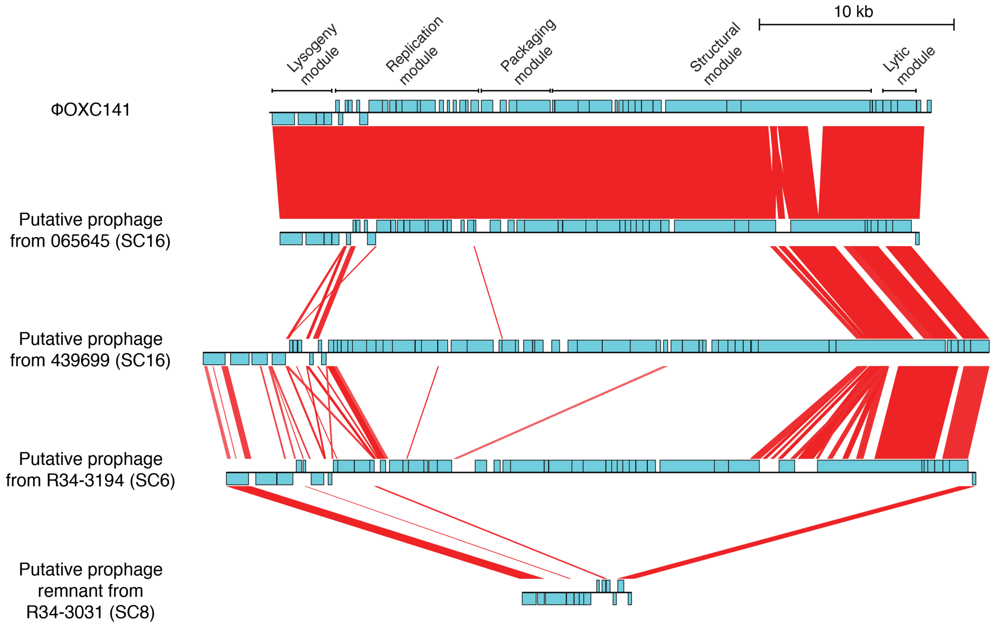

Supplementary Figure 18: Putative prophage-related sequences found in component I, displayed as described in Supplementary Figure 10. At the top is displayed prophage ϕOXC141 (SPNOXC00180-SPNOXC00622) from *S. pneumoniae* ϕOXC141 [EMBL accession: FQ312027]. This is stably associated with serotype 3, clonal complex 180 isolates; correspondingly, a near-identical sequence was identified in such an isolate (065645) within this collection. Prophage from SC6 and SC16 are displayed, both of which exhibit sequence similarity to ϕOXC141 in the lytic module and parts of the structural module. The prophage from isolate R34-3194 is found in the same insertion site as the "prophage remnant", shown at the bottom of the alignment. These two sequences share similarities in their integrase and amidase genes at opposite ends of the virus.

Supplementary Figure 19

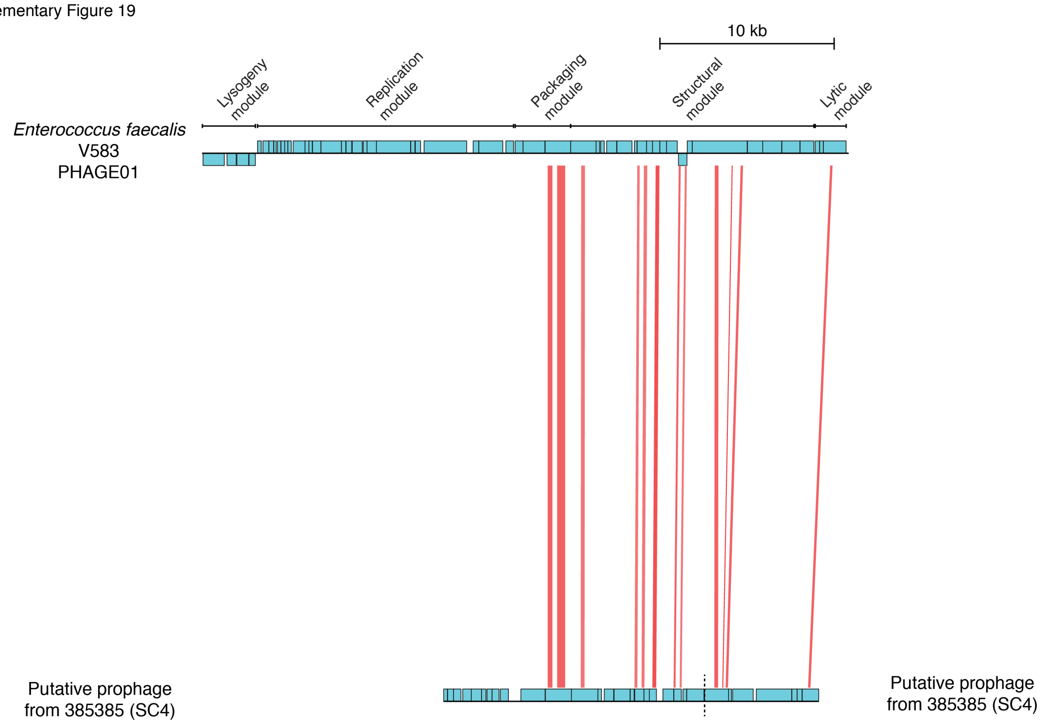

Supplementary Figure 19: Comparison of prophage segments from components J and K with prophage pp1 (EF_0302-EF_0355) from the genome of the vancomycin-resistant *Enterococcus faecalis* isolate V583 [EMBL accession: AE016830], as displayed in Supplementary Figure 10. This prophage could not be assembled in its entirety from the short read data used in this population genomics study; these two segments form separate network components in Figure 3, despite apparently being part of the same MGE, because the assembly breaks (the positions of which are indicated by the vertical dashed line) occurred consistently in the same part of each of the sequences.

Supplementary Figure 20

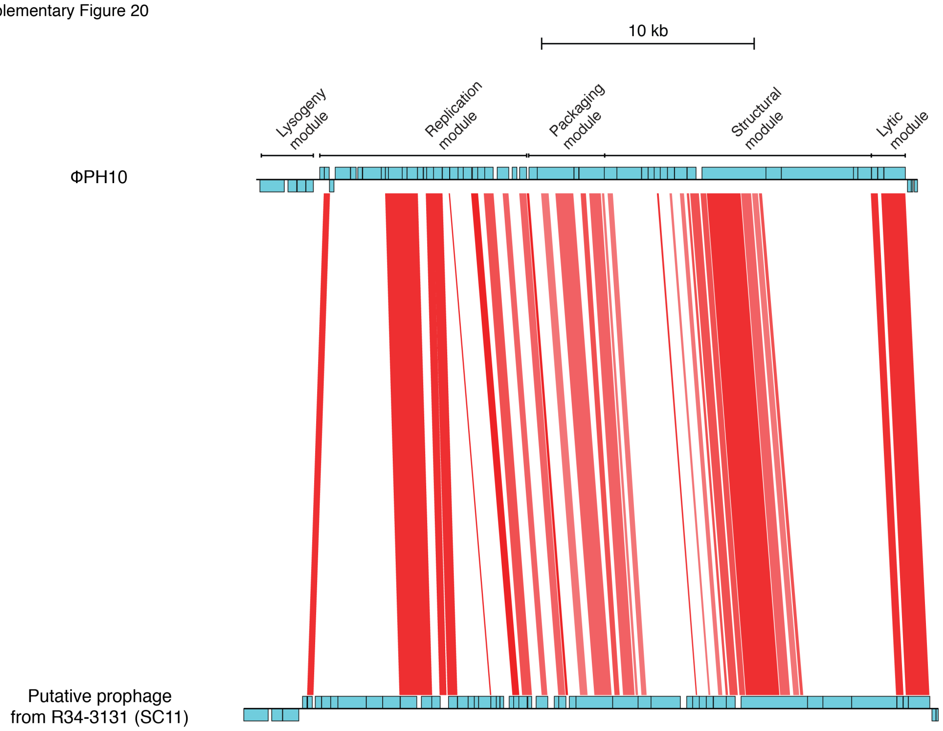

Supplementary Figure 20: Comparison of *S. oralis* prophage ϕPH10 [EMBL accession: FN391954] with the prophage found in component L, as displayed in Supplementary Figure 10.

Supplementary Figure 21

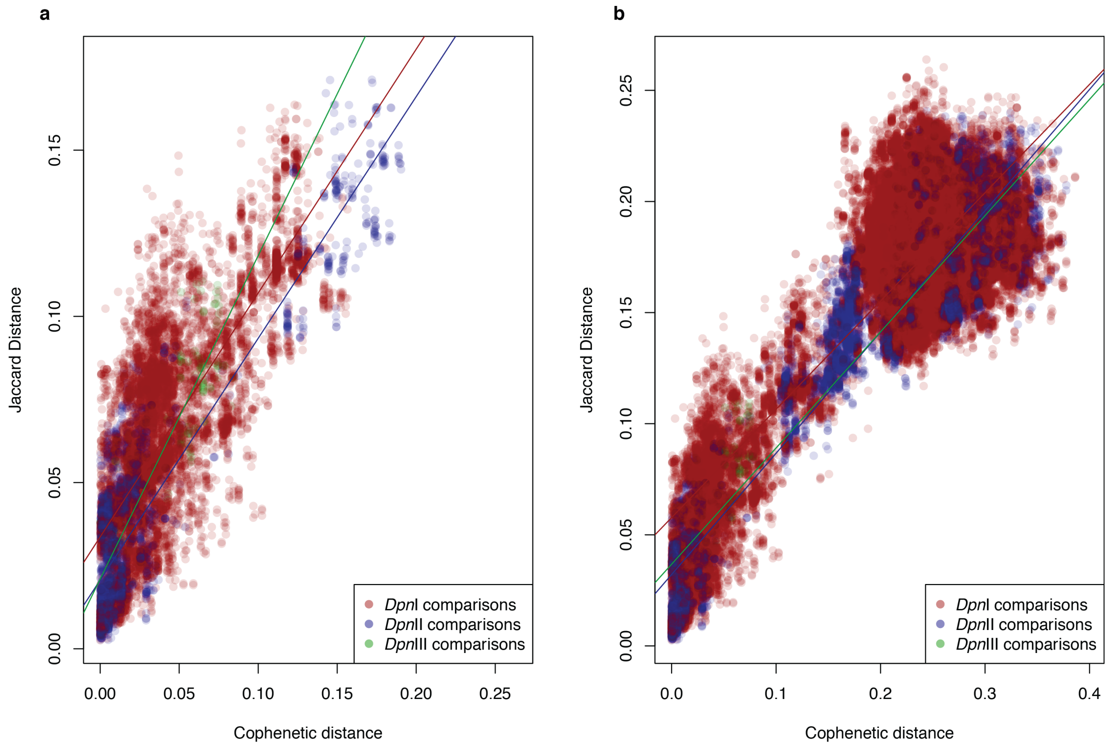

Supplementary Figure 21: The relative rates of diversification of genotypes carrying different *Dpn* loci. (a) Pairwise comparisons between isolates within the same monophyletic sequence cluster that share that same *Dpn* system. Over such short timescales, it is unlikely that the *Dpn* system will have altered during the period over which the isolate pair has diverged. Additionally, as such isolates are closely related, import of sequence through recombination should result in the pairs diverging much more frequently than it causes them to converge, therefore making it easier to use the relative rates of core and accessory genome diversification to detect change in the relevant rates of recombination. As neither *Dpn*I nor *Dpn*II were expected to inhibit the acquisition of genomic islands, it was unsurprising that the relative rate of accessory to core genome diversification was described by a gradient of 0.73 for both isolates sharing *Dpn*I (red line; 95% confidence interval of 0.71-0.74) and *Dpn*II (blue line; 95% confidence interval of 0.72-0.75). By contrast, *Dpn*III seems likely to inhibit the acquisition of any novel GI carrying the motif it

targets, as it is predicted to be a conventional Type II RMS that would cleave such sequences post-integration if they were unmethylated. Hence it is somewhat unexpected that isolates sharing *Dpn*III appeared to diversify their accessory genome most quickly (green line; gradient of 0.97; 95% confidence interval of 0.90-1.0), although this is based on a small sample size. (b) Pairwise comparisons between isolates sharing the same *Dpn* locus. In this plot, all pairwise comparisons between isolates with the same *Dpn* locus are shown, excluding comparisons between SC12 and non-SC12 sequence clusters. This allowed divergence to be measured over longer timescales, with a correspondingly elevated possibility that isolates may have switched between *Dpn* loci for some of the time over which they diverged. Furthermore, it is also more likely that recombination can cause convergence between more distantly related isolate pairs, as well as driving their divergence, making any difference between the systems more difficult to interpret. This analysis found that the isolates sharing *Dpn*II (blue line; gradient of 0.55; 95% confidence interval, 0.54-0.55) or *Dpn*III (green line; gradient of 0.52; 95% confidence interval of 0.51-0.54) diversified their accessory genome at a higher rate relative to those sharing *Dpn*I (red line; 0.49; 95% confidence interval of 0.49-0.49), but variation in their intercept position on the vertical axis meant there was little difference between the lines over the represented timescale of evolution.

Supplementary Figure 22

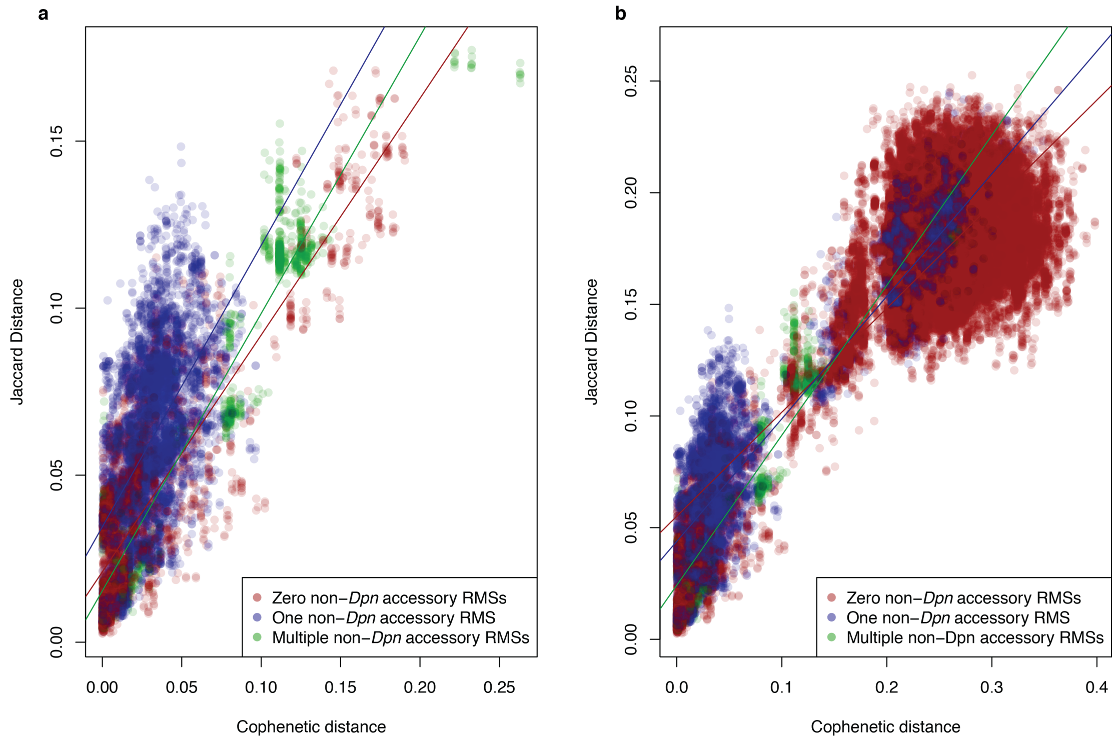

Supplementary Figure 22: The relative rates of diversification of genotypes carrying different numbers of non-*Dpn* accessory RMSs. (a) Pairwise comparisons between isolates within the same monophyletic sequence cluster sharing the same number of non-*Dpn* accessory RMSs. The comparisons represented by the points in this graph will almost always involve isolate pairs that have conserved their complement of non-*Dpn* accessory RMSs over the course of their divergence. This plot indicated that the accessory genome actually appeared to diversify faster in those genotypes with a single putative accessory RMS (blue line; gradient of 0.84; 95% confidence interval of 0.81 to 0.87), or more (green line; 0.83; 95% confidence interval of 0.82 to 0.85), rather than those that lacked any (red line; gradient of 0.71; 95% confidence interval 0.70-0.72). (b) Pairwise comparisons between isolates with the same number of non-*Dpn* accessory RMSs (excluding comparisons between SC12 and non-SC12 sequence clusters). This comparison of more diverse sequences means there was an elevated probability that the nature of the accessory

RMSs may have changed over the period of divergence between a pair of isolates, and that recombinations may cause convergence between pairs rather than divergence. Although there was little substantial difference between the lines over the displayed period of evolution, the gradients describing the diversification of isolates carrying a single non-*Dpn* accessory RMS (blue line; 0.55; 95% confidence interval of 0.55-0.55), or more (green line; 0.67; 95% confidence interval of 0.66-0.68), were again higher than that describing those isolates lacking any such system (red line; 0.47; 95% confidence interval of 0.46-0.47). This suggests that non-*Dpn* RMSs have little impact on the exchange of genomic islands.

Supplementary Figure 23

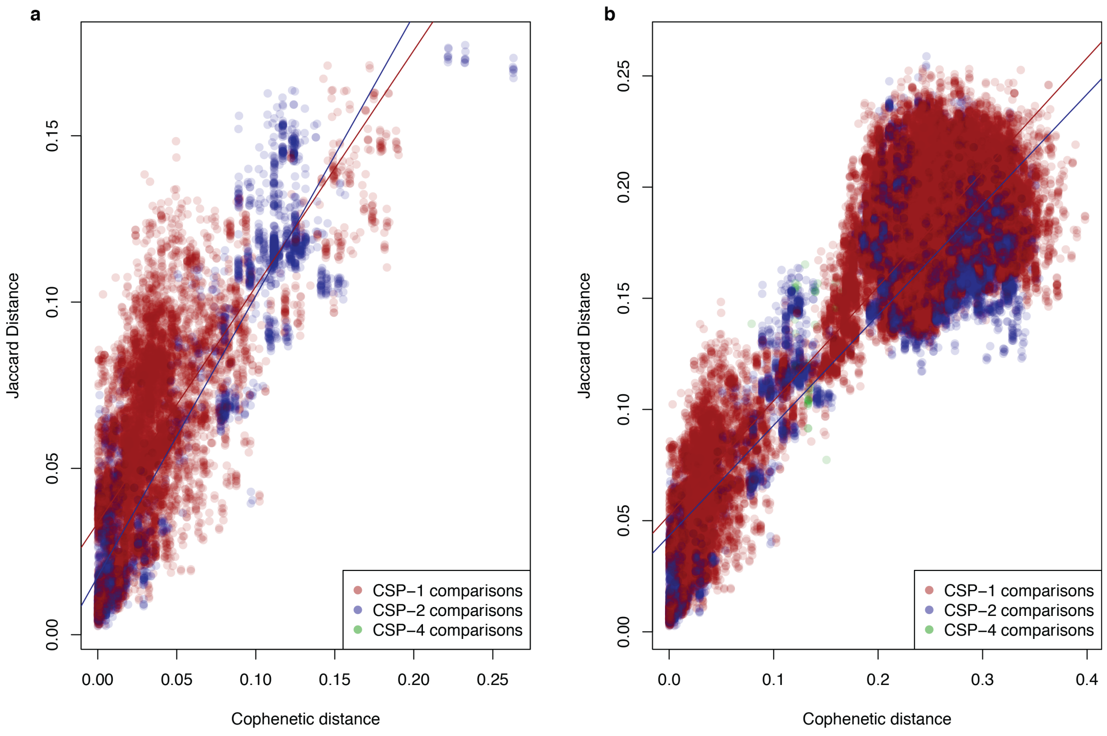

Supplementary Figure 23: The relative rates of diversification of different pherotypes. (a) Pairwise comparisons between isolates of the same pherotype within the same monophyletic sequence cluster. Isolates sharing CSP-2 were associated with a somewhat higher rate of accessory genome diversification (blue line; gradient of 0.84; 95% confidence interval of 0.83 to 0.86) than those sharing CSP-1 (red line; 0.71; 95% confidence interval of 0.70-0.73), but differences in intercept meant this was not substantial over the displayed period of evolution. (b) Pairwise comparisons between isolates of the same pherotype (excluding comparisons between SC12 and other sequence clusters). In this comparison, the two gradients were very similar: 0.51 for isolates sharing CSP-1 (red line; 95% confidence interval of 0.51 to 0.52), and 0.50 for isolates sharing CSP-2 (blue line; 95% confidence interval of 0.49-0.50).

Supplementary Figure 24

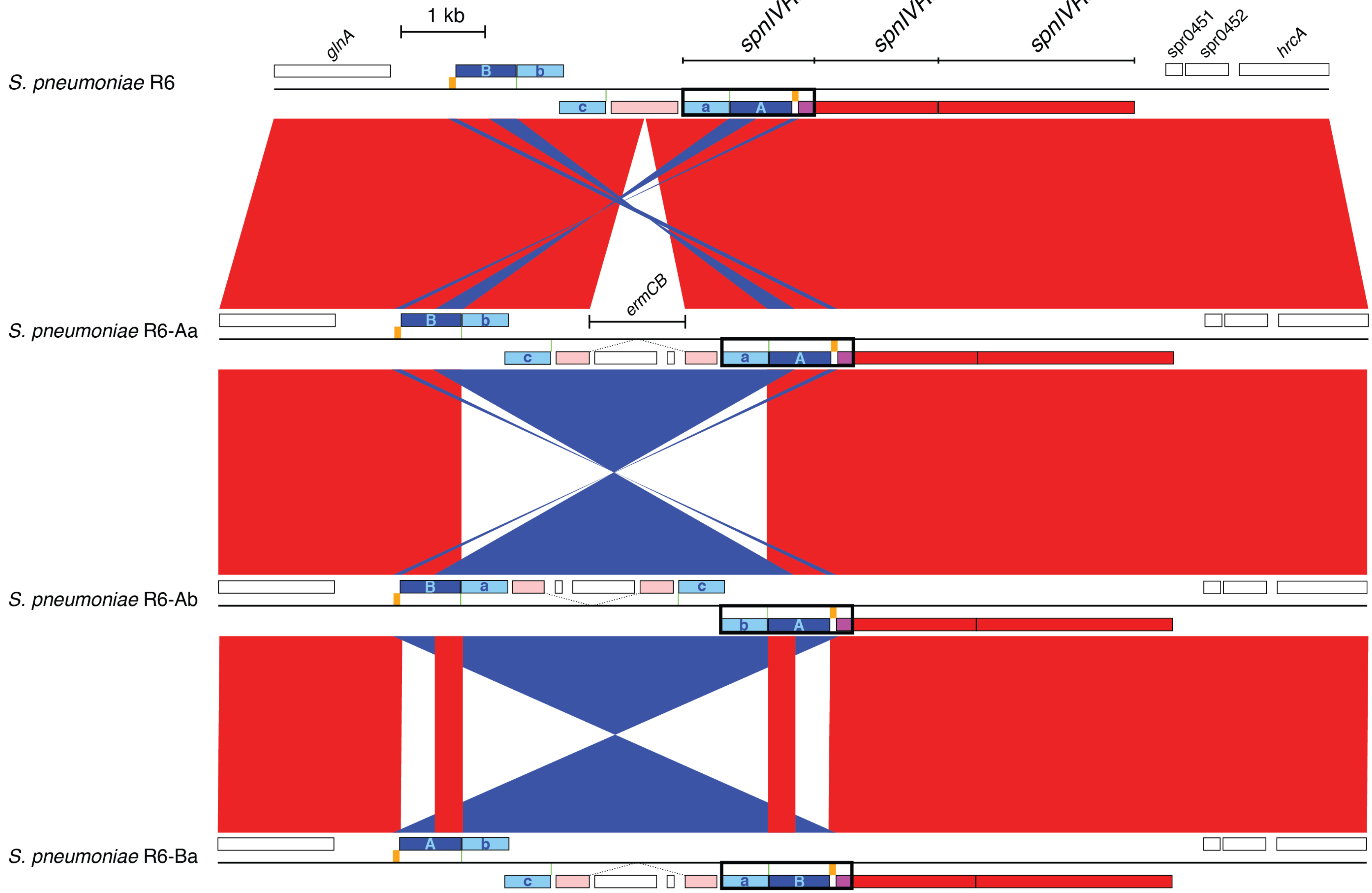

Supplementary Figure 24: Integrase knock out mutants within the *ivr* locus. The native *ivr* locus of *S. pneumoniae* R6 is shown across the top; the red CDSs represent the conserved *spnIVRhsdM* and *spnIVRhsdR* methylase and endonuclease subunit genes. From the 5' to 3', the variable *spnIVRhsdS* gene (highlighted by a black box) is composed of an invariant N terminus (purple box); a repeated sequence recognized by the recombinase (orange box); a 5' TRD-encoding sequence (either A or B; dark blue boxes); a second, shorter repeated sequence (green box); and a 3' TRD-encoding sequence (either a, b or c; light blue box). Inversions occur between the long repeats (orange boxes), exchanging both TRD-encoding sequences of *spnIVRhsdS* for those on the opposite strand, or between the short repeats (green boxes) that exchange only the 3' TRD-encoding sequences. Beneath are *ivr* loci assembled *de novo* from SMRT sequencing of three mutant derivatives of *S. pneumoniae* R6. The *ivr* locus of mutant *S. pneumoniae* R6-Aa is aligned to the native locus, with red bands indicating similar nucleotide sequence in the same orientation in both loci, and blue twisted bands representing similar nucleotide sequence in opposite orientations in the two loci. In *S. pneumoniae* R6 Aa, the *ivrR* recombinase gene was disrupted by the insertion of the *ermCB* macrolide resistance operon, but the sequence of *spnTVRhsdS* was the same as in the original genome. The same insertion was found in the mutants R6-Ab and R6-Ba, albeit with different alleles of *spnTVRhsdS* assembled in each case.

Supplementary Figure 25

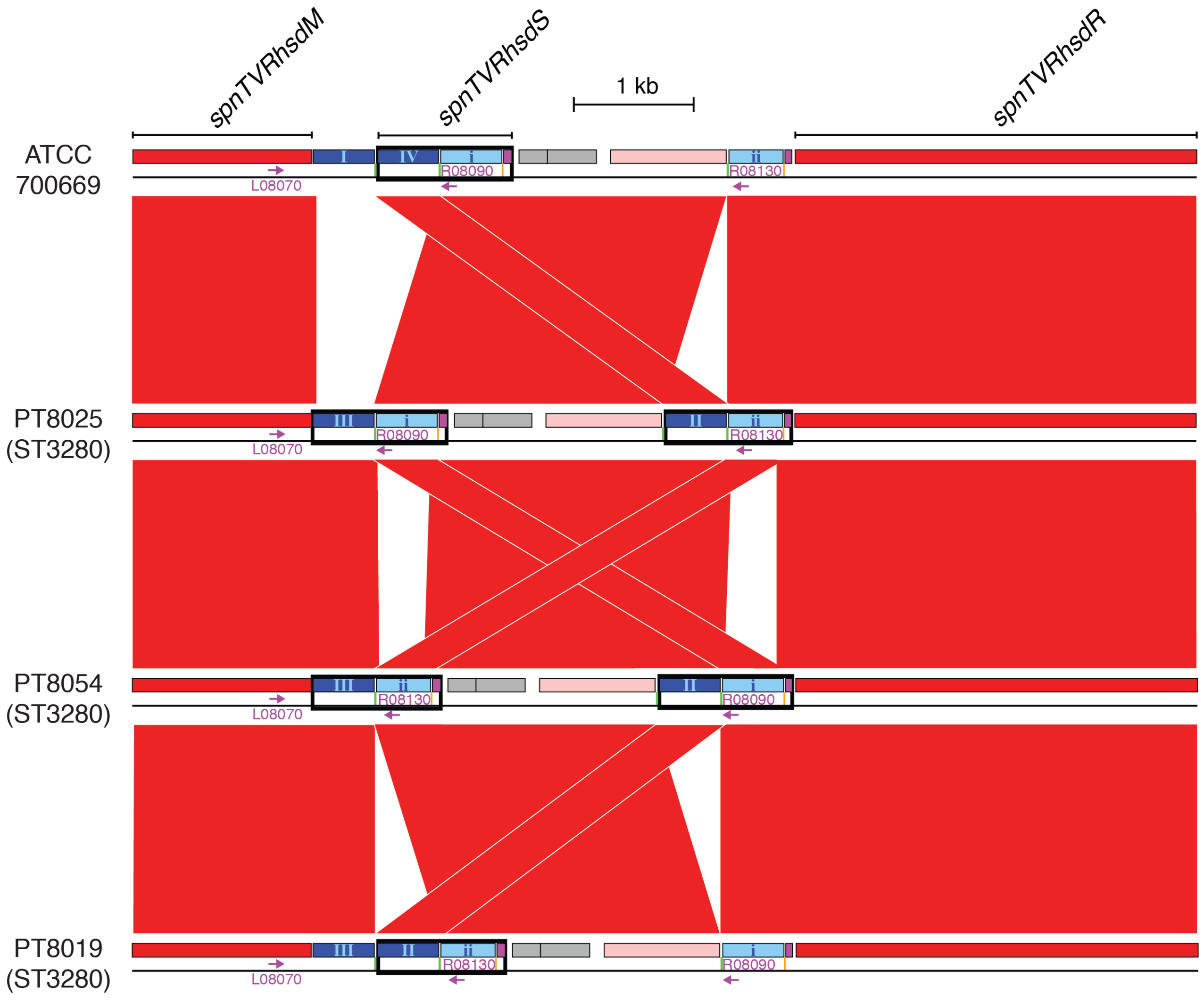

	L08070 R08090	L08070 R08130
ATCC 700669	1450 bp	3922 bp
PT8025 (ST3280)	914 bp	3922 bp
PT8054 (ST3280)	3798 bp	981 bp
PT8019 (ST3280)	3798 bp	1517 bp

Supplementary Figure 25: The *tvr* loci from isolates of sequence type 3280, assembled from Illumina sequence data, displayed as described in Supplementary Figure 24. Three different configurations of the *tvr* locus could be assembled from the five closely-related sequence type 3280 isolates within the collection of genomes, here aligned to that of *S. pneumoniae* ATCC 700669. The red CDSs represent the conserved *spnTVRhsdM* methylase and *spnTVRhsdR* endonuclease genes. Each of the *spnTVRhsdS* TRD-encoding sequences is annotated according to the scheme in Figure 4. Intact *spnTVRhsdS* genes appear to be composed of a 5' TRD-encoding sequence (dark blue boxes), long repeats (green box), 3' TRD-encoding sequence (light blue box), short repeat (orange box) and conserved 3' sequence (purple box); these full-length genes are outlined by black boxes. Purple arrows indicate the position of primers; the size of product expected from the primer pairs L08070 and R08090, and L08070 and R08130, are tabulated to the right of the figure.

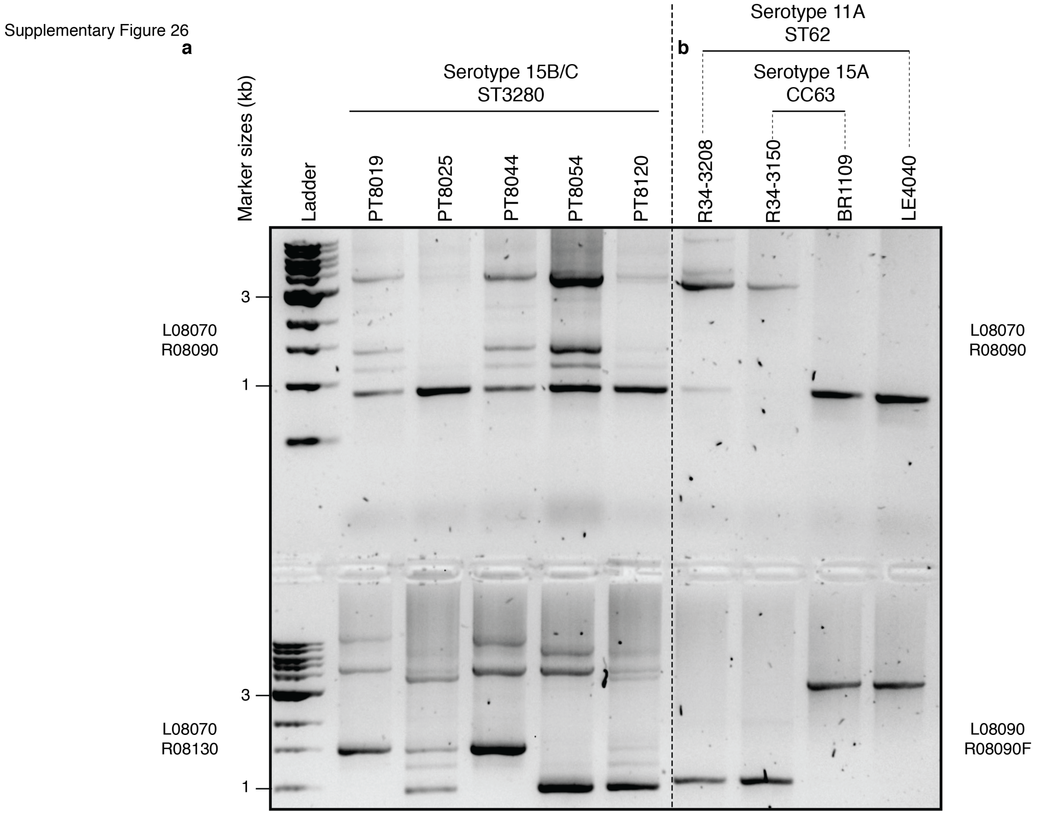

Supplementary Figure 26: Multiple orientations of the *tvr* locus. (a) Configuration of the *tvr* loci in closely-related sequence type 3280 isolates. The top row of lanes show the agarose gel electrophoretic separation of the bands generated by PCR amplification from genomic DNA using the primers L08070 and R08090 with an extension time of 4 min 40 s per thermocycle. As predicted from the assemblies, a prominent band of just under 1 kb was observed for isolate PT8025, and bands of over 3 kb in length for PT8019 and PT8054 (intact *tvr* loci were not assembled for sequence type 3280 isolates PT8044 and PT8120). Multiple smaller bands were also observed with genomic DNA from PT8019 and PT8054, suggesting that there might be shuffling of sequence within the locus. The bottom lanes show the bands generated using the primers L08070 and R08130. In this case, there was a prominent band of just under 1 kb in length for isolate PT8054, as predicted from the genome assembly. The main band in the PT8019 lane was approximately of the expected 1.4 kb size, while in isolate PT8025 the expected band of over 3 kb in length was observed,

as were several shorter bands. Alongside Figure 5, this again suggested the potential for intragenomic recombination. (b) Convergent evolution of the *tvr* locus in SC2 and SC3. The top lanes show the agarose gel electrophoretic separation of the bands generated by PCR amplification of genomic DNA using the primers L08070 and R08090 with an extension time of 4 min 40 s per thermocycle. The serotype 11A, sequence type 62 isolates of SC2 show single bands of very different sizes, as expected from their genome sequence assemblies (Supplementary Figure 27). The serotype 15A, clonal complex 63 isolates of SC3 show the same predicted pattern of dissimilarity; hence the distantly related isolate pairs of R34-3208 and R34-3150, and LE4040 and BR1109, appear to have separately converged upon the same *tvr* loci. The bands generated by PCR amplification using the primer pair L08090 and R08090F, displayed in the lower row of lanes, also provide evidence for this.

Supplementary Figure 27

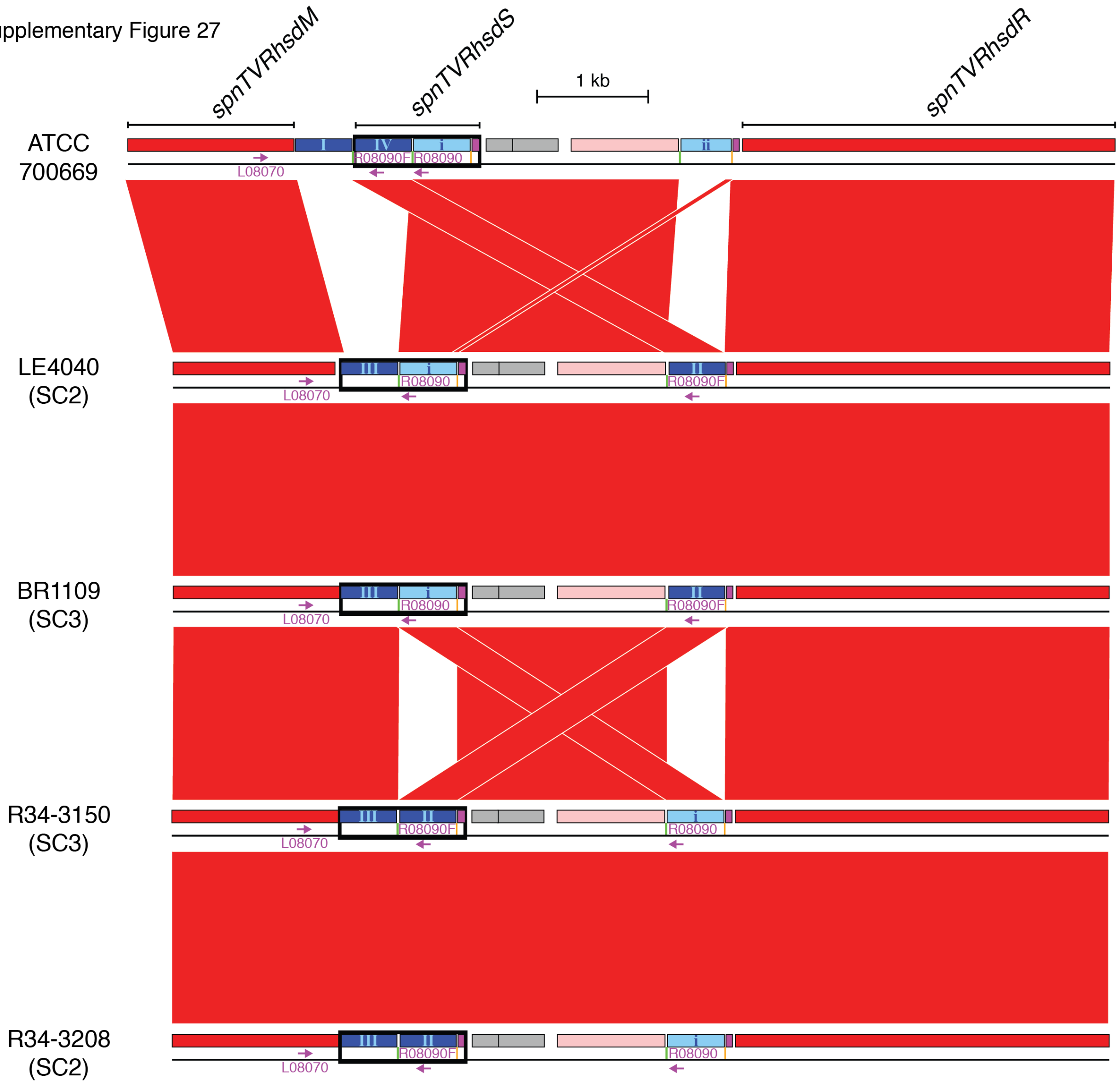

L08070 R08090	L08070 R08090F
1450 bp	1052 bp
914 bp	3457 bp
914 bp	3457 bp
3324 bp	1052 bp
3324 bp	1052 bp

Supplementary Figure 27: The *tvr* loci of SC2 and SC3 isolates, assembled from Illumina sequence data, displayed as in Supplementary Figure 25. The table shows the expected product sizes for the PCR amplification reactions shown in Supplementary Figure 26.

Supplementary Figure 28

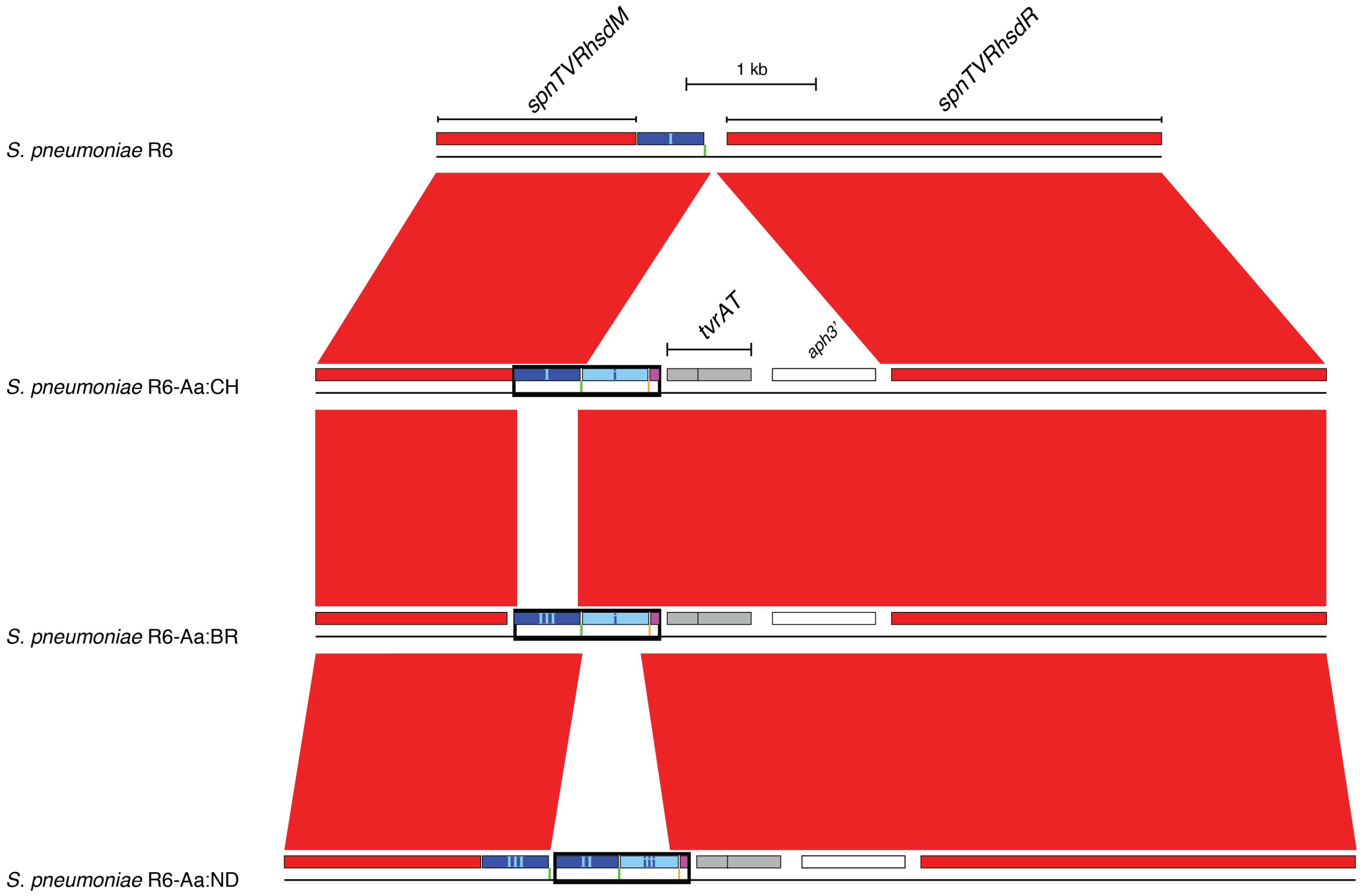

Supplementary Figure 28: Mutant *tvr* loci inserted into *S. pneumoniae* R6-Aa, assembled from SMRT sequence data, displayed as described in Supplementary Figure 25. The wild type *S. pneumoniae* R6 genome has no functional *tvr* locus, as it lacks a full-length *spnTVRhsdS* specificity subunit gene. Each of the inserted *tvr* loci contains an *aph3'* aminoglycoside resistance marker, a toxin-antitoxin system, and an apparently functional *spnTVRhsdS* gene (outlined by a black box). A truncation of the *spnTVRhsdM* CDS in *S. pneumoniae* R6-Aa:BR is evident in this figure, and may have been sufficient to render the system non-functional, based on the results of SMRT sequencing.

Supplementary Figure 29

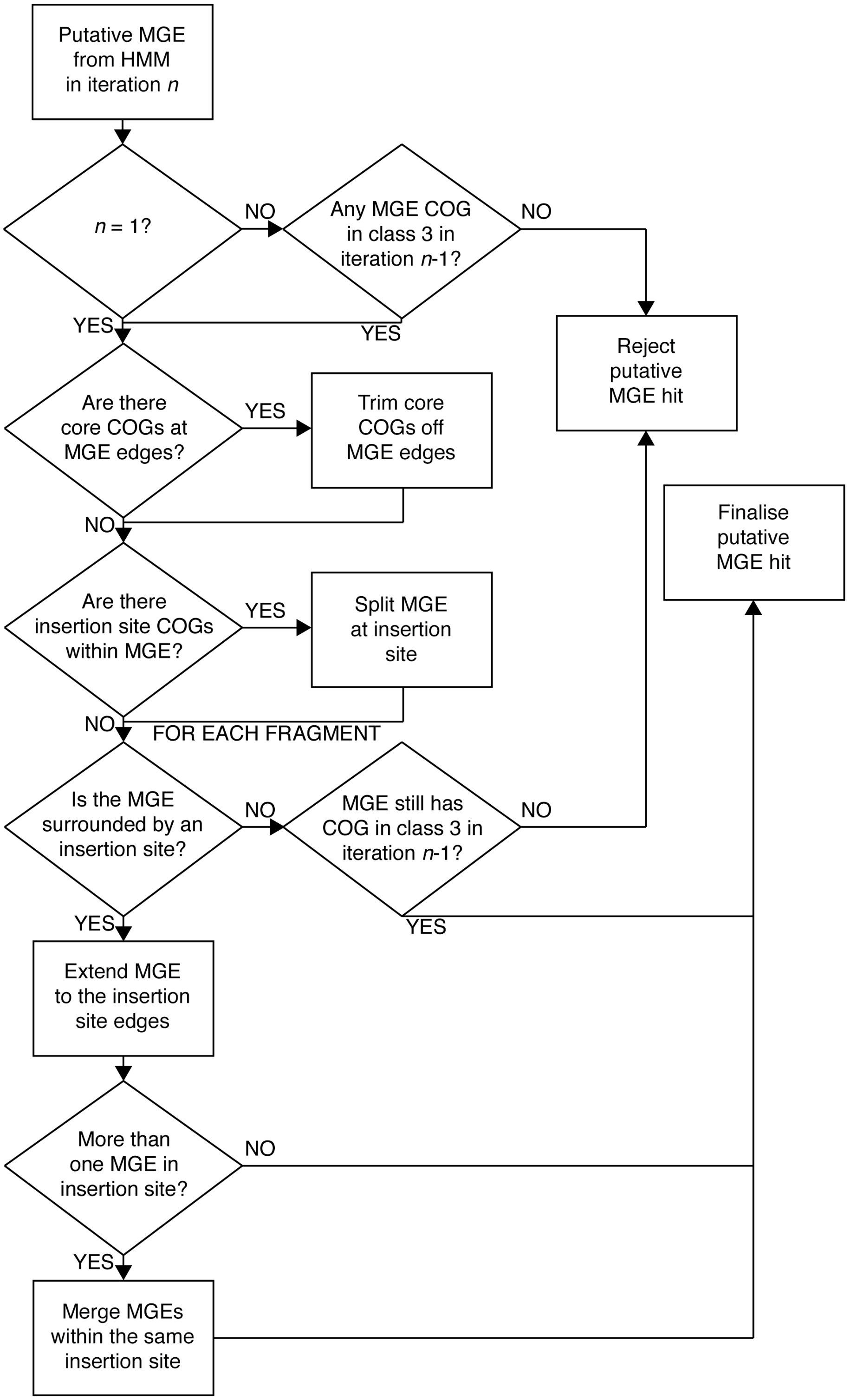

Supplementary Figure 29: Flowchart describing the heuristics used to process the output of the hidden Markov model into the final prediction of putative MGEs.

Supplementary Figure 30

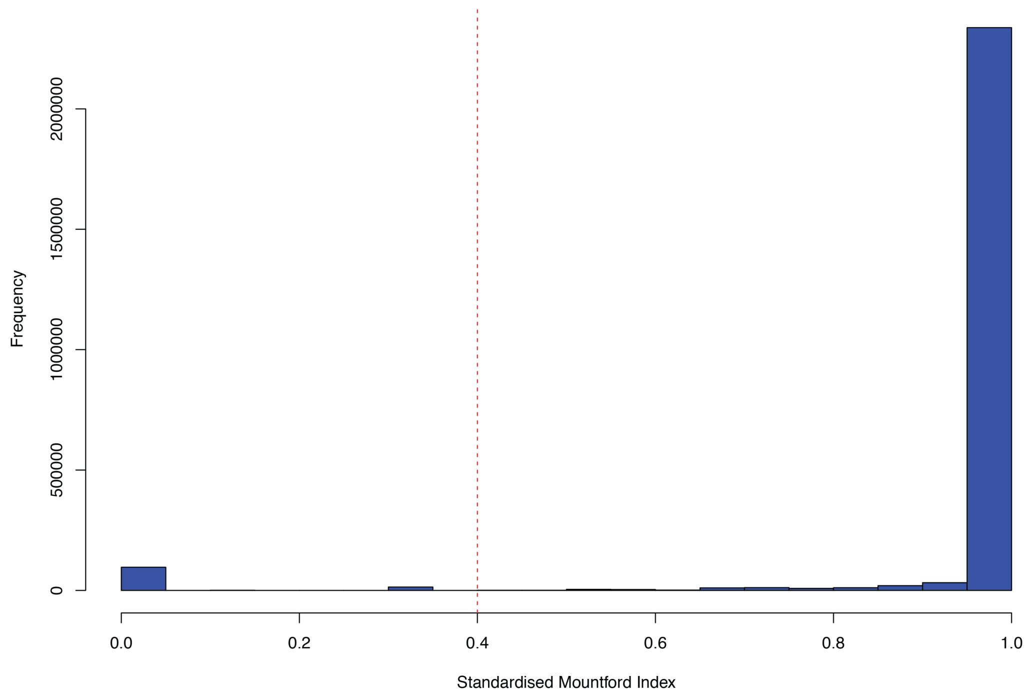

Supplementary Figure 30 Dissimilarities between putative MGEs calculated using the Mountford index. All dissimilarities were divided by their maximal value, ln(2), to standardize them to values between zero and one. All putative MGEs separated by a dissimilarity below the 0.4 threshold indicated by the vertical red dashed line were linked with an edge in the network displayed in Figure 3.

Supplementary Tables

Supplementary Table 1 - Characteristic COGs of the fifteen monophyletic sequence clusters. The classifications are 'CAP' (capsule locus), 'FS' (pseudogene fragment generated by a frameshift mutation), 'GI' (genomic island), 'IS' (IS element), 'MGE' (mobile genetic element), 'PPI' (Pneumococcal Pathogenicity Island 1) and 'PSP' (pneumococcal surface protein, corresponding to either PspA or PspC).

cCOG	Sequence Cluster	Classification	Pfam Domains
CLS02452	1	MGE	SipA,
CLS02453	1	MGE	-
CLS02454	1	MGE	DUF624, Exo_endo_phos, TnpV,
CLS02531	1	PPI	-
CLS03264	1	PPI	-
CLS02593	3	FS	-
CLS02597	3	FS	RepA_N,
CLS02615	3	MGE	Not3, Streptin-Immun,
CLS02617	3	MGE	UvrD_C, UvrD-helicase,
CLS02618	3	MGE	AAA_21, Spc7,

CLS02619	3	MGE	-
CLS02620	3	MGE	DUF4071,
CLS02622	3	MGE	Zeta_toxin,
CLS02623	3	MGE	-
CLS02624	3	MGE	AAL_decarboxy, DUF3990,
CLS02625	3	MGE	DUF3991, Toprim_2, zf-CHC2,
CLS02626	3	MGE	HTH_19,
CLS02627	3	MGE	ABC_membrane, ABC_tran,
CLS02628	3	MGE	-
CLS02629	3	MGE	ABC_tran,
CLS02630	3	MGE	DUF1430,
CLS02631	3	MGE	-
CLS02647	3	MGE	DIX,
CLS02648	3	MGE	-
CLS02649	3	MGE	-
CLS02650	3	MGE	-
CLS02659	3	PSP	RICH, YSIRK_signal,

CLS03302	4	CAP	Hexapep,
CLS03312	4	FS	NAD_binding_10,
CLS03406	4	MGE	-
CLS03407	4	MGE	-
CLS03408	4	MGE	Metallophos,
CLS03409	4	FS	-
CLS03410	4	CAP	Glycos_transf_1, PIGA,
CLS03411	4	CAP	Glycos_transf_1,
CLS03412	4	CAP	Glycos_transf_2,
CLS03413	4	CAP	-
CLS03414	4	CAP	-
CLS03415	4	CAP	Polysacc_synt, SdpI,
CLS03420	4	ZMP	Gram_pos_anchor, Peptidase_M26_C, Peptidase_M26_N,
CLS02875	5	PPI	DUF772,
CLS02876	5	PPI	DDE_Tnp_1,
CLS02930	5	MGE	-

CLS02931	5	MGE	-
CLS02934	5	GI	-
CLS02939	5	FS	AlaDh_PNT_N, PYC_OADA,
CLS02943	5	PPI	-
CLS02944	5	PPI	CW_binding_1, Trypsin_2,
CLS02945	5	PPI	Phage_connect_1,
CLS02946	5	PPI	AAA_21,
CLS02947	5	PPI	UvrD-helicase, Viral_helicase1,
CLS02948	5	PPI	DDE_Tnp_1_6,
CLS02951	5	PPI	BtrH,
CLS02952	5	PPI	DNA_ligase_aden, PP-binding,
CLS02953	5	PPI	-
CLS02954	5	PPI	Pyridoxal_deC,
CLS02955	5	PPI	AMP-binding, DUF4009,
CLS02956	5	PPI	Aminotran_1_2, GlnE,
CLS02957	5	PPI	Pribosyltran,
CLS02959	5	PPI	ABC2_membrane_6,

CLS02960	5	PPI	ABC2_membrane_6,
CLS02961	5	FS	Iso_dh,
CLS02978	5	FS	IMS_C, Sfi1_C,
CLST4865664	5	MGE	-
CLS03060	6	GI	DUF816,
CLS03061	6	GI	Response_reg,
CLS03062	6	GI	HATPase_c,
CLS02667	7	FS	-
CLS02687	7	MGE	-
CLS03219	7	FS	-
CLS03463	7	FS	Gemin7, LeuA_dimer,
CLS03666	7	GI	-
CLS03667	7	GI	DUF4319, Nse5,
CLS03668	7	GI	-
CLS03669	7	GI	-
CLS03671	7	FS	-
CLS03672	7	FS	-

CLS03673	7	MGE	DNA_methylase,
CLS03674	7	FS	Gram_pos_anchor, MucBP,
CLS03675	7	MGE	Helicase_C,
CLS03676	7	MGE	Transketolase_N,
CLS03677	7	CAP	Glycos_transf_1, Glyco_transf_4,
CLS03678	7	CAP	DUF1919,
CLS03679	7	CAP	-
CLS03680	7	CAP	Glycos_transf_2,
CLS03681	7	CAP	-
CLS03682	7	CAP	Glycos_transf_1, Glyco_transf_4,
CLS03683	7	CAP	-
CLS03684	7	CAP	Polysacc_synt,
CLS03685	7	GI	Glyco_hydro_98C,
CLS00087	8	MGE	ProRS-C_2,
CLS00134	8	GI	AAA_13,
CLS00612	8	ZMP	FIVAR, Gram_pos_anchor, Peptidase_M26_C, Peptidase_M26_N,

CLS00614	8	GI	-
CLS01017	8	ZMP	G5, Gram_pos_anchor, Peptidase_M26_C, Peptidase_M26_N,
CLS02894	9	GI	ABC2_membrane_4, FtsX,
CLS02895	9	GI	ABC_tran,
CLS03124	9	FS	Esterase,
CLS03125	9	FS	-
CLS03136	9	GI	AAA_14, AAA_21, PHP, PHP_C,
CLS03137	9	GI	RE_AlwI,
CLS03138	9	GI	Cas_Csa5, MerR_1, MethyltransfD12,
CLS02810	10	PPI	AAA_23, PHP,
CLS02844	10	GI	-
CLS02845	10	GI	HTH_11, Virulence_RhuM,
CLS02846	10	GI	Eco57I, TaqI_C,
CLS02847	10	GI	HATPase_c, HATPase_c_3,
CLS02848	10	GI	-
CLS02849	10	GI	HNH,

CLS02850	10	GI	DNA_methylase,
CLS02851	10	GI	-
CLS01943	11	MGE	Cna_B, Gram_pos_anchor,
CLS02461	11	FS	-
CLS02682	11	FS	Abi,
CLS02886	11	ZMP	G5, Glug, Gram_pos_anchor, Peptidase_M26_C, Peptidase_M26_N, YSIRK_signal,
CLS02887	11	ZMP	G5, Gram_pos_anchor, Peptidase_M26_C, Peptidase_M26_N,
CLS02905	11	FS	-
CLS02906	11	GI	RelA_SpoT,
CLS02907	11	FS	DUF925,
CLS02908	11	IS	DEDD_Tnp_IS110, Transposase_20,
CLS02909	11	CAP	-
CLS02910	11	CAP	Glyphos_transf,
CLS02911	11	CAP	Glycos_transf_2,
CLS02912	11	CAP	Glyco_trans_1_4,
CLS02915	11	FS	Acetyltransf_3,

CLS02919	11	FS	-
CLS02920	11	FS	S4,
CLS02921	11	FS	Peptidase_C15,
CLS02922	11	FS	FGGY_N,
CLST2256674	11	FS	-
CLS02039	12	FS	G5, Gram_pos_anchor,
CLS02040	12	FS	-
CLS02049	12	GI	-
CLS02050	12	ZMP	G5, Gram_pos_anchor, Peptidase_M26_C, Peptidase_M26_N,
CLS02053	12	MGE	Phage-Gp8, RepA_N,
CLS02054	12	MGE	DNA_methylase,
CLS02055	12	MGE	-
CLS02056	12	MGE	Abi,
CLS02080	12	GI	DUF1542,
CLS02084	12	PPI	DUF2851,
CLS02085	12	PPI	-

CLS02086	12	PPI	AAA_23, Chrome_Resist, SMC_N,
CLS02089	12	MGE	-
CLS02090	12	MGE	Phage_integrase,
CLS02099	12	ZMP	G5, Gram_pos_anchor, Peptidase_M26_C, Peptidase_M26_N,
CLS02100	12	ZMP	Peptidase_M26_C,
CLS02101	12	GI	Dak2,
CLS02102	12	GI	Dak1,
CLS02103	12	GI	CRISPR_Cas2, TetR_N,
CLS02104	12	GI	Dak1,
CLS02105	12	GI	DUF1706,
CLS02106	12	GI	FliB,
CLS02117	12	GI	CAP, CW_binding_1,
CLS02134	12	GI	CW_binding_1, G5, Trypsin, Trypsin_2,
CLS02137	12	FS	DUF939, DUF939_C,
CLS02146	12	FS	-
CLS02147	12	FS	MatE,

CLS02151	12	GI	DDE_Tnp_1, MRP-S28, Rep-A_N,
CLS02152	12	GI	-
CLS02192	12	GI	Amidase_2,
CLS02200	12	MGE	-
CLS02201	12	MGE	-
CLS02202	12	MGE	RPA_C, SelB-wing_3,
CLS02203	12	MGE	RepA_N,
CLS02204	12	MGE	-
CLS02208	12	MGE	Recombinase, Resolvase, Zn_ribbon_2, Zn_ribbon_recom,
CLS02209	12	MGE	DUF4368, Recombinase, Resolvase, Spc7,
CLS02210	12	MGE	Recombinase, Resolvase, Zn_ribbon_recom,
CLS02211	12	MGE	-
CLS02212	12	MGE	HTH_3,
CLS02215	12	MGE	DUF772,
CLS02216	12	MGE	-
CLS02217	12	MGE	-

CLS02218	12	MGE	Relaxase, Ribosomal_L1, Streptin-Immun,
CLS02219	12	MGE	DUF217,
CLS02222	12	MGE	-
CLS02223	12	MGE	-
CLS02225	12	MGE	Zeta_toxin,
CLS02226	12	MGE	-
CLS02227	12	MGE	-
CLS02228	12	MGE	DUF3991, Toprim_2, zf-CHC2,
CLS02229	12	MGE	-
CLS02232	12	GI	-
CLS02233	12	GI	DUF4085, HicB, Matrilin_ccoil, UPF0150,
CLS02234	12	GI	Phage_integrase,
CLS02240	12	GI	Gram_pos_anchor, Pex14_N,
CLS02241	12	GI	-
CLS02242	12	GI	Big_4,
CLS02243	12	GI	Glyco_hydro_2, Glyco_hydro_2_C, Glyco_hydro_2_N,

CLS02244	12	GI	G5, Peptidase_M26_C, YSIRK_signal,
CLS02245	12	GI	Acid_phosphat_B, Cobalamin_bind,
CLS02247	12	MGE	-
CLS02256	12	MGE	RHH_1,
CLS02262	12	MGE	-
CLS02273	12	MGE	CD20, Claudin_2, Cyto_ox_2, DUF1772, DUF2232, DUF3862, DUF4131, DUF4190, DUF981, NKAIN, Virul_fac_BrkB, Wzy_C, YibE_F,
CLS02275	12	MGE	-
CLS02276	12	MGE	DUF829, NTP_transf_2,
CLS02277	12	MGE	-
CLS02319	12	MGE	Baculo_PEP_C, Peptidase_S74,
CLS02322	12	MGE	-
CLS02323	12	MGE	Helicase_C, Methyltransf_26, SNF2_N,
CLS02324	12	MGE	-
CLS02325	12	MGE	efhand,
CLS02326	12	MGE	GbpC, Gram_pos_anchor,
CLS02327	12	MGE	DUF4095,

CLS02328	12	MGE	DUF1814,
CLS02332	12	MGE	HNH_4, Intron_maturas2, RVT_1,
CLS02349	12	FS	N6_Mtase,
CLS03472	13	FS	-
CLS02883	14	PPI	ABC2_membrane_6,
CLS03296	14	ZMP	G5, Gram_pos_anchor, Peptidase_M26_C, Peptidase_M26_N,
CLS03540	14	GI	DUF955,
CLS03541	14	GI	DUF4411,
CLS03543	14	FS	ABC_sub_bind,
CLS03248	15	GI	Bacteriocin_IIc, FAD_binding_4, Gly-zipper_OmpA,
CLS03249	15	GI	Bacteriocin_IIc,
CLS03250	15	GI	Abi,
CLS03251	15	GI	-
CLS03252	15	ZMP	G5, Gram_pos_anchor, Peptidase_M26_C, Peptidase_M26_N,

Supplementary Table 2 - Characteristics of the MGE network groups

Statistic	Prophage (without remnant)	Prophage (including remnant)	ICE	PRCI
Number of Nodes	538	672	1,083	471
Clustering Coefficient	0.560	0.645	0.887	0.965
Network Density	0.0460	0.0681	0.141	0.115
Network Heterogeneity	1.38	1.14	0.978	0.731
Average Number of Neighbours	24.7	45.7	153	53.9
Network Centralisation	0.417	0.302	0.235	0.133

Supplementary Table 3 - Pfam domains used to search for novel restriction modification systems, representing a manually curated list of the domains identified with the search term 'restriction modification system'.

Pfam Identifier	Name
PF10592.4	AIPR
PF03230.8	Antirestrict
PF07275.6	ArdA
PF02923.10	BamHI
PF11564.3	BpuJI_N
PF07832.6	Bse634I
PF06616.6	BsuBI_PstI_RE
PF12106.3	Colicin_C
PF04556.7	DpnII
PF06044.7	DRP
PF08011.6	DUF1703
PF08819.6	DUF1802
PF12957.2	DUF3846
PF13020.1	DUF3883
PF13643.1	DUF4145
PF04411.7	DUF524
PF07669.6	Eco57I
PF08463.5	EcoEI_R_C
PF12008.3	EcoR124_C

PF02963.11	EcoRI
PF09019.6	EcoRII-C
PF09217.5	EcoRII-N
PF09195.6	Endonuc-BglII
PF09194.5	Endonuc-BsobI
PF09233.6	Endonuc-EcoRV
PF09254.6	Endonuc-FokI_C
PF09226.6	Endonuc-HincII
PF09208.5	Endonuc-MspI
PF09225.5	Endonuc-PvuII
PF02980.11	FokI_C
PF02981.10	FokI_N
PF08797.6	HIRAN
PF12161.3	HsdM_N
PF04313.9	HSDR_N
PF13588.1	HSDR_N_2
PF09509.5	Hypoth_Ymh
PF14354.1	Lar_restr_allev
PF10117.4	McrBC
PF01420.14	Methylase_S
PF04471.7	Mrr_cat
PF13156.1	Mrr_cat_2
PF14338.1	Mrr_N
PF02384.11	N6_Mtase

PF09126.5	NaeI
PF09015.5	NgoMIV_restric
PF12183.3	NotI
PF08684.5	ocr
PF11463.3	R-HINP1I
PF04002.10	RadC
PF11058.3	Ral
PF04851.10	ResIII
PF11407.3	RestrictionMunI
PF11487.3	RestrictionSfiI
PF09545.5	RE_AccI
PF09665.5	RE_Alw26IDE
PF09491.5	RE_AlwI
PF09499.5	RE_ApaLI
PF09549.5	RE_Bpu10I
PF09504.5	RE_Bsp6I
PF09552.5	RE_BstXI
PF09516.5	RE_CfrBI
PF09517.5	RE_Eco29kI
PF09553.5	RE_Eco47II
PF09554.5	RE_HaeII
PF09556.5	RE_HaeIII
PF09518.5	RE_HindIII
PF09519.5	RE_HindVP

PF09561.5	RE_HpaII
PF09563.5	RE_LlaJI
PF09562.5	RE_LlaMI
PF09567.5	RE_MamI
PF09568.5	RE_MjaI
PF09564.5	RE_NgoBV
PF09565.5	RE_NgoFVII
PF09521.5	RE_NgoPII
PF09522.5	RE_R_Pab1
PF09566.5	RE_SacI
PF09569.5	RE_ScaI
PF09570.5	RE_SinI
PF09573.5	RE_TaqI
PF09572.5	RE_XamI
PF09571.5	RE_XcyI
PF13707.1	RloB
PF06300.7	Tsp45I
PF12564.3	TypeIII_RM_meth
PF05685.7	Uma2
PF04555.8	XhoI
PF09520	RE_MjaII

Supplementary Table 4 – Putative accessory restriction modification systems, displayed in Figure 4. These do not include a putative Type IV RMS, annotated in REBASE[2], as the predicted endonuclease was not identified using the domains listed in Supplementary Table 3; the putative methylase of this Type IV RMS was ubiquitous across the collection, whereas the putative endonuclease was present in all isolates except most representatives of SC9.

Name/Accession Code	Type of System	Endonuclease COG	Methylase COG
*Dpn*I	II	CLS01600	CLS01599
*Dpn*II	II	CLS02664	CLS02665/6
*Dpn*III	II	CLS03474	CLS03475
LK020705	II	CLS01068	CLS01069
LK020706	II	CLS1068	CLS02116
LK020707	II	CLS02342	CLS02343
LK020708	II	CLS02525	CLS02526
LK020709	II	CLS03137	CLS03138
LK020710	II	CLS03173	CLS03172
LK020711	I	CLS03937	CLS03933
LK020712	II	CLS98944	CLS98943

Supplementary Table 5 – Methylated motifs detected by SMRT sequencing (emboldened bases indicate sites of methylation)

Isolate	Motif (embolded adenine bases indicate site of methylation)	Methylated Site	Modification Type	Number of Motifs Detected	Number of Motifs in Genome	RMS Causing Methylation
**R6**	TCG**A**G	4	m6A	1,506	1,510	Type II RMS
	TCTAG**A**	6	m6A	643	646	Type II RMS
	C**A**GNNNNNNNNTTYG	2	m6A	716	718	*ivr* locus
	CRA**A**NNNNNNNNCTG	4	m6A	713	718	*ivr* locus
**R6-Aa**	TCG**A**G	4	m6A	1,484	1,517	Type II RMS
	TCTAG**A**	6	m6A	631	642	Type II RMS
	C**A**GNNNNNNNNTTYG	2	m6A	711	717	*ivr* locus
	CRA**A**NNNNNNNNCTG	4	m6A	705	717	*ivr* locus
**R6-Ab**	TCTAG**A**	6	m6A	564	646	Type II RMS
	TCG**A**G	4	m6A	1,315	1,527	Type II RMS
	CRA**A**NNNNNNNNNTTC	4	m6A	875	1,035	*ivr* locus
	GA**A**NNNNNNNNNTTYG	3	m6A	873	1,035	*ivr* locus
**R6-Ba**	TCG**A**G	4	m6A	1,509	1,513	Type II RMS
	TCTAG**A**	6	m6A	644	646	Type II RMS

	C**A**CNNNNNNNCTG	2	m6A	430	431	*ivr* locus
	C**A**GNNNNNNNGTG	2	m6A	429	431	*ivr* locus
**R6-Aa-BR**	TCG**A**G	4	m6A	1,507	1,518	Type II RMS
	TCTAG**A**	6	m6A	640	646	Type II RMS
	C**A**GNNNNNNNNTTYG	2	m6A	717	720	*ivr* locus
	CRA**A**NNNNNNNNCTG	4	m6A	708	720	*ivr* locus
**R6-Aa-CH**	TCTAG**A**	6	m6A	627	650	Type II RMS
	TCG**A**G	4	m6A	1,481	1,550	Type II RMS
	C**A**GNNNNNNNNTTYG	2	m6A	712	719	*ivr* locus
	CRA**A**NNNNNNNNCTG	4	m6A	697	719	*ivr* locus
	GAT**A**NNNNNDRTC	4	m6A	276	539	*tvr* locus
	G**A**YNNNNNNTATC	2	m6A	315	721	*tvr* locus
	GAT**A**NNNDNCRTC	4	m6A	37	146	*tvr* locus
**R6-Aa-ND**	TCTAG**A**	6	m6A	643	646	Type II RMS
	TCG**A**G	4	m6A	1,510	1,518	Type II RMS
	C**A**GNNNNNNNNTTYG	2	m6A	722	726	*ivr* locus
	CRA**A**NNNNNNNNCTG	4	m6A	720	726	*ivr* locus
	GG**A**NNNNNNNTGA	3	m6A	1,104	1,108	*tvr* locus

	TC**A**NNNNNNNTCC	3	m6A	1,101	1,108	*tvr* locus
	AAAAWN**A**GGNNT	7	unknown	26	158	Unknown

Supplementary Table 6 – Assessing the robustness of MGE identification to parameter changes.

ν	σ (COG^{-1})	ε (COG^{-1})	Number of MGE fragments	Median length of MGE fragments (COGs; range in parentheses)	Number of MGE CDSs	Number of class 1 MGE COGs (MGE class 1 CDS/Total class 1 CDSs)	Number of class 2 MGE COGs (MGE class 2 CDS/Total class 2 CDSs)	Number of class 3 MGE COGs (MGE class 3 CDS/Total class 3 CDSs)	Strongly discordant COGs with final analysis
100	0.0005	0.05	2,260	31 (2-91)	37,670	3,621 (0/1,132,007)	513 (2,453/5,8930)	1,133 (35,217/35,460)	-
10	0.0005	0.05	2,301	66 (2-101)	40,530	3,587 (0/1,121,778)	528 (5,002/6,8885)	1,152 (35,528/35,734)	3
50	0.0005	0.05	2,260	31 (2-91)	37,726	3,617 (0/1,131,903)	517 (2,509/5,9034)	1,133 (35,217/35,460)	0
500	0.0005	0.05	2,312	13.5 (1-91)	37,794	3,611 (0/1,139,199)	500 (2,424/5,1671)	1,156 (35,370/35,527)	13
1000	0.0005	0.05	2,383	15 (1-91)	38,007	3,597 (0/1,139,077)	512 (2,413/5,1640)	1,158 (35,594/35,680)	13
100	0.005	0.05	2,346	6 (1-91)	37,907	3,606 (0/1,139,086)	520 (2,428/5,1681)	1,141 (35,479/35,630)	4
100	0.00005	0.05	2,327	6 (2-101)	40,476	3,595 (0/1,122,444)	521 (4,896/6,8222)	1,151 (35,580/35,731)	3
100	0.0005	0.5	2,298	11.5 (1-91)	37,751	3,616 (0/1,139,210)	502 (2,422/5,1678)	1,149 (35,329/35,509)	8
100	0.0005	0.005	2,256	36.5 (2-91)	37,723	3,616 (0/1,125,631)	518 (2,511/6,5306)	1,133 (35,212/35,460)	0

Supplementary Table 7 - Accession codes of MGE assemblies submitted to the European Nucleotide Archive

Type	Isolate	ENA Accession Code
ICE	187406	LK020689
ICE	397079	LK020692
ICE	403790	LK020693
ICE	462746	LK020683
ICE	WVCE6	LK020696
ICE	0FQ8K	LK020697
ICE	6U8ZJ	LK020703
ICE	J9GMM	LK020702
ICE	NFPTS	LK020698
ICE	R34-3012	LK020680
ICE	R34-3184	LK020685
ICE	R34-3225	LK020687
ICE	RWZJE	LK020701
ICE	UB6XH	LK020704
Phage	065645	LK020688
Phage	385385	LK020690, LK020691
Phage	439699	LK020684
Phage	R34-3031	LK020676
Phage	R34-3131	LK020694
Phage	R34-3194	LK020686

PRCI	446376	LK020682
PRCI	2AHBE	LK020715
PRCI	5ZS1X	LK020700
PRCI	6JT2I	LK020695
PRCI	NFPTS	LK020699
PRCI	O61U7	LK020713
PRCI	QD0ZV	LK020714
PRCI	R34-3013	LK020681
PRCI	R34-3016	LK020678
PRCI	R34-3019	LK020679
PRCI	R34-3053	LK020677

Supplementary Table 8 – List of primer sequences

Primer Name	Primer Sequence
L08070	GCGGATGGTTTAAGTTTGGA
R08090	TTTTTGCCCCTATCACCATC
RC08090	TGGTGATAGGGGCAAAAATT
R08090F	ACCCGACCACGAAATAAGAA
R08130	AATGCCATTTCCACCATAGG
R08140	TTTCAAGCTATTTCTCCACACTTTT
ND001	AGGGGTTTTTCAGTGGTGTG
Lint	CGCGGGCCCGCATGTAGAAATCGGTTATTTTGA
Linr	CGCGGATCCACTTACACGAGCCCCAGTTG

ermBF	CTAGGATCCCGCGGATCCTGGAAATAAGACTTAGAAGCAAACTT
ermBR	CTCGGGCCCTCTCCATTCCCTTTAGTAACGTGT
LUpVL	TGCAGGAGTATTTTGGCTGA
LDwnVL	TGCGGGCCCAAAAGTGTGGAGAAATAGCTTGAAA
RUpVL	CGCGGATCCAAAAAGAGACAATATCAGTTTCTGCAT
RDwnVL	CGGTTCGGACCATCAAGTA
kanL	GCTGGATTTGAATGAGCACAAG
kanR	GGGCCCGGCATCTACATTCTCCTGTGT
R6hsdSL	GCTCGCTCAGTGTAGTTTTAGGA
R6hsdSR	TGGGAATGGGTGAGGTTAAA

Supplementary Methods

Input dataset

The previously described dataset consisted of 1,231,516 putative coding sequences (CDSs) across 101,919 contigs in 616 draft assemblies[3]. These were clustered into 5,442 clusters of orthologous sequences (COGs). In order to provide information on the likely biological function of these COGs, a representative of each (selected as being the closest to the median length) was scanned for functional domains using the Pfam database[4].

In order to increase the information on linkage between CDSs, the contigs from each of the draft assemblies analysed previously were organised into scaffolds using SSPACE2[5], with an insertion size of 500 bp and an error ratio of 0.9. All scaffolds that contained only a single CDS were ignored, resulting in an overall dataset of 616 assemblies containing a total of 25,191 scaffolds, which encoded 1,221,776 CDSs representing 5,267 COGs. Of these, 1,562 were designated as 'core COGs', as they were present in at least 90% of the assemblies with an overall frequency that did not exceed that of the number of isolates by more than 11%.

Fifteen manually curated draft sequences, one for each monophyletic sequence cluster, were annotated in the original description of this dataset[3]. This identified 52 putative mobile genetic elements (MGEs), which were supplemented with the three identified in the reference sequence, *S. pneumoniae* ATCC 700669: ICE*Sp*23FST81,

prophage ϕMM1-2008, and a prophage remnant[6]. The apparently ICE-derived genome island PPI-1 was not defined as an MGE.

This set of sequences were therefore used to define a set of COGs that were consistently associated with MGEs, and a set of COGs that were found within both MGE and non-MGE sequences. As transfers of sequence were identified between MGEs and PPI-1, any COG apparently characteristic of MGEs that appeared in the PPI-1 sequences displayed in Figure 2 (not all of which were found in the set of fifteen reference sequences) was reclassified as being found in both MGE and non-MGE sequence. This resulted in 584 COGs being deemed characteristic of MGEs, and 65 being found in MGE and non-MGE sequence. Furthermore, based on the Pfam domain analysis, 1,163 CDSs were identified within COGs associated with the 'Phage_integrase' domain (PF00589) characteristic of site-specific integrases that were present in fewer than a quarter of the isolates. Many of these 'rare integrase' CDSs were likely to represent MGEs not sufficiently common to be observed in the annotated set of manually curated genomes.

Description of algorithm

An algorithm was used to identify MGEs across the population that was based on two assumptions:

(1) MGEs will share a common mobilisation and integration machinery

(2) The site-specific integration of MGEs occurs at a limited number of sites and will result in normally neighbouring COGs being separated by inserted MGE COGs

Based on assumption (1), a Hidden Markov Model (HMM) was constructed such to have two states (MGE and non-MGE) and three classes of observations: class (1), COG not associated with MGE; class (2), COG sometimes associated with MGE; and class (3), COG consistently associated with MGE.

The analysis used the emission matrix ($\nu = 100$ in the described analysis):

Observation class	Probability in non-MGE	Probability in MGE
**Class 1**	$\frac{p(1)}{p(1)+p(2)} - \frac{p(3)}{2\upsilon}$	$\frac{p(1)}{\upsilon}$
**Class 2**	$\frac{p(2)}{p(1)+p(2)} - \frac{p(3)}{2\upsilon}$	$\frac{p(2)}{p(2)+p(3)} - \frac{p(1)}{2\upsilon}$
**Class 3**	$\frac{p(3)}{\upsilon}$	$\frac{p(3)}{p(2)+p(3)} - \frac{p(1)}{2\upsilon}$

Here, p(*x*) denotes the proportion of COGs in class *x*.

The transmission matrix used was:

From\To	Non-MGE	MGE
**Non-MGE**	1 - σ	σ
**MGE**	ε	1 - ε

In the described analysis, values of σ = 0.0005 and ε = 0.05 per COG were used, approximating to a null expectation of one MGE of length 20 COGs per genome. In each case, varying the parameters by an order of magnitude or more had little impact on the output of the algorithm (Supplementary Table 7). This was most simply seen in the number of strongly discordant COGs: this was the number of COGs that were in class (1) in the final iteration of the described analysis, but in class (3) in the final iteration of the run where the parameters were varied, or vice versa. Reducing ν by an order of magnitude only resulted in three strongly discordant COGs (1,133 COGs were in class (3) in the final iteration of the described analysis), and raising it by an order of magnitude resulted in just thirteen such COGs. Both reducing and increasing σ and ε by an order of magnitude resulted in even smaller changes to the final result.

Stretches of COGs determined as being in the MGE 'state' were then defined as 'putative MGEs', so long as they contained at least one COG of class (3). Based on assumption (2), in cases where the flanking CDSs on either side of a 'putative MGE' were both 'core COGs' that were separated by no more than one intervening CDS in the majority of the population, such a pair of COGs was defined as an 'insertion site'.

All 'putative MGEs' for which an 'insertion site' could not be defined were first trimmed to remove any 'core COGs' from their edges. The scaffold on which they were found was scanned for the set of 'insertion sites' already identified by the

algorithm through other ‘putative MGEs’. Where ‘insertion site’ COGs were found within a ‘putative MGE’, the MGE prediction was split. This was important in preventing multiple neighbouring MGE insertions, as observed near the origin of replication in some pneumococci (Supplementary Figure 7), being incorrectly merged into a single putative MGE.

If a ‘putative MGE’ was found to be within an ‘insertion site’ already identified by the algorithm, but only accounted for some of the CDSs between the pair of COGs comprising the ‘insertion site’, then the ‘putative MGE’ was extended outwards to the edges of the ‘insertion site’. Where multiple ‘putative MGEs’ were present within a single ‘insertion site’, they were merged into a single entity. This allowed regions of MGEs to be identified even where no sequence similarity existed with the MGEs present in the set of manually curated genomes, which was particularly useful in identifying the ends of novel MGEs. In cases where the ‘insertion site’ COGs were on different scaffolds, ‘putative MGEs’ could be extended across breaks in the assembly. This was frequently useful in the case of prophage, many of which contained a lengthy repetitive antireceptor protein CDS that often caused breaks in draft genomes.

This set of heuristics used to define the set of ‘putative MGEs’ is outlined in Supplementary Figure 29.

Following this analysis, COGs were reassigned to different classes, using both information from the 'putative MGE' annotation, and a second tier of predictions, referred to as 'candidate putative MGEs', which were defined using the rare integrase COGs identified previously. Based on assumption (2), each scaffold in which a 'rare integrase' was identified was searched for COG pairs that could form an 'insertion site', using the criteria described above. In cases where the 'insertion site' flanked the 'rare integrase', a 'candidate putative MGE' was identified. These were not included in the set of 'putative MGEs' (at least, at this stage of the analysis), but instead were regarded as potentially representing loci corresponding to MGEs likely to have inserted into a small number of isolates in the population. Hence they were provisionally included when reassigning COGs to classes:

- Any non-core class 1 or 2 COG for which at least 90% of the representatives were found to be within a 'putative MGE' or 'candidate putative MGE', or corresponded to either of the CDSs directly adjacent to a 'putative MGE', was altered to class 3
- Any non-core class 1 COG for which at least one representative was found within a 'candidate putative MGE' or a 'putative MGE', or for which more than 90% of representatives were found on the same scaffold as a 'putative MGE', was altered to class 2

The HMM analysis, and the subsequent heuristic steps, were then all repeated with the updated COG classification. The only difference was that any new 'putative MGE'

had to include a COG that was categorised as class 3 in the previous iteration, to prevent false positives arising from clusters of class 2 COGs in a non-MGE context (e.g. some alleles of PPI-1). Iterations ceased when there was no further alteration of COG classification. The parameters for the HMM were held constant over the analysis, but identical results were observed when the emission matrix was recalculated as the proportion of COGs in different classes changed in later iterations.

Output of algorithm

The described analysis (ν = 100, σ = 0.0005 COG^{-1} and ε = 0.05 COG^{-1}) converged after four iterations, with 1,133 COGs in class 3, 513 COGs in class 2, and 3,621 COGs in class 1 (Supplementary Table 6). A total of 37,670 CDSs were found within the 2,260 identified 'putative MGEs'. These 'putative MGEs' were between two and 91 COGs in length, with a median size of 31 COGs. Sixteen 'insertion sites' were identified (Supplementary Figure 7).

In order to cluster similar MGEs together, the similarities between them were calculated using the Mountford index[7], as implemented within the VEGAN R package[8]. This was selected as the distance between two identical MGEs, and between an intact MGE and a fragment corresponding to a partial assembly of the same element, would be the same. The distribution of distances was strongly discontinuous (Supplementary Figure 30), and therefore when constructing a

network all MGEs separated by a pairwise distance below 0.4 were linked by an edge. The resulting output was displayed using Cytoscape[9].

The association of COGs with particular functional domains from the Pfam database[4] was exploited in order to classify the different 'putative MGEs'. Domains associated with conjugative element machinery (MobC, TrbL, T4SS_DNA_transf), or macromolecular secretion during *Bacillus* spore formation (FtsK_SpoIIIE and SpoIIIAH), were common and appeared in sequences similar to known ICEs. In the case of prophage, domains associated with the terminase packaging enzyme (Terminase_1, Terminase_2, Terminase_3, Terminase_4, Terminase_5 and Terminase_6), or in the formation or attachment of the virion tail (Phage_tail, Sipho_tail, PhageMin_Tail, Prophage_tail, Phage_H_T_join) appeared to be reliable indicators of viral sequences. Of the thirteen multi-node network components that remained, ten contained COGs associated with the Phage_pRha functional domain, associated with MGE gene regulation; this functional domain was not present in any of the nodes predicted to be ICEs or prophage, but was found in the EF_2951 coding sequence of the *Enterococcus faecalis* phage-related chromosomal island *Ef*CIV583[10]. Seven of these ten multi-node network components also included the XhlA domain, associated with some haemolysins, although any functional inference would be misleading, as the hit was highly non-significant when corrected for multiple testing. As the XhlA domain was not found in any of the putative ICE or prophage components, one of the unclassified components in which almost all the nodes were associated with an XhlA domain was defined as containing PRCIs.

Two of the remaining four multi-node network components that shared five COGs were difficult to categorise. The larger of these corresponded to component H, which showed extensive similarity with a genomic island from *S. mutans* LJ23 (Supplementary Figure 17). Based on their length and the presence of functional domains suggesting the presence of an integrase, small terminase subunit and phage replication organiser, but the absence of any identifiable phage structural genes, these 'putative MGEs' were classified as putative PRCIs. As such, the terminase domain (Phage_Nu1) was included as a domain characteristic of PRCIs. This was only present in one other COG, found in a small number of putative ICEs, but this second hit was far less statistically significant than that in the putative PRCIs.

The two remaining multi-node network components were left unclassified. One corresponded to part of PPI-1 from SC12; these were similar to the 3' variable region of PPI-1 from *S. pneumoniae* ATCC 700669, but were associated with a 'rare integrase' that indicated they may have been recently acquired as part of an MGE. The other was a three CDS fragment found in some SC16 isolates that matched closely to part of ICE*Spn*11930[11].

A limited number of ambiguous cases were discovered that likely reflected elements that had been generated through hybridisation between MGEs, degradation of an autonomously mobile element, or a combination of both processes. One network component of five MGEs, corresponding to a short element found in SC13 and SC16,

was classified as containing prophage on the basis of a weak hit to the terminase_3 domain. However, the sequence showed greater similarity to some PRCIs, but lacked any distinctive functional domains. As this element was conserved in a clade of three SC16 isolates, it was retained in the prophage group to be conservative with regard to the rate at which MGE content changed over time (as prophage typically showed the lowest level of stability across clades). The sheer number of potential pairwise comparisons across such a diverse and poorly understood set of elements makes such manual curation impractical across the whole collection. However, such cases illustrate the importance of further experimental investigation of the different types of MGEs present in the streptococcal genus.

Ascertaining rates of change

In order to compare the relative rates at which the different MGEs spread, the 1,133 class (3) COGs were classified according to the type of MGE in which they were found. Identifying those class (3) COGs only found in one MGE type, 355 were exclusively associated with ICEs (not including ICE 'scars'), 142 were associated with PRCIs, 590 were associated with prophage (excluding the remnant) and three were associated with the prophage remnant.

The rates of change of these MGE-associated COGs across the overall phylogeny were then used to infer the rate of change in MGE content over time. The similarity metric used was the Jaccard index modified such that isolate pairs in which both members lacked any MGE-associated COGs were regarded as being as similar as

isolate pairs with identical sets of MGE-associated COGs. This metric was therefore sensitive to the diversifying effect of an MGE insertion into a previously MGE-free background. The results were qualitatively similar, but less visually informative, when using the unmodified Jaccard index.

This analysis (Figure 6) was independent of whether the COGs were identified as being within 'putative MGEs' or not. Hence differences in assembly quality between isolates, which might affect gene linkage information used in the identification of MGEs, will have had less of an effect on this purely COG-content based analysis. That isolates found to be very closely related on the basis of their core genome were found to have near-identical profiles in terms of all three MGEs independently indicates that assembly artefacts are unlikely to account for the patterns that emerge from the pairwise comparisons at higher levels of genetic divergence.

Supplementary References

1. Wyres, K. L. *et al.* Evidence of antimicrobial resistance-conferring genetic elements among pneumococci isolated prior to 1974. *BMC Genomics* **14,** 500 (2013).

2. Roberts, R. J., Vincze, T., Posfai, J. & Macelis, D. REBASE-A database for DNA restriction and modification: Enzymes, genes and genomes. *Nucleic Acids Res.* **38,** (2009).

3. Croucher, N. J. *et al.* Population genomics of post-vaccine changes in pneumococcal epidemiology. *Nat. Genet.* **45,** 656–663 (2013).

4. Punta, M. *et al.* The Pfam protein families database. *Nucleic Acids Res* **40,** D290–301 (2012).

5. Boetzer, M., Henkel, C. V, Jansen, H. J., Butler, D. & Pirovano, W. Scaffolding pre-assembled contigs using SSPACE. *Bioinformatics* **27,** 578–579 (2011).

6. Croucher, N. J. *et al.* Role of conjugative elements in the evolution of the multidrug-resistant pandemic clone *Streptococcus pneumoniae*$^{\mathrm{Spain23F}}$ ST81. *J Bacteriol* **191,** 1480–1489 (2009).

7. Mountford, M. D. An index of similarity and its application to classificatory problems. *Prog. Soil Zool.* **43,** 50 (1962).

8. Dixon, P. VEGAN, a package of R functions for community ecology. *J. Veg. Sci.* **14,** 927–930 (2003).

9. Smoot, M. E., Ono, K., Ruscheinski, J., Wang, P.-L. & Ideker, T. Cytoscape 2.8: new features for data integration and network visualization. *Bioinformatics* **27,** 431–432 (2011).

10. Matos, R. C. *et al. Enterococcus faecalis* Prophage Dynamics and Contributions to Pathogenic Traits. *PLoS Genet.* **9,** (2013).

11. Croucher, N. J. *et al.* Rapid pneumococcal evolution in response to clinical interventions. *Science* **331,** 430–434 (2011).
